# Age-, sex- and proximal–distal-resolved multi-omics identifies regulators of intestinal aging in non-human primates

**DOI:** 10.1038/s43587-024-00572-9

**Published:** 2024-02-06

**Authors:** Xinyuan Wang, Yaru Luo, Siyu He, Ying Lu, Yanqiu Gong, Li Gao, Shengqiang Mao, Xiaohui Liu, Na Jiang, Qianlun Pu, Dan Du, Yang Shu, Shan Hai, Shuangqing Li, Hai-Ning Chen, Yi Zhao, Dan Xie, Shiqian Qi, Peng Lei, Hongbo Hu, Heng Xu, Zong-Guang Zhou, Biao Dong, Huiyuan Zhang, Yan Zhang, Lunzhi Dai

**Affiliations:** 1 National Clinical Research Center for Geriatrics, Center for Immunology and Hematology and General Practice Ward/International Medical Center Ward, General Practice Medical Center, State Key Laboratory of Biotherapy, West China Hospital, Sichuan University, Chengdu, China; 2 Advanced Mass Spectrometry Center, Research Core Facility, Frontiers Science Center for Disease-Related Molecular Network, West China Hospital, Sichuan University, Chengdu, China; 3 School of Life Sciences, Tsinghua University, Beijing, China; 4 Colorectal Cancer Center, Department of General Surgery, West China Hospital, Sichuan University, Chengdu, China; 5 Department of Rheumatology and Immunology, State Key Laboratory of Biotherapy, West China Hospital, Sichuan University, Chengdu, China

**Keywords:** Proteomics, Phosphorylation, Ageing

## Abstract

The incidence of intestinal diseases increases with age, yet the mechanisms governing gut aging and its link to diseases, such as colorectal cancer (CRC), remain elusive. In this study, while considering age, sex and proximal–distal variations, we used a multi-omics approach in non-human primates (*Macaca fascicularis*) to shed light on the heterogeneity of intestinal aging and identify potential regulators of gut aging. We explored the roles of several regulators, including those from tryptophan metabolism, in intestinal function and lifespan in *Caenorhabditis elegans*. Suggesting conservation of region specificity, tryptophan metabolism via the kynurenine and serotonin (5-HT) pathways varied between the proximal and distal colon, and, using a mouse colitis model, we observed that distal colitis was more sensitive to 5-HT treatment. Additionally, using proteomics analysis of human CRC samples, we identified links between gut aging and CRC, with high HPX levels predicting poor prognosis in older patients with CRC. Together, this work provides potential targets for preventing gut aging and associated diseases.

## Abstract (web summary)

Using a multi-omics approach, Wang et al. explored sex-specific and region-specific patterns of intestinal aging in non-human primates, identifying regulators with conserved functions in *Caenorhabditis* *elegans* intestinal aging, in colitis in mice and in patient colorectal cancer samples.

## Main

According to the embryonic origins, the region from the cecum to the proximal two-thirds of the transverse colon originating from the midgut is called right-sided colon (proximal colon), and the part from the distal one-third of the transverse colon to the rectum originating from the hindgut is called left-sided colon (distal colon). The integrity of barrier, a hallmark of gut health^1^, relies on the preservation of the intestinal epithelium, a barrier against pathogens^2–5^. Destruction of this barrier may increase the risk of disorders, including inflammatory bowel disease (IBD), colorectal cancer (CRC)^6,7^, chronic liver disease^8^, diabetes^9,10^ and obesity^11^. Interestingly, although the proximal and distal colon belong to the same organ, the incidence of many diseases differs between the two locations. For example, left-sided colonic mucosal inflammation is present in 50–80% of patients with ulcerative colitis (UC)^12^. Among all diagnosed patients with CRC, 53.9% have cancer located on the left side; 32.2% have cancer on the right side; and 13.9% have an unknown primary site^13^. Moreover, patients with right-sided CRC often have microsatellite instability (MSI)-high tumors, whereas patients with left-sided CRC tend to have chromosomal instability (CIN)-high tumors and a better prognosis than patients with right-sided CRC^14,15^.

Aging substantially contributes to gut dysfunction, leading to prevalent intestinal disorders in individuals over 40 years of age^6,16,17^. Intestinal aging involves morphological changes, slowed peristalsis, decreased absorptive capacity, impaired gut epithelial regeneration and dysbiosis^18,19^. Cutting-edge omics technologies provide robust avenues for delving into the root causes of gut aging and have unveiled age-dependent epigenetic alterations^20^, differentiation patterns of intestinal stem cells (ISCs)^18,21^, enteric nervous system dysfunction^22^ and the pivotal role of the gut microbiome in modulating healthy aging^23^. Despite tremendous progress, substantial tasks remain. First, an integrative omics analysis of normal gut tissues from diverse age groups of higher animals is crucial for revealing intestinal aging mechanisms. Second, dissimilarities in disease types and onset between the proximal and distal colon suggest aging heterogeneity. However, this assumption still lacks evidence. Third, the specific impacts of age-related molecules on gut aging remain unclear, offering potential targets for preventing intestinal aging and associated disorders.

*Macaca fascicularis* is a near-human species that is widely used as an aging model^24–33^. In the present work, by performing location-specific and sex-resolved multi-omics analyses of 104 intestinal tissues taken from 26 *M. fascicularis* animals of different ages, we demonstrated the heterogeneity of aging between the proximal and distal colon in males and females and identified crucial regulators of gut aging. In addition, we identified a number of proteins potentially linking gut aging to CRC.

## Multi-omics characterization of large intestinal aging

**Fig. 1:**
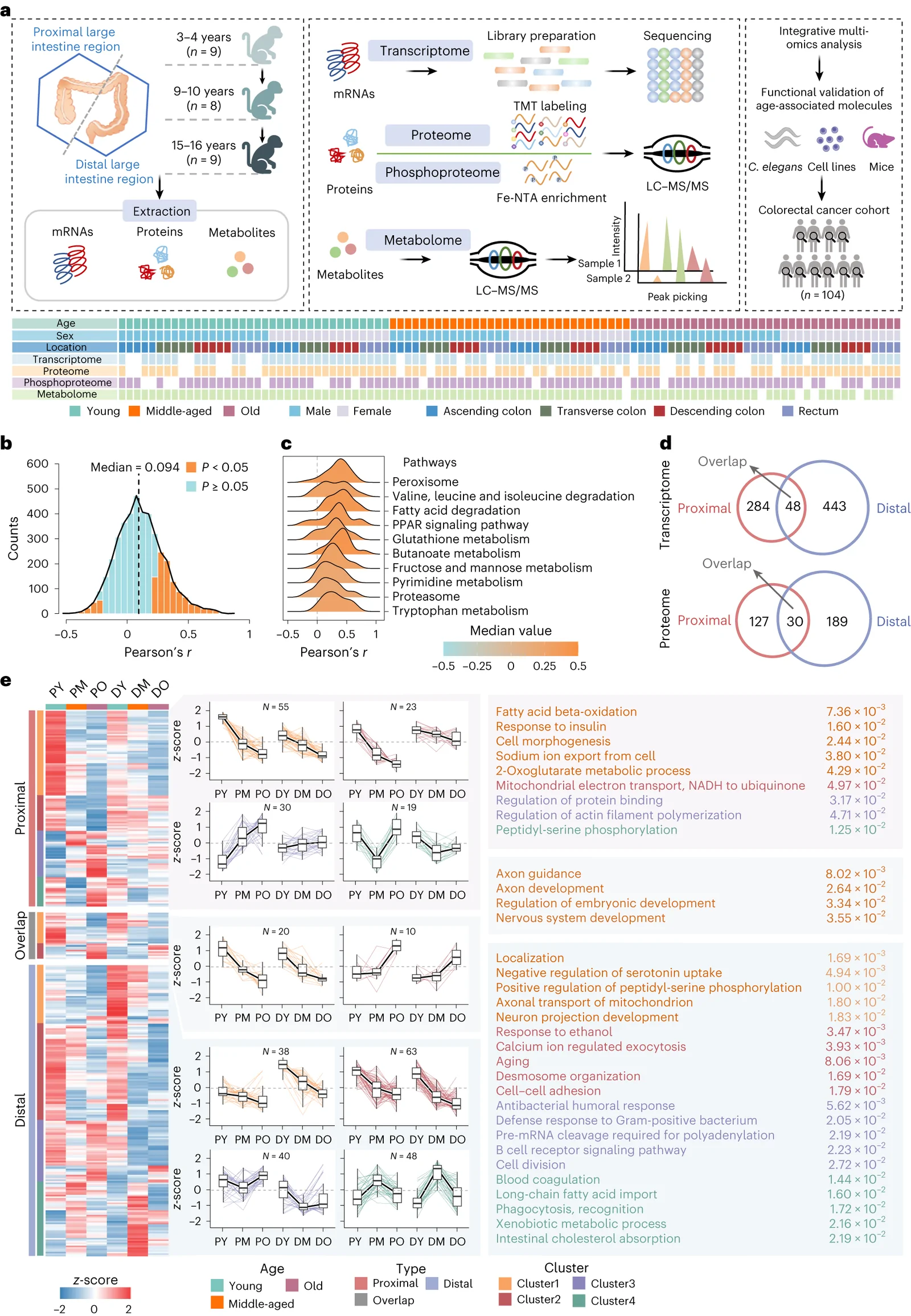
Location-resolved proteome of large intestinal aging. **a**, Schematic of multi-omics analyses of large intestinal tissues from 26 *M. fascicularis* animals. **b**, Histogram of correlations between proteins and mRNAs. Significantly correlated genes are shown in orange (*n* = 77 samples, *P* < 0.05, Pearson’s correlation analysis). **c**, Top 10 enriched KEGG pathways using genes with positive mRNA-to-protein correlations (*P* < 0.05, Fisher’s exact test). **d**, Venn diagrams displaying overlapping age-related mRNAs/proteins between the proximal and distal colon (*P* < 0.05, one-way ANOVA). **e**, Unsupervised *k*-means clustering analysis of age-related proteins. The expression patterns of the age-related proteins in distinct clusters are shown. *z*-scored protein levels were mapped along three age stages (young, middle age and old) in the proximal and distal colon (*P* < 0.05, one-way ANOVA, median ± quartiles; ‘*N*’ denotes protein number). The top five enriched pathways in each cluster are shown in the right panel. PY, proximal samples from young; PM, proximal samples from middle age; PO, proximal samples from old; DY, distal samples from young; DM, distal samples from middle age; DO, distal samples from old. Raw and processed data for drawing are provided in Source Data File 1. Source data

**Extended Data Fig. 1:**
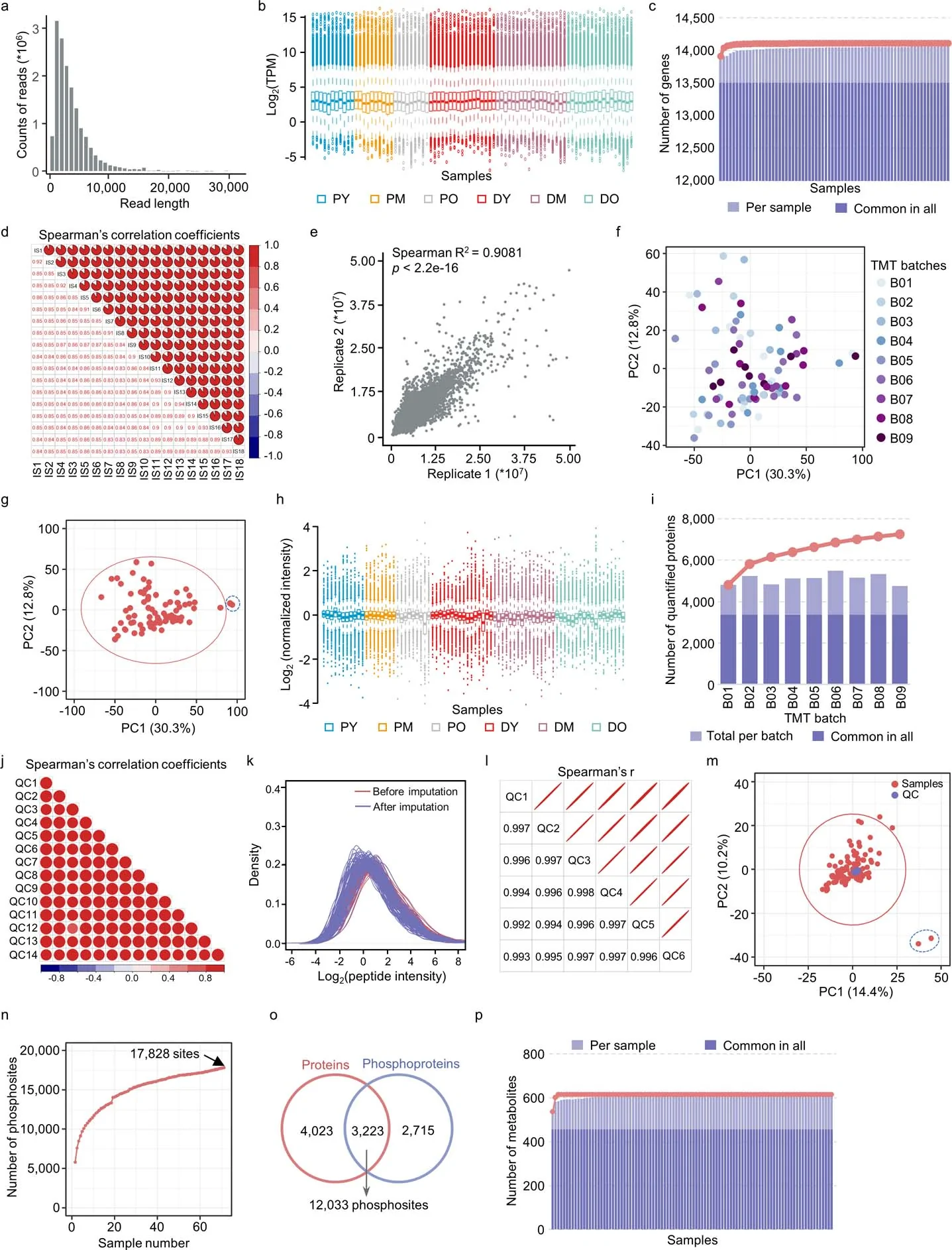
Quality evaluation of transcriptome, proteome, phosphoproteome and metabolome data. **a**, Read length distribution of all identified genes. **b**, Boxplots showing log2-transformed transcripts per million (TPM) of mRNAs (n = 14,113 genes) in 82 samples (median ± quartiles), including PY (proximal samples from young, n = 11), PM (proximal samples from middle age, n = 10), PO (proximal samples from old, n = 9), DY (distal samples from young, n = 17), DM (distal samples from middle age, n = 18), and DO (distal samples from old, n = 17). **c**, Histogram showing identified mRNA number. **d,e**, Spearman’s correlation for common reference samples (**d**) and overlapping proteins in technical duplicate (n = 80 biologically independent samples, *p* < 2.2e-16) (**e**). **f**, Principal component analysis (PCA) of proteins quantified in 9 batches. **g**, PCA plot of the proteome. Red dots indicate 80 biologically independent samples, and blue circled dots represent outliers. **h**, Boxplots showing log2-transformed normalized intensity of identified proteins (n = 7,246) in 78 samples (median ± quartiles). PY (n = 11), PM (n = 8), PO (n = 9), DY (n = 16), DM (n = 16), and DO (n = 18). **i**, Histogram showing identified protein number. **j**, Spearman’s correlation for 14 quality control (QC) samples for phosphoproteome. **k**, Density plot displaying phosphopeptide intensity distribution before (red) and after (blue) imputation. **l**, Spearman’s correlation for 6 QC samples for metabolome. **m**, PCA plot of metabolome. Red dots represent 98 biologically independent samples, blue dots represent 6 QC samples, and blue discrete dots circled samples are excluded. **n**, Cumulative number of identified phosphosites (n = 88 biologically independent samples). **o**, Venn diagram showing the overlap between phosphoproteins and proteins. **p**, Histogram showing identified metabolite number. Raw and processed data for drawing are provided in Source Data File 8. Source data.

To determine the molecular landscape of large intestinal aging, we collected 104 pieces of fresh large intestinal tissues from 26 *M. fascicularis* animals and performed age-resolved, location-resolved and sex-resolved multi-omics analyses, including transcriptomics, proteomics, phosphoproteomics and metabolomics. Of the 26 monkeys, nine were young (3–4 years old, five males and four females); eight were middle-aged (9–10 years old, four males and four females); and nine were aged (15–16 years old, five males and four females) (Fig. 1a). RNA sequencing identified 14,113 expressed genes with average transcript per million (TPM) values above 1 (Extended Data Fig. 1a–c). Isobaric tandem mass tag (TMT)-based proteomic profiling was carried out in duplicate. Peptide and protein identifications were mapped to a reference database of *M. fascicularis* with 30,202 entries^34^. The reproducibility of inter-plex common reference and replicate samples demonstrated the high quality of the proteomics data (Extended Data Fig. 1d–h). As a result, 7,246 proteins were identified with at least two confident unique peptides (Extended Data Fig. 1i). Label-free phosphoproteomics and untargeted metabolomics were performed with continuous quality controls (QCs) every 21 and 20 MS injections, respectively (Extended Data Fig. 1j–m). Across the datasets, appropriate filtering resulted in the identification of 17,828 phosphosites on 5,938 proteins with a localization probability greater than 0.75 (Extended Data Fig. 1n,o) and 615 metabolites with QC coefficients of variation (CVs) less than 0.3 (Extended Data Fig. 1p).

## Multi-omics reveals the heterogeneity of intestinal aging

**Extended Data Fig. 2:**
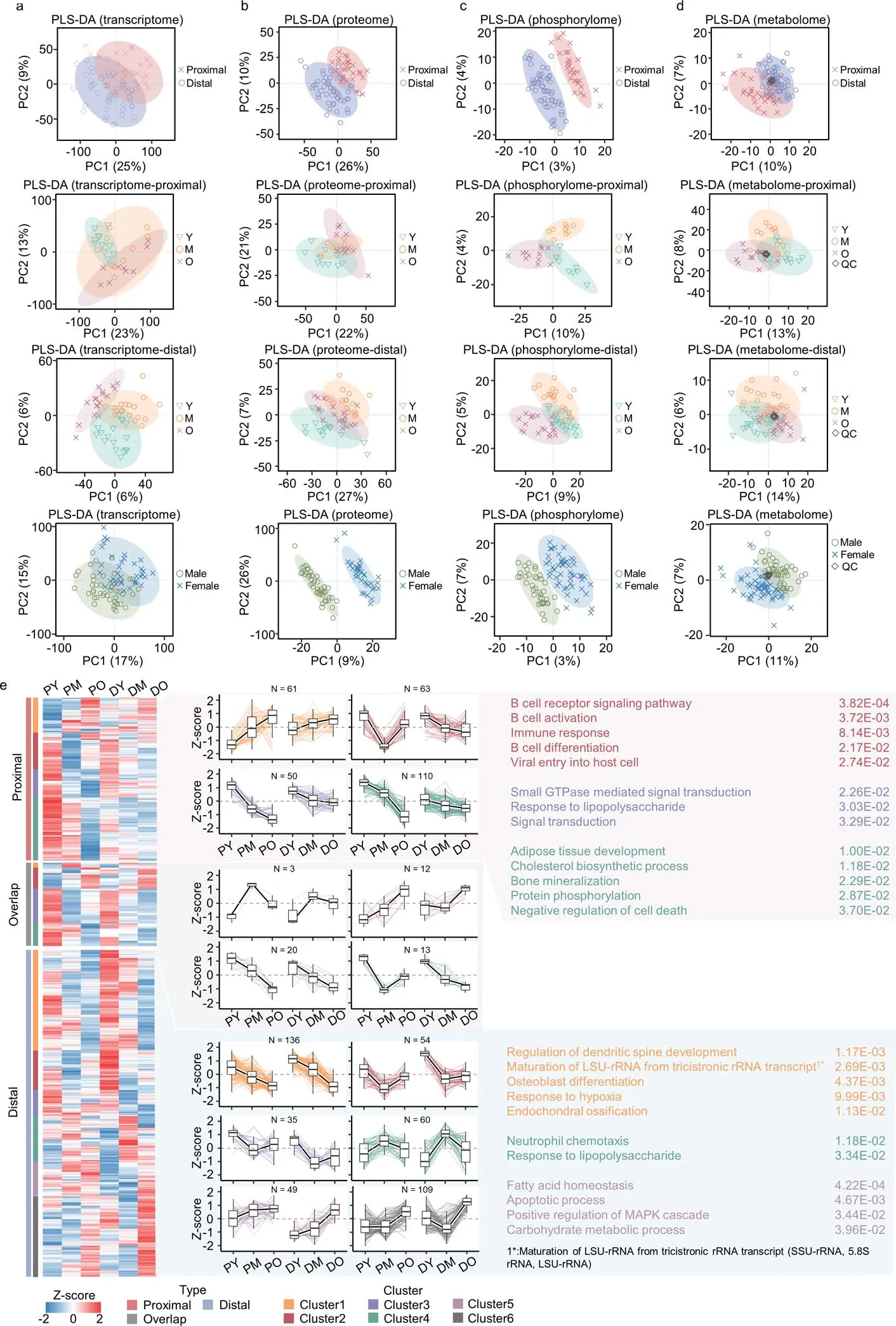
Partial least squares discriminant analyses (PLS-DA) of the omics data in the large intestinal tissues and location-resolved transcriptome of large intestinal aging. **a-d**, PLS-DA of the large intestinal tissues from 26 *Macaca fascicularis* based on transcriptomic (**a**), proteomic (**b**), phosphoproteomic (**c**) and metabolomic data (**d**). **e**, Unsupervised *k*-means clustering analysis of age-related mRNAs among all samples (*p* < 0.05, one-way ANOVA). The expression patterns of the age-related mRNAs in distinct clusters are shown in the middle panel. Their scaled expression patterns were mapped across three age stages (young, middle age and old) in the proximal and distal colon. ‘N’ represents the number of genes in each cluster. The top 5 enriched pathways for each cluster are shown on the right (*p* < 0.05, Fisher’s exact test). Raw and processed data for drawing are provided in Source Data File 9. Source data

Partial least squares discriminant analyses (PLS-DA) effectively distinguished the tissue samples across age, location and sex dimensions (Extended Data Fig. 2a–d), underscoring the necessity of considering both location-related and sex-related variations with age in subsequent data interpretation. Moderate correlations between protein and mRNA expression were observed in normal large intestinal tissues^35^ (Fig. 1b), and certain metabolic processes, such as valine, leucine and isoleucine degradation, fatty acid degradation and tryptophan (Trp) metabolism, showed significant positive protein–mRNA correlations (Fig. 1c).

**Extended Data Fig. 3:**
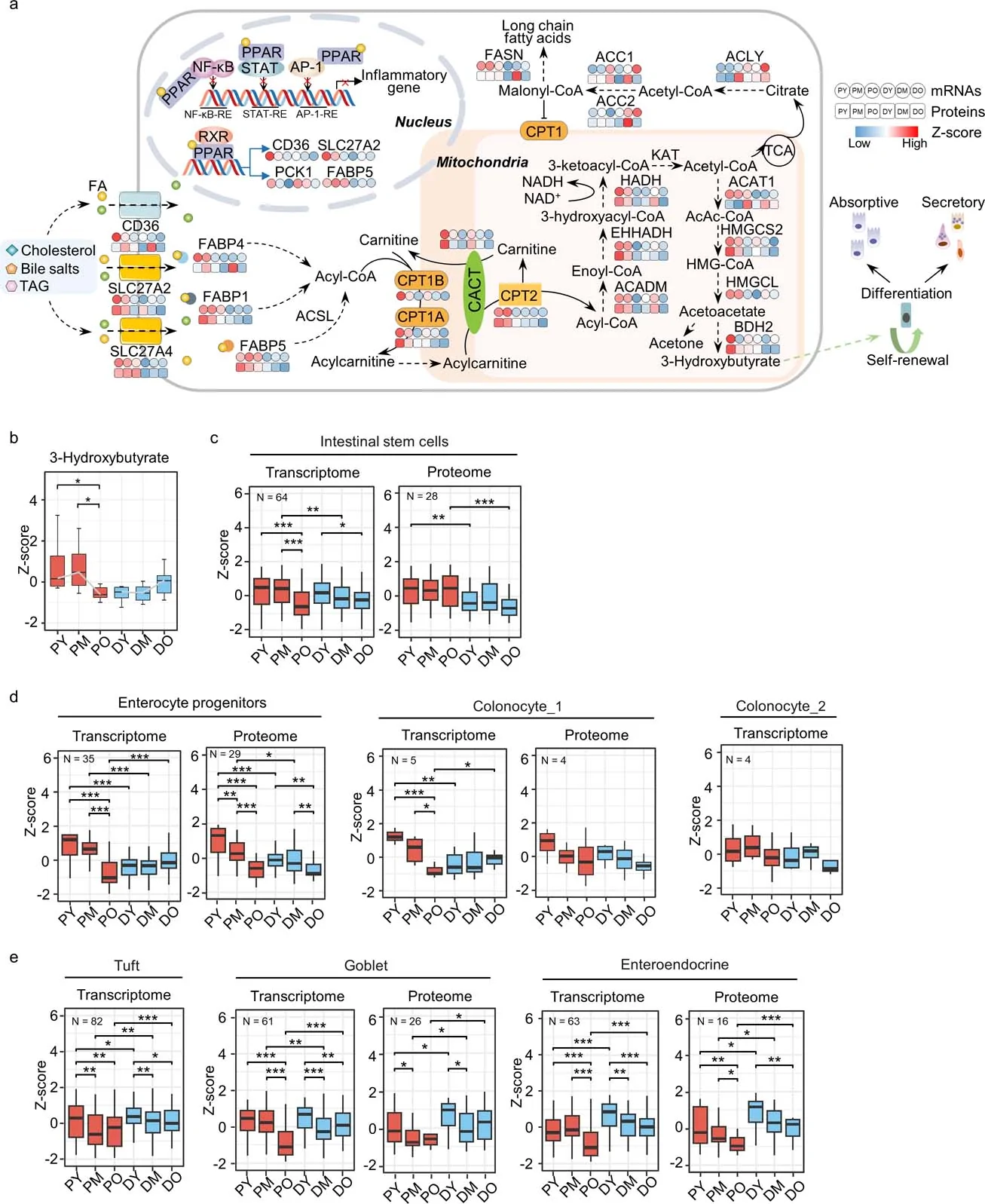
Changes in the fatty acid β-oxidation pathway, ketogenesis and intestinal cell populations during aging. **a**, Schematics of fatty acid β-oxidation and ketogenesis. The scaled expression patterns of mRNAs/proteins were mapped across three age stages (young, middle age and old) in the proximal and distal colon. Circles represent mRNA, and squares represent proteins. **b**, Box plot displaying the levels of 3-hydroxybutyrate mapped across three age stages (young, middle age and old) in the proximal and distal colon (n = 3 samples per group, *p* = 0.026 (PY *vs* PO), *p* = 0.026 (PM *vs* PO), Wilcoxon signed-rank sum test, mean ± quartiles). **c-e**, Box plots displaying the expression patterns of the characteristic genes of cell populations in the transcriptome and proteome to roughly reflect the changes in cell numbers. The scaled expression patterns of characteristic genes in cell populations, including intestinal stem cells (**c**), absorptive enterocyte progenitors and downstream enterocytes (colonocyte_1 and colonocyte_2) (**d**), secretory cell populations including goblet cells, enteroendocrine cells and tuft cells (**e**), were mapped across three age stages (young, middle-aged and old) in the proximal and distal colon (two-sided Student’s *t* test, mean ± quartiles), *p < 0.05, **p < 0.01, and ***p < 0.001. The numbers of characteristic genes or proteins in these cell populations are shown in the figures. Raw and processed data for drawing are provided in Source Data File 10. Source data

To minimize the individual animal effects, we balanced the samples for each comparative analysis by using the same number of samples chosen from each animal. After strict balancing, age-associated mRNAs and proteins were pinpointed in both the proximal and distal colon across the young, middle-aged and old groups, without considering sex effects (*P* < 0.05, one-way ANOVA). Intriguingly, a greater number of age-associated gene products were observed in the distal colon (Fig. 1d). Unsupervised *k*-means clustering analyses demonstrated similar trends but different degrees of changes in age-associated proteins and mRNAs in both colonic segments (Fig. 1e and Extended Data Fig. 2e). Some proteins exhibited significant changes in middle age that were reversed in old age, such as proteins in cluster3 and cluster4 in the distal colon (Fig. 1e), suggesting potential adaptive mechanisms optimizing function during this specific life stage. For example, serum cholesterol levels tend to rise with age until middle age and then decrease^36–38^, and a similar pattern of trends in the intestinal cholesterol absorption pathway was observed (Fig. 1e). Fatty acid β-oxidation (FAO) is a mitochondrial process that promotes ISC renewal^39,40^. Higher levels of FAO-associated transporters and enzymes, such as CPT1A, CPT1B, CPT2, EHHADH and BDH2, were observed in the proximal colon across all age stages (Extended Data Fig. 3a), and their expression decreased faster with age (Fig. 1e and Extended Data Fig. 3a). 3-Hydroxybutyrate, a pivotal FAO and ketogenesis product critical for regulating ISC renewal and differentiation, exhibited a decline in the proximal colon of old individuals (Extended Data Fig. 3b). Based on the levels of characteristic genes^41^ and the described algorithm^42^, we observed age-related drops in ISC numbers (Extended Data Fig. 3c), enterocyte progenitors and downstream absorptive colonocytes (colonocyte_1 and colonocyte_2), particularly notable in the proximal colon (Extended Data Fig. 3d), aligning with previous mouse data^43^. 3-Hydroxybutyrate, which facilitates ISC differentiation into absorptive cells, might contribute to these trends^44^, but its influence on ISC differentiation into secretory cells remains debatable^21,40,44^. The age-related reductions extended to secretory cell counts, including goblet cells, enteroendocrine cells and tuft cells, predominantly in the proximal colon (Extended Data Fig. 3e). Although trends in enteroendocrine and tuft cells matched previous results^43^, alterations in goblet cells differed from mouse data^43^ but aligned with the findings of other studies^21,45^. Notably, the potential sample variations, sample size and characteristic gene number might impact the statistical accuracy of our study and mouse single-cell research^43^. These findings suggest that the homeostasis of the proximal colon may be more dependent on FAO and ketogenesis.

**Extended Data Fig. 4:**
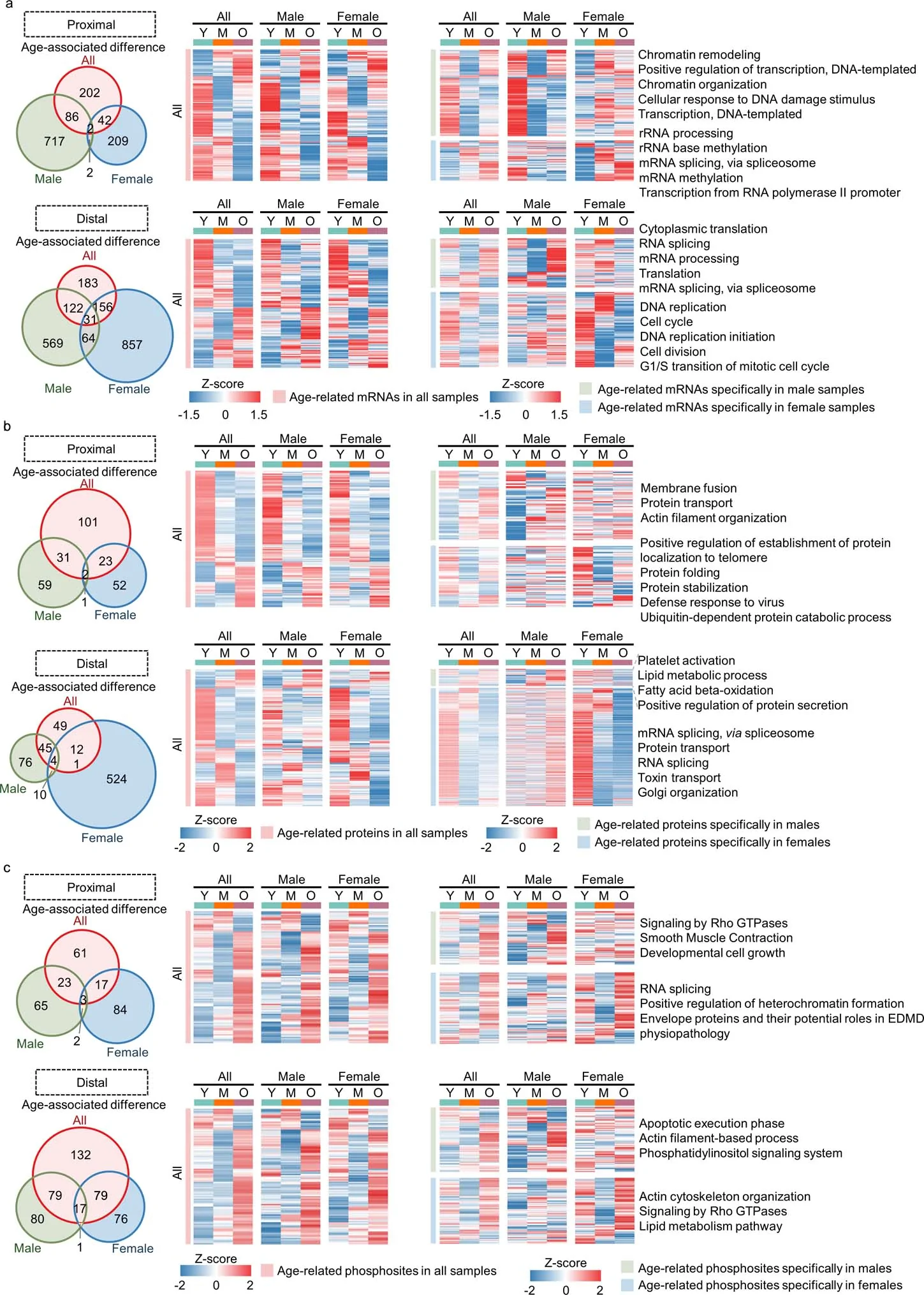
Location- and sex-resolved transcriptome, proteome and phosphoproteome of large intestinal aging. **a**, Venn diagrams illustrating age-related mRNA number in the proximal (upper) and distal (below) region among all, male, and female samples. Red circle represents age-related mRNAs screened by using all samples, green circle represents age-related mRNAs in males, and blue circle represents the age-related mRNAs in females (*p* < 0.05, one-way ANOVA). Heatmaps on the right present the levels of age-related mRNAs obtained from different screening conditions. The enriched pathways using sex-specific mRNAs are presented on the right side of the heatmaps (*p* < 0.05, Fisher’s exact test). **b**, Venn diagrams illustrating age-related protein number in the proximal region (upper) and the distal region (below) among all, male, and female samples. Red circle represents age-related proteins screened by using all samples, green circle represents age-related proteins in males, and blue circle represents age-related proteins in females (*p* < 0.05, one-way ANOVA). Heatmaps on the right show the expression of age-related proteins obtained from different screening conditions. The enriched pathways using sex-specific proteins are presented on the right side of the heatmaps (*p* < 0.05, Fisher’s exact test). **c**, Venn diagrams illustrating age-related phosphosite number in the proximal region (upper) and the distal region (below) among all, male, and female samples. Red circle represents age-related phosphosites screened by using all samples, green circle represents age-related phosphosites in males, and blue circle represents age-related phosphosites in females (*p* < 0.05, one-way ANOVA). Heatmaps on the right present the levels of age-related phosphosites obtained from different screening conditions. The enriched pathways using proteins corresponding to these sex-specific phosphosites are presented on the right side of the heatmaps (*p* < 0.05, Fisher’s exact test). Raw and processed data for drawing are provided in Source Data File 11. Source data

Additionally, the impact of sex on gene expression during large intestinal aging was explored (Extended Data Fig. 4a,b). Notably, more age-associated gene products were found in the proximal and distal colon of males and females, respectively (Extended Data Fig. 4a,b). Genes with age-related changes in both sexes were associated with gene expression regulation and biomolecule synthesis pathways (Extended Data Fig. 4a,b). Specifically, at the mRNA level, aging affected the transcriptional regulation in the proximal colon of males and RNA synthesis in females (Extended Data Fig. 4a). In contrast, males exhibited altered RNA synthesis in the distal colon, whereas females encountered impacts on DNA replication and the cell cycle (Extended Data Fig. 4a). Analysis of age-related proteins revealed sex-specific enrichments, including protein folding, stabilization and localization for females in the proximal colon and protein transport for males (Extended Data Fig. 4b). Notably, significant age-related changes in the FAO pathway were observed in the distal colon of males (Extended Data Fig. 4b).

**Fig. 2:**
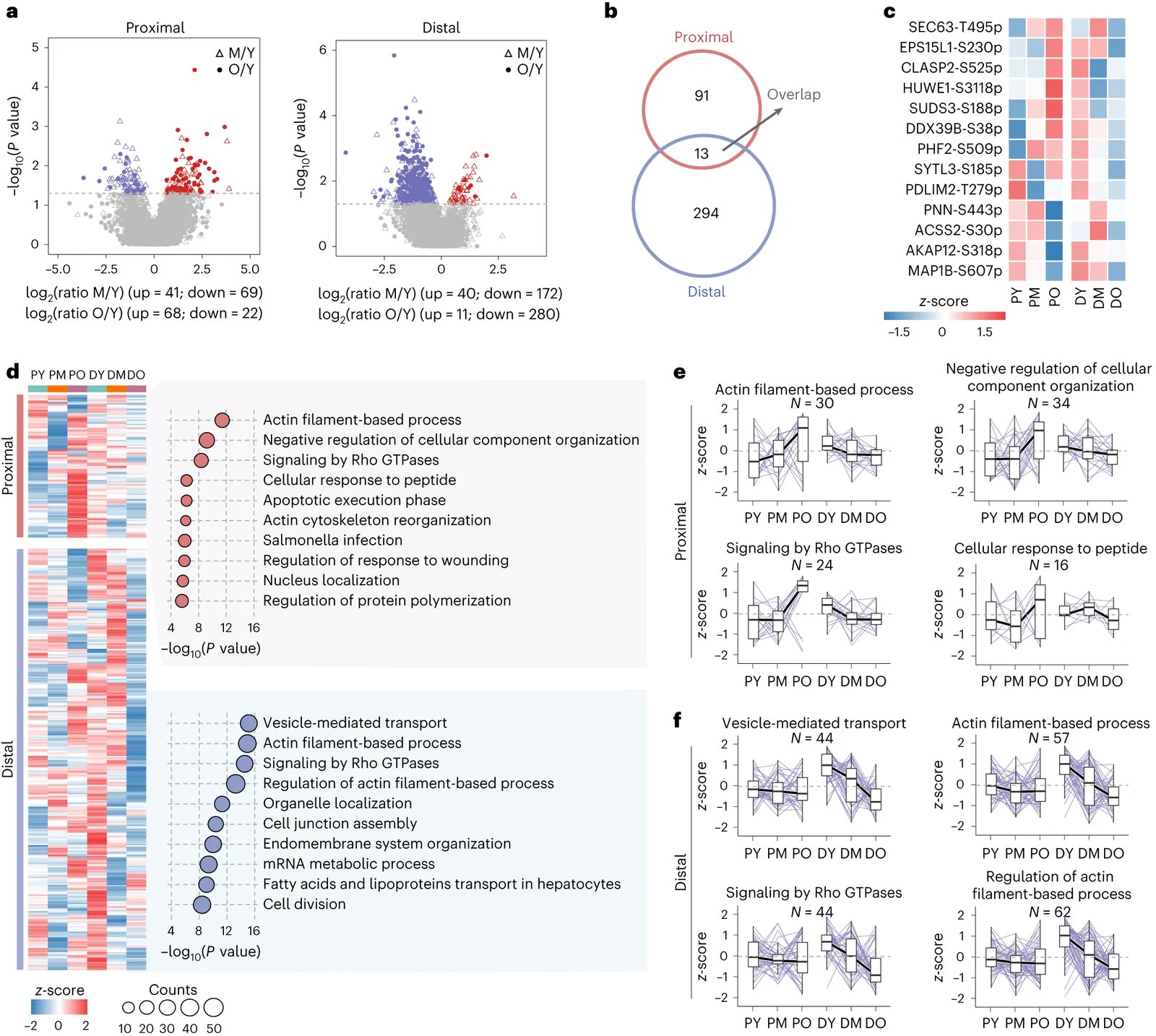
Location-resolved phosphoproteome of large intestinal aging. **a**, Volcano plots displaying phosphosites differentially expressed between middle-aged and young individuals and between old and young individuals in the proximal and distal colon (*P* < 0.05, two-sided Student’s *t*-test). Red and blue dots represent upregulated and downregulated phosphosites, respectively. **b**, Venn diagram displaying the overlapping age-related phosphosites between the proximal and distal colon (*P* < 0.05, one-way ANOVA). **c**, Heat map showing the levels of 13 common age-related phosphosites in the proximal and distal colon. **d**, Heat map showing the levels of age-related phosphosites in the proximal and distal colon. The pathways enriched by Metascape using age-related phosphoproteins are shown in the right panel (*P* < 0.05, Fisher’s exact test). **e**,**f**, Box plots displaying the levels of phosphosites in representative enriched pathways in the proximal (**e**) and distal (**f**) colon (median ± quartiles; whiskers extend to minimum and maximum values, and ‘*N*’ denotes phosphosite number). Their expression patterns were mapped across three age groups (young, middle age and old) in the proximal (*n* = 3 samples per stage) and distal (*n* = 6 samples per stage) colon. Raw and processed data for drawing are provided in Source Data File 2. PY, proximal samples from young; PM, proximal samples from middle age; PO, proximal samples from old; DY, distal samples from young; DM, distal samples from middle age; DO, distal samples from old. Source data

Protein post-translational modifications (PTMs), such as phosphorylation, tightly regulate the aging process^46,47^. Strikingly, numerous phosphosites exhibited contrasting age-related trends in the proximal and distal colon. In aged individuals, we noted a greater decrease in phosphosites in the distal colon and a higher number of increased phosphosites in the proximal colon (Fig. 2a). Notably, most age-associated phosphosites across three age stages in the proximal and distal colon were different (*P* < 0.05, one-way ANOVA) (Fig. 2b), with only 13 sites overlapping (Fig. 2c). Unsupervised *k*-means clustering analyses showed that age-related phosphosites in the proximal colon slightly decreased in middle age, followed by an increase in old age, whereas those in the distal colon exhibited a consistent decrease with age (Fig. 2d–f). The proteins bearing these phosphosites were mainly associated with signaling by Rho GTPases and actin filament-based processes (Fig. 2d–f), suggesting the involvement of phosphorylation in cell–cell junction regulation. Like the above proteomic findings, age-related phosphosites, when analyzed without considering sex influence, exhibited similar trends across both sexes in the proximal and distal colon. However, the examination of sex-specific age-associated phosphosites highlighted sex-dependent features (Extended Data Fig. 4c) and revealed their association with Rho GTPase signaling, actin filament-based processes and actin cytoskeleton organization (Extended Data Fig. 4c), underscoring the intricate interplay between protein phosphorylation and cell–cell junction regulation.

**Fig. 3:**
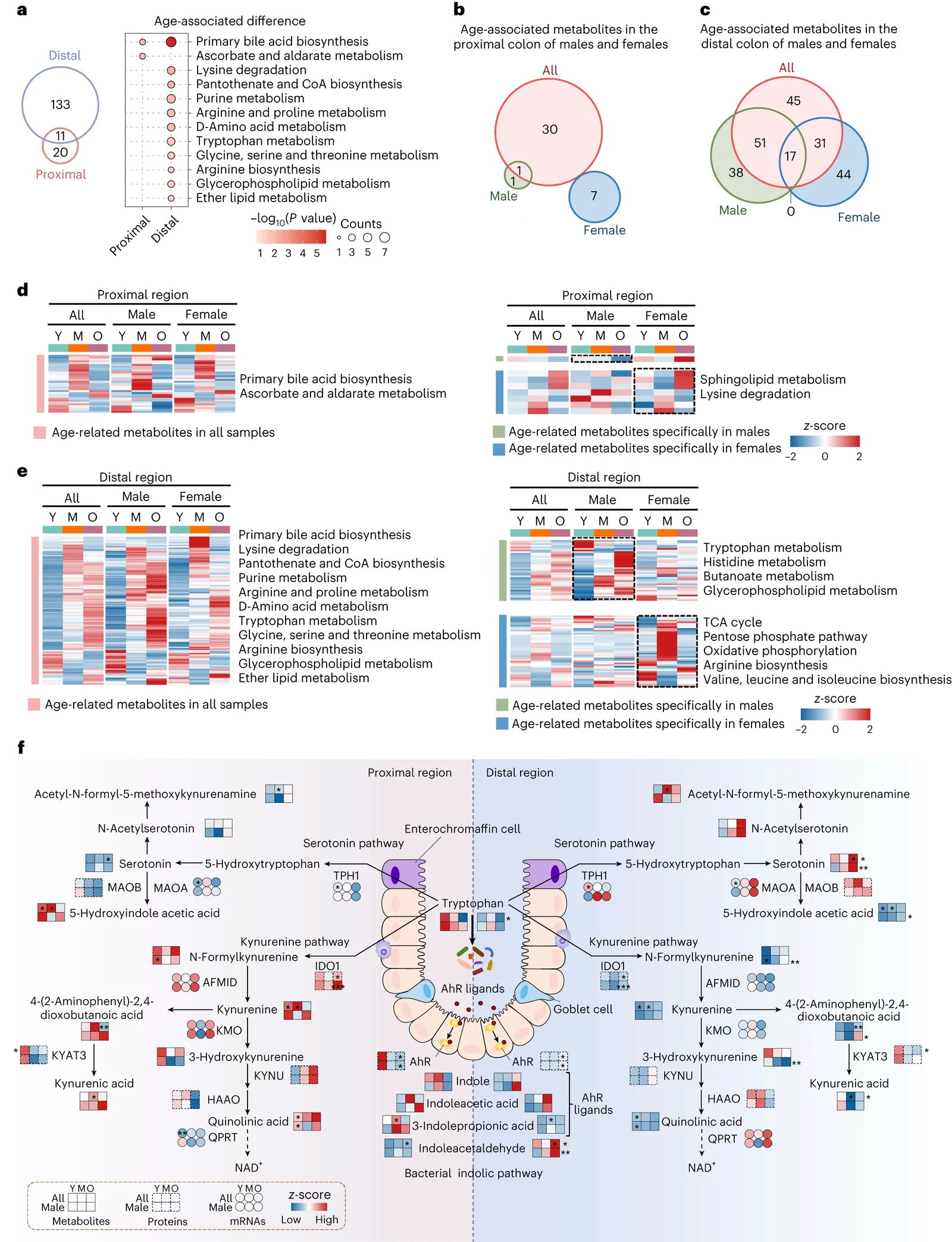
Location-resolved and sex-resolved metabolome of large intestinal aging. **a**, Venn diagram displaying overlapping age-related metabolites in the proximal and distal colon (*P* < 0.05, Kruskal–Wallis test). The enriched metabolic pathways are shown on the right. Circle size indicates the number of metabolites enriched in the pathway. Color indicates −log_10_(*P* value) (*P* < 0.05, Fisher’s exact test). **b**,**c**, Venn diagrams illustrating age-related metabolite number in the proximal (**b**) and distal (**c**) colon among all, male and female samples. Red circle represents age-related metabolites identified in all samples; green circle represents age-related metabolites identified in males; and blue circle represents age-related metabolites identified in females (*P* < 0.05, Kruskal–Wallis test). **d**,**e**, Heat map showing the expression levels of age-related metabolites in the proximal (**d**) and distal (**e**) colon in each group, and pathways associated with these metabolites are presented on the right side of the heat map (*P* < 0.05, Fisher’s exact test). **f**, Levels of enzymes and metabolites in the Trp metabolism pathway in the proximal and distal colon. Test methods for differences between the proximal and distal colon in certain age stages: mRNA, two-sided Student’s *t*-test; protein, two-sided Student’s *t*-test; and metabolite, Wilcoxon rank-sum test. The test significance is labeled in the box or circle. Test methods for differences across three age groups in the proximal or distal colon: mRNAs, one-way ANOVA; proteins, one-way ANOVA; and metabolites, Kruskal–Wallis test. The *P* value is labeled outside the box or circle. *P < 0.05, **P < 0.01 and ***P < 0.001. Raw and processed data for drawing are provided in Source Data File 3. M, middle-aged; O, old; Y, young. Source data

The metabolic imbalances associated with large intestinal aging are poorly understood. In pursuit of insights into metabolic disturbances during large intestine aging, age-associated metabolites across three age stages were identified in the proximal and distal colon (*P* < 0.05, Kruskal–Wallis test). Consistent with the transcriptomic, proteomic and phosphoproteomic findings, a greater count of age-linked metabolites emerged in the distal colon (Fig. 3a). Our investigation disclosed age-related variations in primary bile acid biosynthesis, along with shifts in ascorbate and aldarate metabolism in the proximal colon. Similarly, primary bile acid biosynthesis exhibited age-related differences in the distal colon (Fig. 3a). Additionally, we identified sex-specific age-linked metabolites in the proximal and distal colon (Fig. 3b,c). Pathway enrichment analysis highlighted distinct regulatory patterns of specific metabolic pathways associated with age in the proximal and distal colon for both males and females (Fig. 3d,e). Importantly, Trp metabolism was exclusively enriched in the distal colon in males (*P* = 0.0008, Fisher’s exact test). Trp metabolism generally adopts the kynurenine (KYN) or serotonin (5-HT) pathway, with about 90% of Trp consumed through the KYN pathway in the large intestine^48^. Surprisingly, we observed greater activity in the 5-HT pathway in the distal region, whereas the proximal region favored the KYN pathway (Fig. 3f).

**Extended Data Fig. 5:**
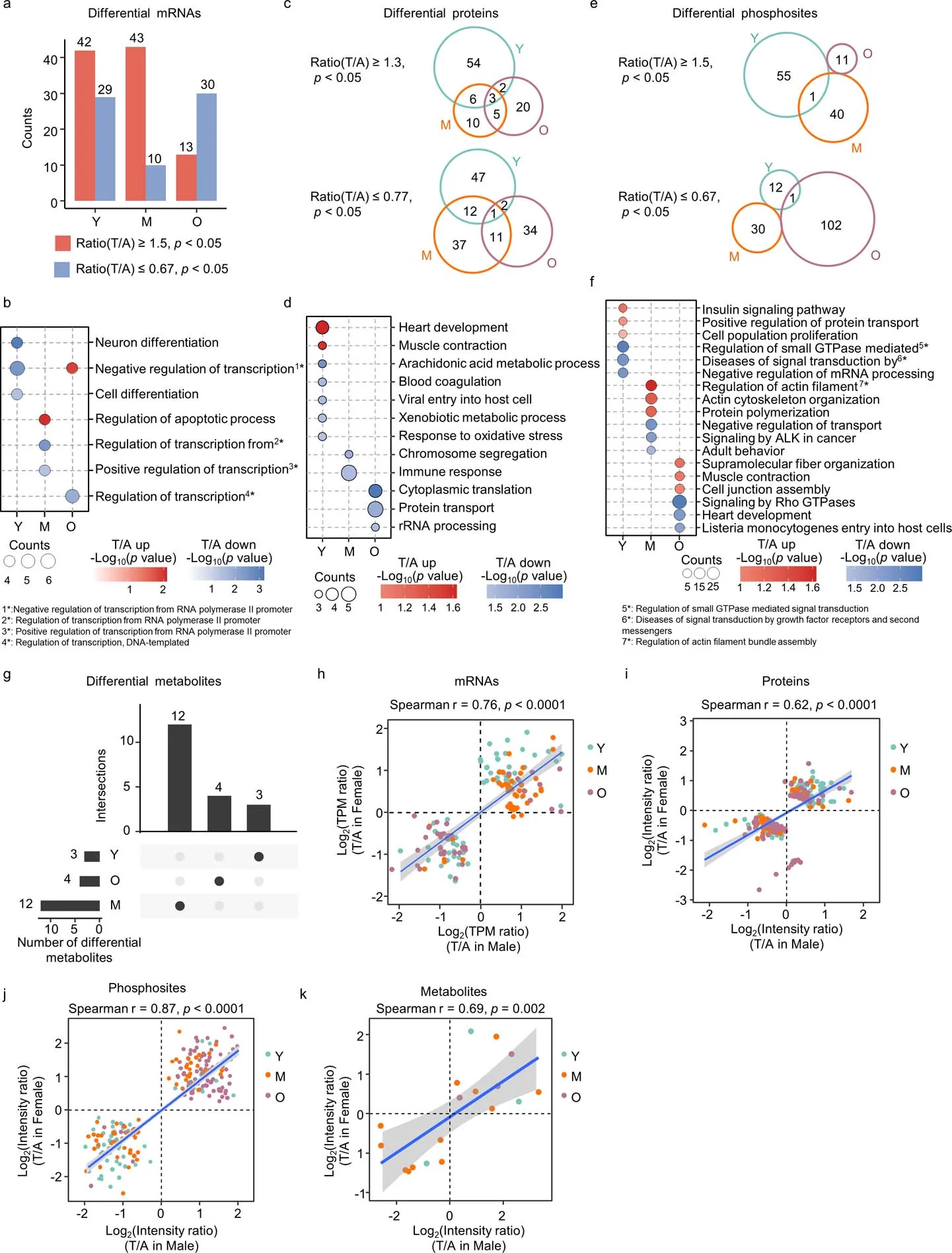
Comparative omics analysis between the ascending and transverse colon. **a**, Histogram showing differentially expressed mRNA number between the ascending (A) and transverse (T) colon at different age stages (*p* < 0.05, two-tailed paired *t* test). Red and blue represent mRNAs with a ratio (T/A) ≥ 1.5 and ≤ 0.67, respectively. **b**, Bubble plot displaying the upregulated and downregulated pathways. Circle size indicating mRNA number. Red and blue represent −log_10_(*p* value) (*p* < 0.05, Fisher’s exact test). **c**, Venn diagram illustrating differentially expressed protein number between A and T at different age stages (*p* < 0.05, two-tailed paired *t* test). The upper and lower parts represent proteins with a ratio (T/A) ≥ 1.3 and ≤ 0.77, respectively. **d**, Bubble plot displaying the upregulated and downregulated pathway. Circle size indicating protein number. Red and blue represent −log_10_(*p* value) (*p* < 0.05, Fisher’s exact test). **e**, Venn diagram illustrating differential phosphosite number between A and T at different age stages (*p* < 0.05, two-tailed paired *t* test). The upper and lower parts represent phosphosites with a ratio (T/A) ≥ 1.5 and ≤ 0.67, respectively. **f**, Bubble plot displaying the top 3 enriched pathways in each age group. Circle size representing phosphoprotein number. Circle color represents −log_10_(*p* value) (*p* < 0.05, Fisher’s exact test). **g**, UpSet diagram showing differential metabolite number between A and T at different age stages (*p* < 0.05, Wilcoxon signed-rank paired test). **h-k**, Spearman’s correlation for differential mRNAs (**h**), differential proteins (**i**), differential phosphosites (**j**) and differential metabolites (**k**) in different age stages using log_2_(ratio(T/A)) in males and females. Green points represent young group, orange points represent middle age group, and purple points represent old group. Raw and processed data for drawing are provided in Source Data File 12. Source data

Additionally, we explored the molecular distinctions between the ascending and transverse colon. In the transverse colon of young individuals, reduced neuron differentiation, negative transcriptional regulation and cell differentiation were observed, contrasting with elevated negative transcription regulation in old individuals (Extended Data Fig. 5a,b). Moreover, the number of differentially expressed proteins varied between the two colonic segments in young individuals, with lower levels of proteins responsible for protein transport in the transverse colon of old individuals (Extended Data Fig. 5c,d). Phosphosites with higher levels in the transverse colon decreased with age, whereas those with lower levels increased. The differential phosphosites between colonic segments exhibited minimal overlap across age stages, with notable differences in the phosphosites in cell junction assembly and signaling by Rho GTPase related to cell–cell adhesion regulation (Extended Data Fig. 5e,f). Limited differential metabolites suggested minor metabolic profile variations between the ascending and transverse colon (Extended Data Fig. 5g). Sex minimally impacted the molecular profiles, as indicated by correlation analysis across different age stages (Extended Data Fig. 5h–k).

Collectively, the above findings underscore the heterogeneity of large intestinal aging at the molecular level.

## Effects of age-related genes on gut atrophy

**Fig. 4:**
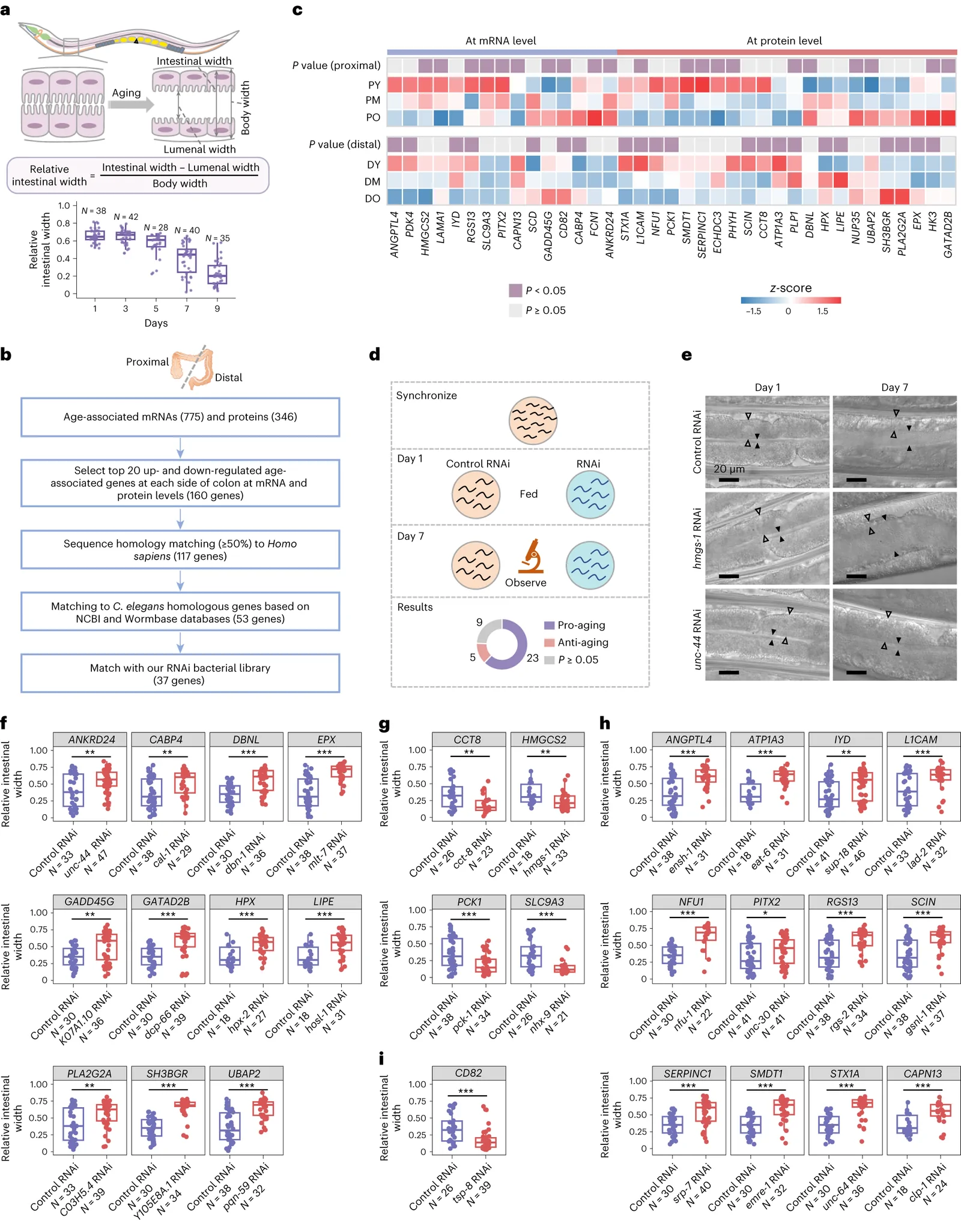
Functional validation of age-related genes in *C. elegans*. **a**, Intestinal atrophy and lumen enlargement are features of gut aging in *C. elegans*. Top, diagram of changes in *C. elegans* intestinal aging and the formula used to calculate the relative intestinal width. Bottom, the relative intestinal width of *C. elegans* decreases with age (median ± quartiles; whiskers extend to minimum and maximum values, and ‘*N*’ represents worm number). **b**, Flow chart of screening age-related genes for functional investigation. **c**, Heat map showing the expression of 37 selected genes in the proximal and distal colon. Purple indicates *P* < 0.05; gray indicates *P* ≥ 0.05 (one-way ANOVA). **d**, Workflow diagram for validating age-related genes in *C. elegans*. **e**, Representative results after knocking down the corresponding genes with RNAi. Compared to the control RNAi group, the *hmgs-1* RNAi group exhibited enlarged lumens, whereas the *unc-44* RNAi group showed the opposite results. Scale bars, 20 μm. **f**–**i**, Box plots depicting the relative intestinal width of *C. elegans* after knocking down the target gene using RNAi (median ± quartiles; whiskers extend to minimum and maximum values, and ‘*N*’ represents worm number). Notably, the analysis revealed significant increases in relative intestinal width when silencing 11 genes whose expression increased with age (**f**) and 12 genes whose expression decreased with age (**h**). Moreover, significant decreases in relative intestinal width were observed when silencing four genes whose expression decreased with age (**g**) and one gene whose expression increased with age (**i**). *P < 0.05, **P < 0.01 and ***P < 0.001, two-sided Student’s *t*-test. Raw and processed data and unprocessed images for drawing are provided in Source Data File 4 and Source Image File 1, respectively. Source data

Numerous age-associated molecules, including mRNAs, proteins, phosphosites and metabolites, have been identified in the large intestines of monkeys. However, their functions in large intestinal aging remain unknown. Prior studies in *Caenorhabditis* *elegans* revealed that intestinal aging is characterized by alterations in lumen width and intestinal atrophy^49,50^. By subtracting the luminal width from the intestinal width and dividing it by the body width^49^, we observed that the relative intestinal width gradually decreased from 0.65 on day 1 to 0.2 on day 9 during normal aging. An obvious drop of the relative intestinal width was observed from day 3 (Fig. 4a). After appropriate screening, the roles of 37 age-associated genes in gut atrophy were assessed using this model (Fig. 4b). Notably, these genes exhibited consistent age-related changes in the proximal and distal colon (Fig. 4c). To systematically assess their impact, synchronized *C. elegans* was treated with control or targeted gene RNA interference (RNAi), followed by lumen width measurement after 7 days (Fig. 4d). Among the 37 genes, 23 were found to promote gut aging, whereas five prevented it (*P* < 0.05, two-sided Student’s *t*-test) (Fig. 4d). Among the 28 effective genes, 11 pro-aging genes (*ANKRD24*, *CABP4*, *DBNL*, *EPX*, *GADD45G*, *GATAD2B*, *PLA2G2A*, *HPX*, *LIPE*, *SH3BGR* and *UBAP2*) (Fig. 4e,f) and four anti-aging genes (*CCT8*, *HMGCS2*, *PCK1* and *SLC9A3*) (Fig. 4e,g) showed increased and reduced expression, respectively, with age, indicating their involvement in large intestinal aging. Silencing *HMGCS2*, a positive regulator of ISC differentiation, led to widened lumen width (Fig. 4e,g), consistent with previous findings^21^. Additionally, 12 pro-aging genes (*ANGPTL4*, *ATP1A3*, *L1CAM*, *IYD*, *NFU1*, *SCIN*, *PITX2*, *RGS13*, *SERPINC1*, *SMDT1*, *STX1A* and *CAPN13*) decreased with age (Fig. 4h), suggesting a diminished contribution to large intestinal aging in non-human primates. Notably, one anti-aging gene, *CD82*, exhibited elevated levels with age (Fig. 4i), suggesting a potential protective role against gut atrophy during aging.

**Extended Data Fig. 6:**
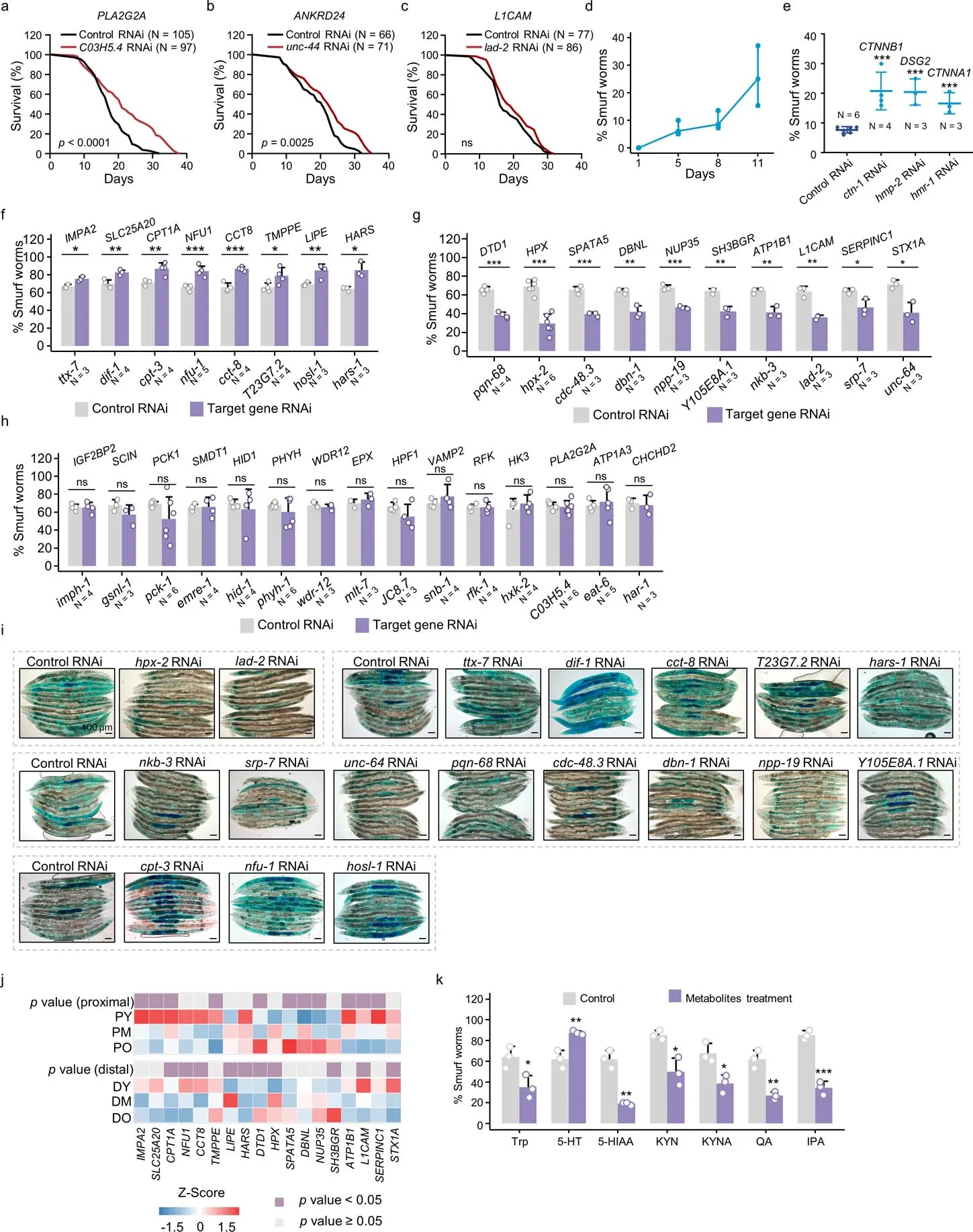
Functional validation of age-related genes in *C. elegans*. **a-c**, Effects of representative age-related gut genes on lifespan. *C03H5.4* RNAi (**a**) increases worm lifespan. Control RNAi: median survival 17 days. *C03H5.4* RNAi: median survival 21 days (*p* < 0.0001, log rank test). *unc-44* RNAi (**b**) increases worm lifespan. Control RNAi: median survival 21 days. *unc-44* RNAi: median survival 23 days (*p* = 0.0025, log rank test). *lad-2* RNAi (**c**) showed no significant effect on lifespan. **d**, Percentage of smurf phenotype in wild-type worms with age. Percentage of smurf worms was assessed at D1, D5, D8, and D11 in each experiment with ~30 worms (n = 3 independent replicates, mean ± SD). **e**, Percentage of smurf phenotype upon knocking out cell adhesion-related genes (two-tailed Student’s *t* test; mean ± SD). **f-h**, Histogram representing percentage of smurf worms observed under a DIC microscope. 8 genes showing protective effects (**f**), 10 genes showing destructive effects (**g**), and 15 genes displaying no significant effects (**h**). Two-tailed Student’s *t* test. Data are mean ± SD. **i**, Representative DIC images of control and target gene RNAi*-*treated worms soaked in blue food dye for 5 h on D8. Enclosed region by dotted lines indicates the use of same control RNAi treatment. Scale bars, 100 μm. **j**, Heatmap showing the expression of 18 effective genes across different age and locations. Purple indicates *p* < 0.05, and gray indicates *p* ≥ 0.05 (one-way ANOVA). **k**, Histogram showing percentage of smurf worms observed under a DIC microscope (n = ~30 worms) (n = 3 independent experiments, mean ± SD). In this figure, *p < 0.05, **p < 0.01, and ***p < 0.001, and ‘ns’ means no significance, two-tailed Student’s *t* test. ‘N’ represents worm number (**a-c**) and experimental repeats (**d-h**). Raw and processed data for drawing are provided in Source Data File 13. Source data

Among the 28 genes linked to intestinal atrophy, some are known to influence organismal lifespan. Notably, *ATP1A3* mutations extend the *C. elegans* lifespan^51^, and *PCK1* overexpression is correlated with increased mouse longevity^52^. We also demonstrated that silencing *PLA2G2A* and *ANKRD24* significantly extended the lifespan of *C. elegans*, whereas *L1CAM* knockdown had a negligible impact on lifespan (Extended Data Fig. 6a–c). Overall, we identified 15 genes pivotal for driving large intestinal aging in non-human primates.

## Effects of age-related genes on gut barrier integrity

**Fig. 5:**
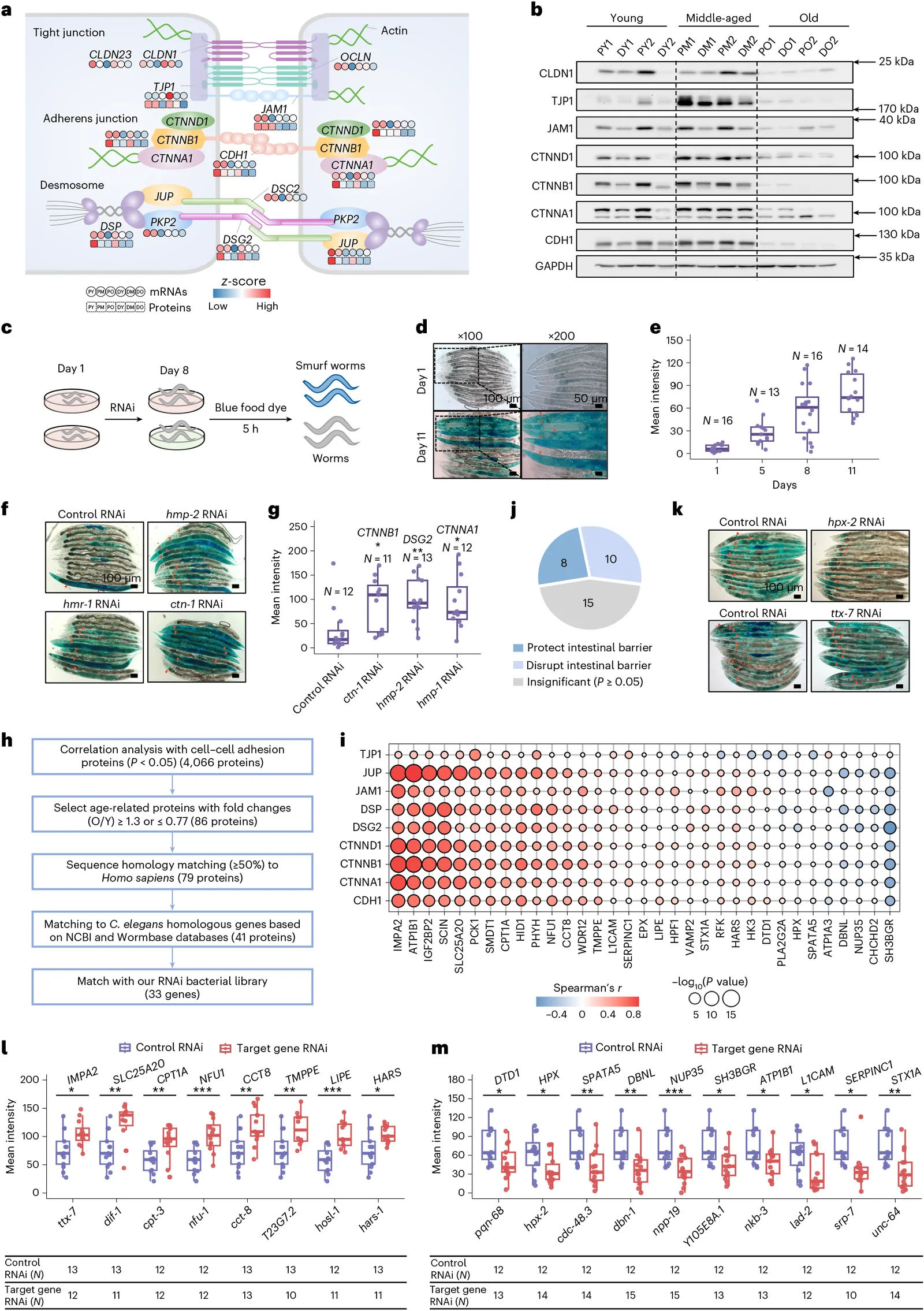
Effects of age-related genes on gut barrier integrity. **a**, Scaled protein and mRNA expression of cell–cell junction genes across three age groups in the proximal and distal colon. **b**, Representative western blotting results of cell–cell junction proteins in *M. fascicularis* large intestinal samples. **c**, Workflow of assays for evaluating gut barrier integrity in *C. elegans*. **d**, Representative DIC microscope pictures of worms showing dye leakage from the intestinal lumen into the body cavity with age. Scale bars, 100 μm (left panels) and 50 μm (right panels). **e**, Quantification of age-related Smurf phenotype in wild-type worms on D1, D5, D8 and D11 (median ± quartiles; ‘*N*’ represents worm number). **f**,**g**, Representative DIC images (**f**) and box plot (median ± quartiles; ‘*N*’ represents worm number) (**g**) showing the effects of silencing cell–cell adhesion-related genes on worm intestinal barrier function. The control and target gene RNAi-treated worms were soaked in blue food dye for 5 h on D8 of adulthood. Red arrowheads indicate worms with Smurf phenotype. Scale bars, 100 μm. *P < 0.05, **P < 0.01 and ***P < 0.001, two-sided Student’s *t*-test. **h**, Flow chart of screening age-related genes for Smurf worm assays. **i**, Bubble plot showing the correlation between 33 selected proteins and nine cell–cell adhesion proteins. **j**, Pie chart showing the overall results of the Smurf worm assays. **k**, Representative DIC images of *hpx-2* RNAi-treated, *ttx-7* RNAi-treated and control RNAi*-*treated worms soaked in blue food dye for 5 h on D8 of adulthood. Red arrowheads indicate worms with Smurf phenotype. Scale bars, 100 μm. **l**,**m**, Box plots illustrating the mean dye leakage intensity after knocking down the corresponding gene with RNAi (median ± quartiles; whiskers extend to the minimum and maximum values, and ‘*N*’ represents worm number). Eight genes showed protective effects (**l**), and ten genes showed destructive effects (**m**). *P < 0.05, **P < 0.01 and ***P < 0.001, two-sided Student’s *t*-test. Raw and processed data and unprocessed images for drawing are provided in Source Data File 5 and Source Image File 2, respectively. PY, proximal samples from young; PM, proximal samples from middle age; PO, proximal samples from old; DY, distal samples from young; DM, distal samples from middle age; DO, distal samples from old. Source data

Interestingly, a notable decline in the levels of cell–cell junction proteins, which are crucial for maintaining intestinal barrier integrity, was observed during large intestinal aging. Tight junction (TJ), adherens junctions (AJ) and desmosomal proteins exhibited higher expression in the proximal colon than in the distal colon (Fig. 5a,b). To determine the age-related genes involved in the regulation of gut barrier integrity, a *C. elegans* gut leaky model was employed^53^, in which more blue dye infiltrated the body cavity from the lumen as intestinal permeability worsened (Fig. 5c). As expected, aging promoted gut leakage (Fig. 5d,e and Extended Data Fig. 6d). Silencing genes associated with cell–cell junctions, including *CTNNA1*, *CTNNB1* and *DSG2*, validated the model by increasing blue dye infiltration (Fig. 5f,g and Extended Data Fig. 6e). Next, we investigated age-related proteins potentially implicated in regulating specific cell–cell junction proteins (Fig. 5h). Our conjecture rested on the premise that if a protein influences cell–cell adhesion, its expression might correlate with the levels of certain cell–cell junction proteins, such as TJP1, CTNND1, JUP, DSG2, CDH1, DSP, CTNNA1, JAM1 and CTNNB1. As a result, 86 proteins supported this hypothesis. We then matched human and *C. elegans* homologous genes and further performed an overlapping analysis with our RNAi bacterial library to identify 33 genes of interest (Fig. 5h,i). By individually silencing the 33 genes in *C. elegans* using RNAi (Extended Data Fig. 6f–h), we uncovered eight genes conferring significant protective effects on the gut barrier, including *IMPA2*, *SLC25A20*, *CPT1A*, *NFU1*, *CCT8*, *TMPPE*, *LIPE* and *HARS* (Fig. 5j–l and Extended Data Fig. 6f,i), alongside ten genes inducing destructive effects on the gut barrier, including *DTD1*, *HPX*, *SPATA5*, *DBNL*, *NUP35*, *SH3BGR*, *ATP1B1*, *L1CAM*, *SERPINC1* and *STX1A* (Fig. 5j,k,m and Extended Data Fig. 6g,i). Notably, among the eight protective genes, all except *LIPE* showed a significant age-related reduction in the proximal and/or distal colon (Extended Data Fig. 6f,j). Conversely, the expression of six of the ten barrier-disrupting genes—*DTD1*, *HPX*, *SPATA5*, *DBNL*, *NUP35* and *SH3BGR—*significantly increased with age in the proximal and/or distal colon (Extended Data Fig. 6g,j). These results suggest the pivotal involvement of these 14 genes in age-related gut barrier disruption.

Remarkably, nine genes—namely, *SH3BGR*, *LIPE*, *HPX*, *DBNL*, *CCT8*, *SERPINC1*, *NFU1*, *L1CAM* and *STX1A*—were found to affect both intestinal atrophy and barrier integrity. *SH3BGR*, *HPX* and *DBNL* demonstrated pro-aging and barrier-disrupting effects, with increased expression in the colon with age. *CCT8* exhibited anti-aging and protective effects on the gut barrier, and its expression decreased with age. Notably, *SERPINC1*, *L1CAM* and *STX1A* had pro-aging and barrier-disrupting effects, yet their expression decreased in the gut with age, suggesting a potential protective mechanism.

## Impacts of age-related phosphosites on gut barrier integrity

**Fig. 6:**
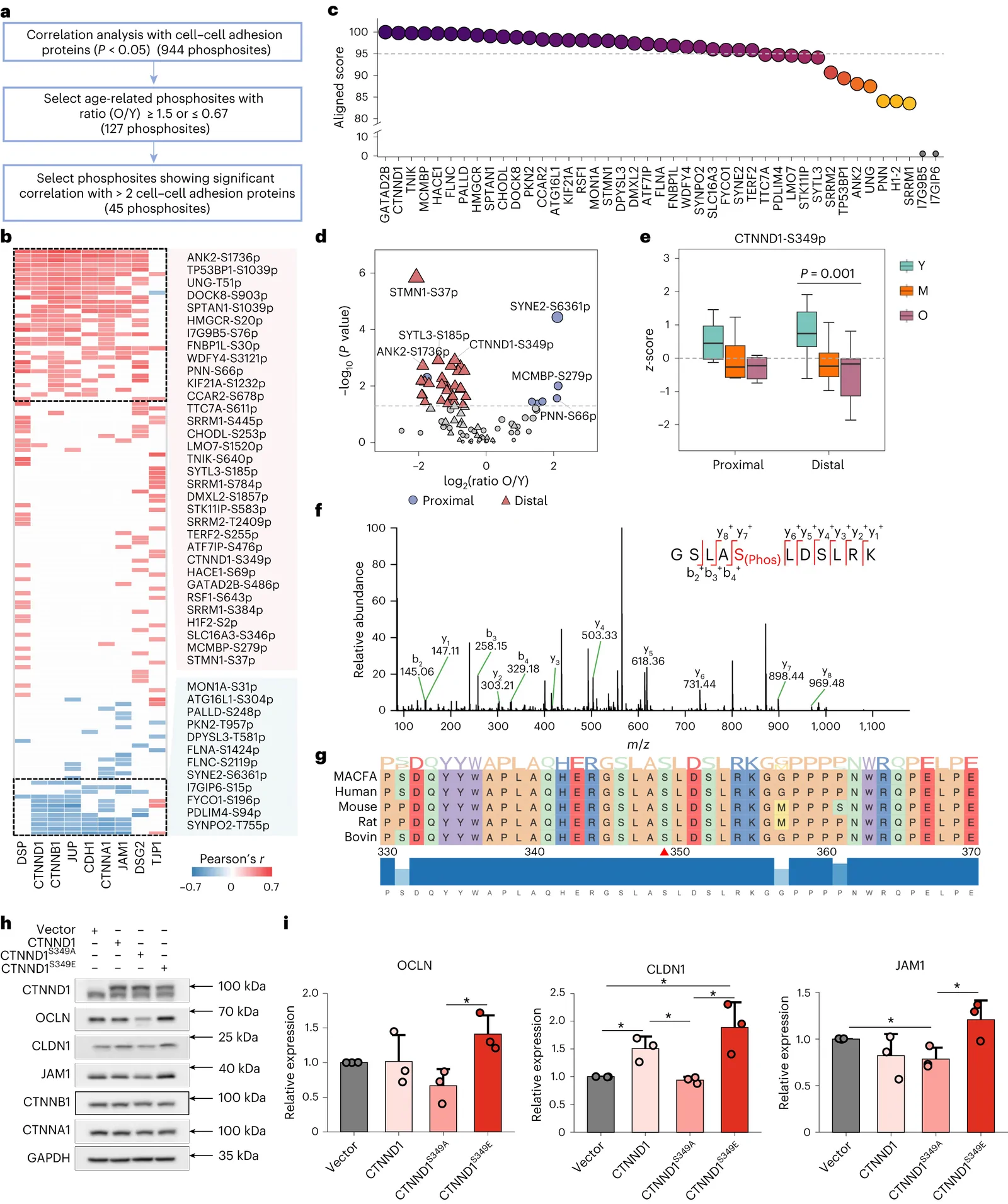
Effects of age-related phosphosites on gut barrier integrity. **a**, Flow chart of screening age-related phosphosites potentially affecting the levels of cell–cell adhesion proteins. Correlation analysis was performed between all the phosphosites and the nine cell–cell junction proteins. **b**, Heat map showing the correlation between 127 age-related phosphosites and nine cell–cell junction proteins (*P* < 0.05, Pearson’s correlation analysis). The white-filled grid in the figure represents insignificant correlations. The 45 phosphosites showing significant correlations with more than two cell–cell adhesion proteins are shown on the right of the heat map. **c**, Sequence alignment of 43 proteins corresponding to 45 phosphosites between monkeys and humans by CLUSTALW. **d**, Volcano plot displaying expression differences of 45 phosphosites in the proximal and distal colon between young and old monkeys. Circles and triangles represent phosphosites identified in the proximal and distal colon, respectively. Red and blue indicate *P* < 0.05, and gray indicates *P* ≥ 0.05 (two-sided Student’s *t*-test). **e**, Box plot displaying scaled levels of CTNND1-S349p across three age groups in the proximal (*n* = 6) and distal (*n* = 12) colon (one-way ANOVA, median ± quartiles; whiskers extend to minimum and maximum values). **f**, MS/MS spectrum of the identified phosphopeptide containing CTNND1-S349p in *M. fascicularis* samples. **g**, Alignments of the partial amino acid sequence of CTNND1 containing the S349 residue among different species. Ser349 (red arrowhead) was an evolutionarily conserved site in CTNND1 in *M. fascicularis*, humans, mice, rats and bovines. **h**, Representative Western blots showing the expression of some cell–cell adhesion proteins in Caco-2 cells stably transfected with the wild-type CTNND1, CTNND1^S349A^ or CTNND1^S349E^ plasmid. **i**, Bar graphs displaying the levels of OCLN, CLDN1 and JAM1 quantified by densitometry (*n* = 3 independent experiments, mean ± s.d.). *P < 0.05, two-sided Student’s *t*-test. Raw and processed data and unprocessed images for drawing are provided in Source Data File 6 and Source Image File 3, respectively. MACFA, *Macaca fascicularis*; Y, young; M, middle-aged; O, old. Source data

With the insights gained from pathway analysis underscoring the potential role of phosphorylation in cell–cell adhesion regulation, our next step was to pinpoint specific phosphosites that might impact intestinal barrier integrity. Building on a similar hypothesis to identify cell–cell adhesion-associated proteins (Fig. 5h), we obtained 944 phosphosites that exhibited a significant correlation with at least one of the nine cell–cell junction proteins (Pearson’s correlation analysis, *P* < 0.05) (Fig. 6a). Applying a fold change threshold of 1.5 or higher between young and old individuals led to the identification of 127 sites (Fig. 6a). Among these sites, 45 phosphosites demonstrated a significant correlation with more than two cell adhesion-related proteins, as depicted on the right side of the heat map (Fig. 6b). Notably, all 45 phosphosites were fully conserved in both humans and monkeys. Additionally, 29 out of the 43 phosphoproteins shared over 95% identity between these two species (Fig. 6c). Among the 45 phosphosites, phosphorylation of CTNND1 at S349 (CTNND1-S349p) exhibited an age-related decrease in both colon sections, albeit more prominently in the distal colon (Fig. 6d–g). Introducing a mutation of S349 to E (S349E) to mimic the effect of phosphorylation significantly increased the expression of cell–cell junction proteins, such as OCLN, CLDN1 and JAM1 (Fig. 6h,i), underscoring the contribution of age-associated CTNND1-S349p dysregulation to barrier integrity disruption.

## Effects of Trp metabolites on gut homeostasis

**Fig. 7:**
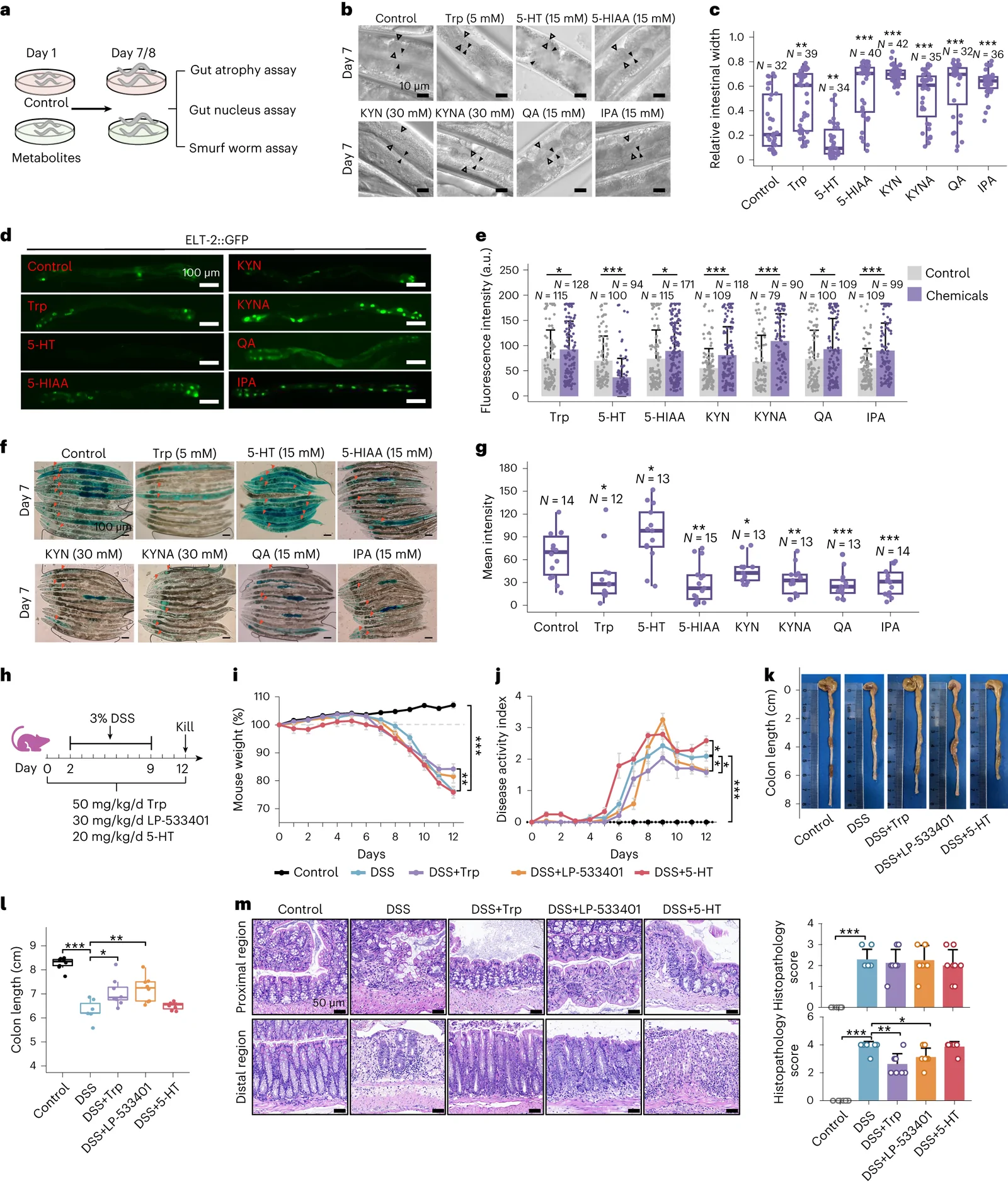
Effects of Trp metabolites on gut homeostasis. **a**, Workflow diagram for exploring Trp metabolite functions. **b**, Representative DIC microscopy images showing intestinal atrophy on D7. Scale bars, 10 μm. **c**, Box plot illustrating statistical analysis of relative intestinal width on D7 (two-sided Student’s *t*-test, median ± quartiles; whiskers extend to minimum and maximum values, and ‘*N*’ represents worm number). **P < 0.01 and ***P < 0.001. **d**, Representative micrographs of *elt-2*::gfp worms on D7. Scale bars, 100 μm. **e**, Bar graph showing average GFP intensity (two-sided Student’s *t*-test, mean ± s.d.; ‘*N*’ represents worm number). *P < 0.05 and ***P < 0.001. **f**, Representative DIC images of worms soaked in blue food dye for 5 h on D8. Red arrowheads indicate Smurf phenotype. Scale bars, 100 μm. **g**, Box plot representing the mean dye leakage intensity (two-sided Student’s *t*-test, median ± quartiles; whiskers extend to minimum and maximum values, and ‘*N*’ represents worm number). *P < 0.05, **P < 0.01 and ***P < 0.001. **h**, Experimental procedures. **i**, Daily mouse body weight (two-sided Student’s *t*-test, mean ± s.e.m.). **P < 0.01 and ***P < 0.001. **j**, Disease activity index determined by weight loss, stool consistency and bleeding (two-sided Student’s *t*-test, mean ± s.e.m.; whiskers extend to minimum and maximum values). *P < 0.05 and ***P < 0.001. **k**, Representative pictures of mouse colon length. **l**, Statistical analysis of colon length (two-sided Student’s *t*-test, mean ± quartiles; whiskers extend to minimum and maximum values). *P < 0.05, **P < 0.01 and ***P < 0.001. **m**, H&E images of the ascending and descending colons. Scale bars, 50 μm. Box plot illustrating statistical results of histopathology score (0 = none; 1 = very mild; 2 = mild; 3 = moderate; 4 = severe) (two-sided Student’s *t*-test, mean ± s.d.). *P < 0.05, **P < 0.01 and ***P < 0.001. In Fig. 7i–m, each group included eight mice, except for the DSS group that contained seven mice. Raw and processed data and unprocessed images for drawing are provided in Source Data File 7 and Source Image File 4, respectively. MAOB, monoamine oxidase B; MAOA, monoamine oxidase A; AFMID, arylformamidase; KMO, kynurenine 3-monooxygenase; HAAO, 3-hydroxyanthranilate 3,4-dioxygenase; QPRT, quinolinate phosphoribosyltransferase; NAD^+^, nicotinamide adenine dinucleotide. Source data

Given the distinct preference of Trp metabolism pathways in the proximal and distal colon, we evaluated the effects of Trp and its metabolites, including 5-HT, 5-hydroxyindoleacetic acid (5-HIAA), KYN, quinolinic acid (QA), kynurenic acid (KYNA) and microbiota-derived 3-indolepropionic acid (IPA), on intestinal morphology, intestinal nucleus and barrier integrity in *C. elegans* during aging (Fig. 7a). Treatment with these compounds demonstrated protective effects against intestinal atrophy (Fig. 7b,c), with notable improvements in intestinal nuclei fluorescence intensity^50^, except for 5-HT, which exhibited the opposite effect (Fig. 7d,e). Additionally, only 5-HT treatment increased gut leakage in a leaky gut model (Fig. 7f,g and Extended Data Fig. 6k), accompanied by smaller worm sizes (Fig. 7f), likely due to the pro-inflammatory effects of 5-HT^54^.

5-HT plays a key role in the pathogenesis of colitis^55^. Considering the heterogeneity of large intestinal aging, it is conceivable that 5-HT could impact left-sided and right-sided colitis differently. To verify this hypothesis, a dextran sulfate sodium (DSS)-induced colitis model using male mice was applied (Fig. 7h). The study included untreated mice and those treated with DSS, DSS+Trp, DSS+5-HT or DSS+Tph1 inhibitor (LP-533401) (Fig. 7h). Tph1 is a key enzyme in the 5-HT biosynthesis pathway, and inhibition of Tph1 can reduce the production of 5-HT. The findings showed that 5-HT aggravated colitis, whereas inhibiting 5-HT biosynthesis or Trp treatment alleviated this effect (Fig. 7i–l). H&E staining of the proximal and distal colon showed that 5-HT or LP-533401 treatment had little effect on right-sided colitis. In contrast, left-sided colitis severity increased with 5-HT treatment but was alleviated by suppressing 5-HT production using LP-533401 (Fig. 7m). This finding emphasizes the heterogeneity of the large intestine, with left-sided colitis being more responsive to 5-HT fluctuations.

## HPX potentially links gut aging to CRC progression

**Extended Data Fig. 7:**
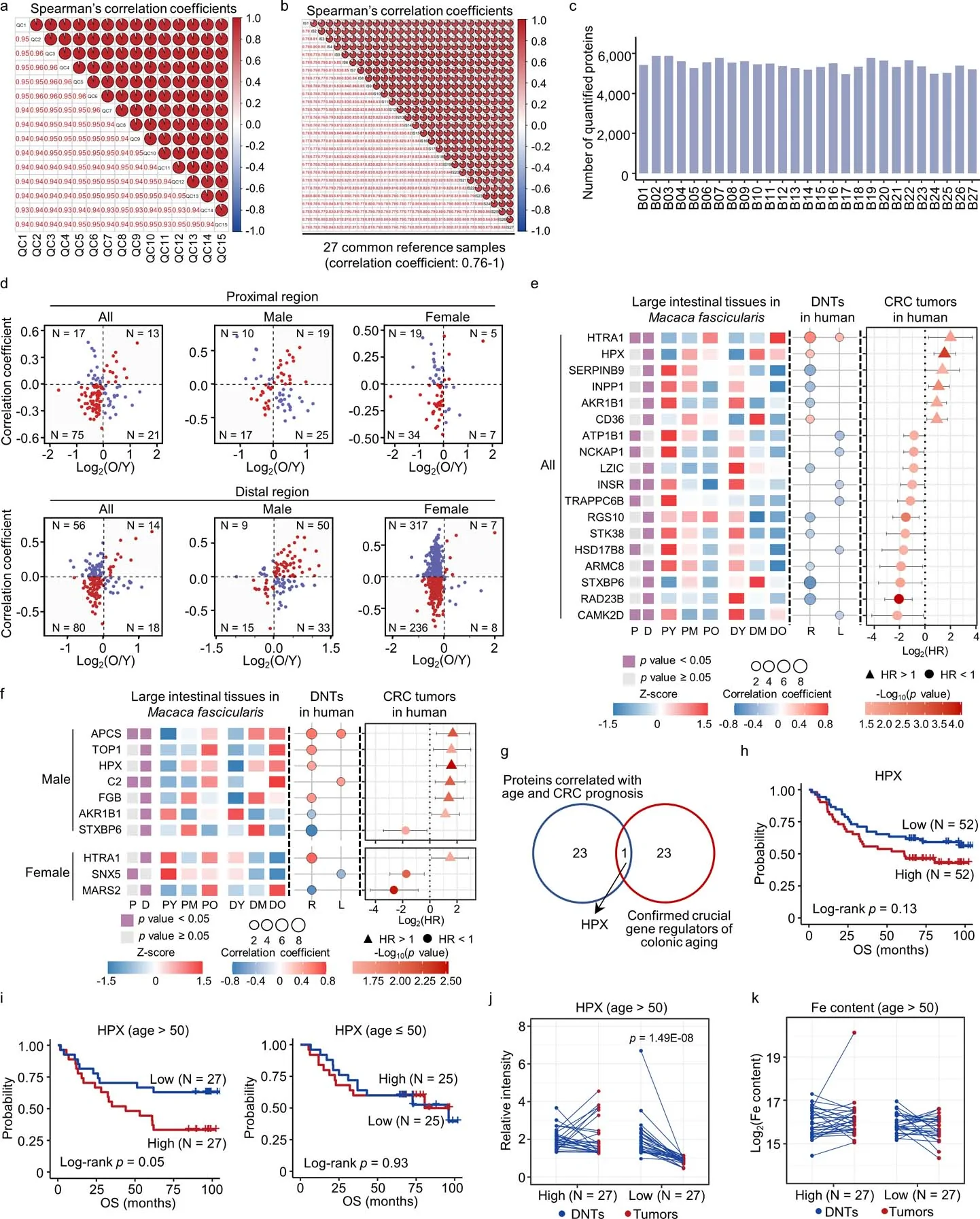
Proteins potentially linking large intestinal aging to CRC progression. **a,b**, Spearman’s correlation analysis of QCs (**a**) and common reference samples (**b**) of each batch to assess the stability of proteomics (*p* < 0.05). **c**, Histogram showing identified protein number in each batch. **d**, Scatterplots showing correlation difference of age-associated proteins in the large intestinal tissues of *Macaca fascicularis* and in the DNTs of humans, with considering sex effects. **e,f**, Combined charts illustrating age-associated proteins in the proximal and distal colon of *Macaca fascicularis* and DNTs of humans that predict the prognosis of CRC patients (**e**), with considering sex effects (**f**). Purple indicates *p* < 0.05, and gray indicates *p* ≥ 0.05 in left panel (one-way ANOVA); *p* value for the cox proportional hazards regression is presented in the right panel (*p* < 0.05). **g**, Venn diagram highlighting confirmed crucial regulators showing a correlation with age in both *Macaca fascicularis* and humans and exhibiting a correlation with CRC prognosis. **h,i**, Kaplan-Meier plots showing survival outcomes of all CRC patients (**h**), CRC patients over 50 years old and CRC patients younger than 50 years (n = 52 sample per groups, *p* = 0.13, log rank test) (**i**) based on HPX expression (divided into two groups according to the median expression, n = 52 sample per groups, *p* = 0.05 (age > 50), *p* = 0.93 (age ≤ 50), log rank test). **j**, Comparison of HPX expression in DNTs between CRC patients with high and low HPX expression in tumors. **k**, Comparison of relative iron content in DNTs and tumors between CRC patients with high and low HPX expression in tumors (n = 27 sample per groups, *p* = 1.49e-8, Wilcoxon signed-rank test). Raw and processed data for drawing are provided in Source Data File 14. Source data

Aging-associated epigenetic changes and the aging microenvironment play pivotal roles in cancer development^56,57^. The aging microenvironment, acting as a double-edged sword, can either inhibit or promote tumor development. On the one hand, the senescence-associated secretory phenotype (SASP) can promote the elimination of senescent cells by the immune system and contribute to tissue repair^58^. On the other hand, the accumulation of SASP factors may lead to chronic inflammation, creating an environment conducive to cancer progression^56^. Proteomic analysis of 104 paired CRC tissues revealed that protein changes with age in right-sided distant normal tissues (DNTs) were consistent with those in the proximal colon of monkeys (Extended Data Fig. 7a–c). However, this trend was absent in left-sided DNTs, indicating potential susceptibility to influences from distal colon tumors (Extended Data Fig. 7d). Further investigation of the influence of sex on these correlations revealed that proximal tumors in males exerted a greater influence on corresponding DNTs, whereas distal tumors in females had a more pronounced impact on corresponding DNTs (Extended Data Fig. 7d). Subsequently, we proceeded to identify the proteins showing consistent age-related changes in human DNTs and monkey intestines (Extended Data Fig. 7e,f). Overall survival (OS) analysis of patients with CRC stratified by tumor protein levels identified 24 proteins (Extended Data Fig. 7e,f), with HPX standing out (Extended Data Fig. 7g). Intriguingly, HPX, a confirmed regulator of large intestinal aging and gut barrier integrity, emerged as a predictor of prognosis in patients with CRC over 50 years of age but not in patients younger than 50 years of age (Extended Data Fig. 7h,i), indicating that an age-associated increase in HPX might contribute to cancer progression. Although HPX is a circulating protein, its level in DNTs of certain patients with CRC was not as low as those in corresponding tumors (Extended Data Fig. 7j). Previous research has proposed that hemoglobin entering cells without the protection of HPX could induce ferroptosis^59^. Examination of the iron content in HPX-low tumors revealed a close match to that in HPX-high tumors (Extended Data Fig. 7k). This led to the speculation that low levels of HPX might allow more iron to directly enter cancer cells, potentially causing ferroptosis and resulting in a favorable prognosis for patients with CRC^60^. Overall, we identified age-associated proteins in the large intestine that are potentially linked to CRC.

## Discussion

In summary, we performed location-resolved and sex-resolved omics characterization of large intestinal aging in non-human primates and validated the functions of many age-associated molecules. This work yielded several key findings. First, we unveiled the heterogeneous and asynchronous molecular aging patterns between the proximal and distal colon that are influenced by sex. Second, 32 essential regulators crucial for intestinal aging, including 24 genes, one phosphosite and seven metabolites, were identified (Supplementary Table 1). Third, we found a more active 5-HT pathway in the distal region and a greater preference for the KYN pathway in the proximal region. Moreover, distal colitis was more sensitive to 5-HT fluctuations. Finally, we identified 24 age-associated proteins in the large intestine potentially contributing to the progression of CRC.

Large intestine heterogeneity extends to various aspects, such as morphology, physiological functions, blood supply and microenvironment. For example, from the proximal to the distal colon, the thickness of the mucus increases continuously^61^, and B cells in the colon show a gradient of activation^62^. In addition, the expression of cell cycle genes and apoptosis markers is higher in the distal colon^63^. This work addressed the heterogeneity of large intestinal aging^21^, emphasizing inconsistent or even opposing changes in vital regulators between the proximal and distal colon, suggesting distinct contributions of gut aging regulators in different locations.

The actin cytoskeleton, crucial for epithelial junction remodeling and integrity, is regulated by actin-binding proteins (ABPs)^64^ and phosphorylation^65^. For example, phosphorylation of TJ, AJ and desmosomal proteins^66–69^ and phosphorylation of ABPs, such as cofilin (S3p)^70^, fimbrin (T103p)^71^ and drebrin (S647p)^72^, can modulate apical junction complex assembly/disassembly. Intriguingly, we observed continuously decreasing changes in phosphorylation associated with actin filament-based processes, signaling by Rho GTPases and cell–cell junction assembly with age in the distal colon (Fig. 2f), suggesting that changes in phosphorylation may contribute more to cell–cell junction protein homeostasis in the distal colon. The mechanisms of phosphorylation dysregulation in cell–cell adhesion remain elusive and may be driven by age-related influences, such as shifts in cell-to-cell communication^73^, variations in kinase activities and other modulators, such as magnesium ions^74^.

Large intestinal aging contributes to organismal inflammaging, which is featured by disrupted gut barrier function, bacterial entry into the bloodstream and chronic inflammation^75^. Dysbiosis and intestinal aging can disrupt the production of beneficial metabolites, including short-chain fatty acids, potentially promoting inflammation^76^. Overlap analysis of our age-related genes with inflammation-related genes from the Molecular Signatures Database (https://www.gsea-msigdb.org/gsea/msigdb) revealed 29 genes. Among them, *MYC*, *NFKB1*, *CDKN1A* and *PSEN1* impact longevity (https://genomics.senescence.info/genes/index.html). Notably, *NFKB1*, whose levels decrease with age in the distal colon of females, regulates the lifespan of mice^77^. Signaling pathways linked to inflammaging, such as mitochondrial respiration (Fig. 1e) and the MAPK signaling pathway^78,79^, also show age-related changes (Extended Data Fig. 2e). Furthermore, we identified 25 inflammaging-related metabolites (Supplementary Table 2), two of which were pro-inflammaging and 23 of which were anti-inflammaging. Among these metabolites, asymmetric dimethylarginine, a pro-inflammaging metabolite, increases with age in the distal colon of females, and six of the 23 anti-inflammaging metabolites, including allantoin^80^, fumarate^81^, pyruvate^82^, sulfoacetate^83^, triethylamine^84^ and KYNA^85^, decrease with age, potentially collectively contributing to heightened inflammation as individuals age.

The distal colon appears more susceptible to diseases or interventions. UC, a main form of chronic colitis, tends to occur in the distal colon and extends proximally^86^. The DSS-induced colitis mouse model mimics UC, showing more severe distal colitis^87^, and treatment with Trp metabolites had a profound influence on distal colitis (Fig. 7m). Moreover, previous studies indicated that tumors may influence adjacent normal tissues^88^. Intriguingly, we found that distal colon cancer may have a greater impact on adjacent normal colon tissues, extending even to tissues 10–15 cm away. Hence, caution is necessary when using DNTs as controls in tumor studies.

Although our study provides a multi-dimensional molecular landscape of large intestinal aging and identifies 32 confirmed crucial regulators, certain limitations exist. The number of samples for the analysis of age-related molecules, as well as cell–cell junction protein-associated proteins and phosphosites, is limited. Additionally, the specific cell types in the gut where these regulators exert their functions remain unclear. Further study is needed to elucidate the precise mechanisms by which these molecules contribute to large intestinal aging and CRC development.

## Methods

### Ethics statements

This study was conducted in accordance with the Ethical Treatment Guides of Non-Human Primates and was approved by the Institutional Animal Care and Use Committee of Yuanxi Biotech in Guangzhou, China (YXSW-2016-01). The collection and use of human CRC samples were approved by the Ethics Committee of Biology Research, West China Hospital at Sichuan University (2020(374)), and written informed consent was obtained from all participants or their families. We followed STROBE guidelines in the use of patient samples. The use and care of the mice were approved by the Animal Experiment Ethics Committee of the State Key Laboratory of Biotherapy at Sichuan University (20220531045).

### Experimental model and biological sample collection

*M. fascicularis* animals were housed in a standardized laboratory environment maintained at a temperature of approximately 25 °C and a 12-h light and 12-h dark cycle in Guangzhou, China. Before the experiment, all animals had no clinical or experimental history that might affect physiological aging or increased susceptibility to diseases. Large intestinal tissues were obtained from nine young (3–4 years old, five males and four females), eight middle-aged (9–10 years old, four males and four females) and nine old (15–16 years old, five males and four females) *M. fascicularis* animals and snap frozen in liquid nitrogen. C57BL/6 mice (8 weeks old) were obtained from GemPharmatech and housed in a standard specific pathogen-free laboratory environment. The mice were kept in a controlled environment with a 12-h light/dark cycle, maintaining the ambient temperature at 22 ± 2 °C and humidity at 50 ± 5%. The animals had access to standard rodent chow and water at all times. Housing conditions remained uniform across all experimental and control groups. The mice were housed together for 7 days before use and fed a standard chow diet and had free access to drinking water throughout the study. Tumors and paired DNTs (10–15 cm away from the cancer tissues) from a cohort of 104 patients with CRC were collected at West China Hospital and snap frozen in liquid nitrogen.

### RNA extraction and RNA sequencing

Total RNA was extracted from fresh frozen *M. fascicularis* large intestinal tissues using TRIzol. Library preparations were sequenced on the Illumina NovaSeq 6000 PE150 platform, and 125-bp/150-bp paired-end reads were generated. The mRNA sequencing reads per sample were mapped to the *M. fascicularis* reference genome mf5 and RefSeq gene annotations by STAR (version 2.7.5a)^89^ using the two-pass model. The mf5 sequence and RefSeq gene annotation were downloaded from the UCSC Table Browser (https://genome.ucsc.edu/). The overall mapping rate per sample was more than 70%. featureCounts (Subread version 2.0.1)^90^ was used to quantify gene expression levels. The TPM was calculated from the gene read counts using R (version 4.0.2). The mRNA data in downstream analyses were filtered for genes with an average TPM > 1. The subsequent differential expression analysis was performed using the log_2_(TPM + 1) method.

### Sample preparation and TMT labeling

Collected tissues were lysed with RIPA buffer (50 mM Tris (pH 7.5), 150 mM NaCl, 0.5% sodium deoxycholate, 1% NP40, 25 mM nicotinamide, 10 mM sodium butyrate, 1% protease inhibitor cocktail, 1% phosphatase inhibitor A and 1% phosphatase inhibitor B) and homogenized with a gentleMACS Dissociator. The crude lysates were then sonicated on ice for 5 min (30% power, 3 s on and 10 s off) and centrifuged at 20,000*g* for 30 min at 4 °C. The supernatants were transferred to a new tube, and the protein concentrations were determined by Bradford assay (Bio-Rad, 5000205). The extracted proteins (50 μg) from each sample were reduced with 10 mM TCEP at 56 °C for 1 h and alkylated with 20 mM iodoacetamide at room temperature for 30 min in the dark. Protein samples were precipitated by the chloroform/methanol/water method and digested with trypsin (trypsin:protein = 1 μg:50 μg) at 37 °C for 16 h.

A TMT-based strategy was used for proteomic analyses of *M. fascicularis* large intestinal tissues and CRC tissues. In each batch of TMT-labeled samples, a common reference sample was used. For the proteomics of the *M. fascicularis* large intestinal tissues, the reference sample was labeled with TMT-126. The samples from young, middle-aged and old groups were labeled with the remaining nine channels of TMT-10plex reagents (Thermo Fisher Scientific) (127N, 127C and 128N for young; 128C, 129N and 129C for middle-aged; 130N, 130C and 131 for old). For CRC proteomics, the reference sample was labeled with 12C. Four pairs of tumorous tissues and DNTs were labeled with the other eight channels (tumors labeled with 126, 127N, 127C and 128N; DNTs labeled with 129C, 130N, 130C and 131). The labeling procedure was performed according to the instructions of the TMT kit. Specifically, TMT reagents were dissolved in anhydrous acetonitrile (ACN) and then added to the digested peptides. After incubation at room temperature for 1 h, the reaction was quenched by adding 5% hydroxylamine. The labeled peptides were combined and dried by a SpeedVac. After being redissolved in 0.1% trifluoroacetic acid (TFA), the labeled peptides were desalted with C18 SPE columns (100 mg, Waters) and fractionated via high-performance liquid chromatography (HPLC).

### HPLC fractionation

To increase the depth of protein identification, TMT-labeled peptides were fractioned by using HPLC (Shimadzu LC-2030 Plus) on a 25-cm reversed-phase column (4 μm, 4.6 × 250 mm, Poroshell, Agilent) under basic pH conditions (pH 10). The parameters were set as follows: flow rate, 1 ml min^−1^; column temperature, 40 °C; and ultraviolet detection at 214 nm. Gradient elution was performed on a mixture of buffer A (98% H_2_O and 2% ACN, 10 mM ammonium formate, pH 10) and buffer B (90% ACN, 10% H_2_O, 10 mM ammonium formate, pH 10). The 120-min LC gradient was set as follows: 3–35% B in 90 min; 35–60% B in 15 min; 60–100% B in 10 min; and 100–3% B in 5 min. The 120 fractions of each batch of *M. fascicularis* samples were combined into 24 fractions, and the 120 fractions of each batch of CRC samples were combined into 15 fractions. The combined fractions were dried and desalted with C18 ZipTip before liquid chromatography with tandem mass spectrometry (LC–MS/MS) analysis.

### Enrichment of phosphopeptides by Fe-NTA agarose

One milligram of tryptically digested peptides was divided into nine fractions by using C18 SPE columns (100 mg, Waters) and then combined into three fractions. After being dried by vacuum, the three fractions were reconstituted with 300 μl of loading buffer (85% ACN, 0.1% TFA). The enrichment of phosphorylated peptides was carried out with PureCube Fe-NTA agarose (Cube Biotech, 31403). Samples dissolved in loading buffer were incubated with Fe-NTA agarose at room temperature for 1 h. Subsequently, the agarose beads were washed four times with washing buffer (80% ACN, 0.1% TFA) and then washed with 150 μl of elution buffer (40% ACN, 15% ammonium hydroxide). The elution buffer was then neutralized by using 7 μl of 20% TFA. After being dried by vacuum, the samples were desalted with C18 ZipTip and subjected to LC–MS/MS analysis.

### MS analysis

The desalted peptides were loaded onto a 75 μm (inner diameter) × 2.5 cm (length) trap column (Spursil C18 5-μm particle size, Dikma) and analyzed on a 75 μm (inner diameter) × 25 cm (length) analytical column (Reprosil-Pur C18-AQ 1.9-μm particle size, Dr. Maisch).

For proteomic analysis of *M. fascicularis* large intestinal tissues, LC–MS/MS analysis was carried out using an EASY-nLC 1000 nanoflow LC instrument coupled with a Q Exactive Plus Quadrupole-Orbitrap mass spectrometer (Thermo Fisher Scientific). Peptides were analyzed using a 65-min gradient of 6% to 95% buffer B (0.1% formic acid (FA) in 95% ACN) at a flow rate of 330 nl min^−1^. Data-dependent acquisition (DDA) was performed in positive ion mode. Full MS scans (*m*/*z* 350–1,600) were acquired with a resolution of 70,000. The automatic gain control (AGC) value was set to 3 × 10^6^, and the maximum injection time (MIT) was 20 ms. For MS/MS analysis, the top 15 most intense precursor ions were selected at an isolation window of 0.6 *m*/*z* and then fragmented with a normalized collision energy of 30%. The AGC value of MS/MS was set to 1 × 10^5^, and the MIT was set to 100 ms. Precursor ions with charge states of *z* = 1 and *z* = 8 or an unassigned charge state were excluded. The dynamic exclusion duration was 50 s.

For phosphoproteomic analysis of *M. fascicularis* large intestinal tissues, LC–MS/MS analysis was carried out using an EASY-nLC 1200 nanoflow LC instrument coupled with a Q Exactive HF-X Quadrupole-Orbitrap mass spectrometer (Thermo Fisher Scientific). Peptides were analyzed using a 65-min gradient of 14% to 95% buffer B (0.1% FA in 95% ACN) at a flow rate of 330 nl min^−1^. DDA was performed in positive ion mode. Full MS scans (*m*/*z* 350–1,800) were acquired with a resolution of 60,000. The AGC value was set to 3 × 10^6^, and the MIT was set to 20 ms. For MS/MS analysis, the top 20 most intense precursor ions were selected at an isolation window of 1.6 *m*/*z* and then fragmented with stepped normalized collision energies of 25% and 27%. The AGC value of MS/MS was set to 1 × 10^6^, and the MIT was set to 64 ms. Precursor ions with charge states of *z* = 1 and *z* = 8 or an unassigned charge state were excluded. The dynamic exclusion duration was 50 s. Continuous QC samples were analyzed every 21 MS injections.

For proteomic analysis of CRC tissues, LC–MS/MS analysis was carried out using an EASY-nLC 1200 nanoflow LC instrument coupled with an Orbitrap Exploris 480 mass spectrometer (Thermo Fisher Scientific). Peptides were analyzed using a 65-min gradient of 4% to 100% buffer B (0.1% FA in 80% ACN) at a flow rate of 300 nl min^−1^. DDA was performed in positive ion mode. Full MS scans (*m*/*z* 350–1,800) were acquired with a resolution of 60,000. The normalized AGC value was set to 300%, and the MIT was 50 ms. For MS/MS analysis, the top 20 most intense precursor ions were selected at an isolation window of 0.7 *m*/*z* and then fragmented with a normalized collision energy of 36%. The normalized AGC value for MS/MS was set to 75%, and the MIT was set to 80 ms. Precursor ions with charge states of *z* = 1 and *z* = 8 or an unassigned charge state were excluded. The dynamic exclusion duration was set to 50 s. Continuous QC samples were analyzed every 30 MS injections.

### MS database searching

The raw files for proteomics and phosphoproteomics were searched using MaxQuant (version 1.6). Proteomic data from *M. fascicularis* large intestinal tissues were searched against the *M. fascicularis* protein sequence database (30,202 protein sequences), and CRC proteomic data were searched against the Swiss-Prot human protein sequence database (updated in April 2019; 20,431 sequences). The precursor peptide mass tolerance was set to 10 ppm, and the fragment ion mass tolerance was set to 0.02 Da. The minimum amino acid length was set to 6. Carbamidomethylation of cysteine was assigned as a fixed modification. Oxidation of methionine and protein N-terminal acetylation were assigned as variable modifications. Two missed trypsin cleavages were allowed. Peptides with a false discovery rate < 1% were kept for further data processing. For phosphoproteomics, phosphorylated serine, threonine and tyrosine were also added to the above variable modifications. Phosphoproteomic quantitative analysis was performed by label-free quantification.

### Proteomic data analysis

For proteomics, the unique peptides were extracted, and the intensities of different unique peptides of the same protein were summed. Proteins with ≥2 unique peptides were retained. We first normalized the protein intensities. The intensity of each protein was divided by the corresponding coefficient, which was obtained by dividing the total protein intensity of each sample by the average total protein intensity of all samples. The protein intensity was subsequently divided by the protein intensity of the reference sample in the same batch. The relative protein intensities of all the samples in the different batches were combined. The proteins detected in ≥50% of the samples in each subgroup (PY, PM, PO, DY, DM and DO) were used for subsequent analysis.

### Phosphoproteomic data analysis

For phosphoproteomics, to normalize the phosphopeptide intensities, the intensity of each phosphopeptide was divided by the corresponding coefficient, which was obtained by dividing the total phosphopeptide intensity of each sample by the average total intensity of phosphopeptides in all the samples. After subsequent log_2_ transformation and centering, phosphopeptides detected in ≥50% of the samples were kept. Missing values were filled out using the random forests method of the ‘mice’ package^91^. After filling in missing values, the phosphoproteomic data were used for subsequent analysis.

### Untargeted metabolomic analysis

The tissues were weighed, and hydrophilic metabolites were extracted from *M. fascicularis* large intestinal tissues with pre-cooled 80% (v/v) HPLC-grade methanol containing two isotopic amino acids (1 μg ml^−1^ ^13^C_6_-Lys and ^13^C_6_^15^N_4_-Arg)^92^ by adding 1 ml of 80% (v/v) methanol to 100 mg of tissue. The tissues were homogenized using the protein_01_01 program with a gentleMACS Dissociator for 15 s, with a stop of 30 s. This process was repeated three times. Then, the whole lysate was transferred to an Eppendorf tube, after which the metabolites were fully extracted with methanol at 4 °C for 1 h. The lysate was centrifuged at 14,000*g* (4 °C) for 20 min, and the same volume of supernatant of each sample was transferred to a new Eppendorf tube. In addition, 20 μl from each sample was mixed, which was used as a QC mixture for MS detection. The supernatants of all the samples were concentrated and dried in vacuo.

An UltiMate 3000 UHPLC (Dionex) coupled with Q Exactive mass spectrometry (Thermo Fisher Scientific) was used to analyze the hydrophilic metabolites extracted from *M. fascicularis* large intestinal tissues. The column temperature was 35 °C. In positive ion mode, gradient elution was performed using mobile phase A (95% ACN, 10 μM ammonium formate, 0.0001% formic acid) and mobile phase B (50% ACN, 10 μM ammonium formate, 0.0001% formic acid) on an Atlantis HILIC silica column (2.1 × 100 mm, Waters). The linear gradient was as follows: 0 min, 1% B; 2 min, 1% B; 3.5 min, 20% B; 17 min, 80% B; 17.5 min, 99% B; 19 min, 99% B; 19.1 min, 1% B; 22 min, 1% B. In negative ion mode, mobile phase A (95% ACN, 10 μM ammonium acetate, pH 9) and mobile phase B (50% ACN, 10 μM ammonium acetate, pH 9) were eluted by a gradient according to proportional mixing on a BEH Amide column (2.1 × 100 mm, Waters). The linear gradient was as follows: 0 min, 5% B; 2 min, 5% B; 4 min, 20% B; 18 min, 85% B; 19 min, 95% B; 21 min, 95% B; 21.1 min, 5% B; 25 min, 5% B. Analysis of polar metabolites was performed by a Q Exactive Orbitrap mass spectrometer (Thermo Fisher Scientific). The spray voltage was 3.5 kV for positive ion mode and 2.5 kV for negative ion mode. The capillary temperature was 275 °C for positive ion mode and 320 °C for negative ion mode. The mass range (*m*/*z*) was 70–1,050 for positive ion mode and 80–1,200 for negative ion mode^93^.

The raw files of untargeted metabolomics were searched by using TraceFinder (Thermo Fisher Scientific) based on a homemade metabolite database. The mass tolerance of the precursor ions was 10 ppm, and the mass tolerance of the fragment ions was 15 ppm. For quantitation, a retention time shift of 0.25 min was allowed. Metabolites with missing values in ≥20% of the samples or with CV values ≥ 0.3 among the QC replicates were removed. To normalize the intensities of the metabolites, the intensity of each metabolite was divided by the corresponding correction factor of that sample (correction factor = total intensity per sample / average total intensity of all samples). Missing values were filled with half of the minimum value in the samples.

### Targeted analysis of 3-hydroxybutyrate

The tissues were weighed (approximately 10 mg per sample), and 200 μl of pre-cooled 80% (v/v) methanol was added. The tissues were homogenized with Tissue Prep (Bio-Xplorer) at 1,500*g* for 10 s, with a stop of 1 min. This process was repeated three times. Then, an additional 800 μl of 80% spiked methanol with isotopic chemicals (120.89 μM ^13^C_6_-D-glucose, 23.12 mM ^13^C_5_-L-glutamate-^15^N) was added. The Eppendorf tube was kept at −80 °C for 30 min to fully extract the metabolites. Then, the lysate was sonicated in an ice bath for 10 min and centrifuged at 16,200*g* (4 °C) for 20 min. Then, 600 μl of the supernatant was transferred to a new Eppendorf tube. Next, 500 μl of 80% methanol was added to Eppendorf tubes with pellets, which were vortexed for 5 min (4 °C). Subsequently, the lysate was centrifuged at 16,200*g* for 30 min at 4 °C, after which the supernatants from the same sample were pooled into one tube. Finally, 20 μl of each sample was taken from the tube and mixed, which was used as a QC mixture for MS detection. The remaining extracts were concentrated, dried in vacuo and stored at −80 °C.

A SCIEX ExionLC UHPLC liquid chromatograph coupled with SCIEX Triple Quad 6500 + LC–MS/MS was used to analyze the metabolites. An ACQUITY UPLC BEH Amide column was used (100 × 2.1 mm, 1.7 μm) (Waters), and the column temperature was set at 40 °C. Gradient elution was performed at a flow rate of 0.3 ml min^−1^ with mobile phase A (90% H_2_O, 10 mM ammonia acetate, 0.2% acetic acid) and mobile phase B (90% ACN, 10 mM ammonia acetate, 0.2% acetic acid). The samples were reconstituted in 1 ml of HILIC buffer (14.74 μM ^13^C_9_-L-tyrosine-^15^N, 42.74 nM ^13^C-L-lactate in 30% mobile phase A and 70% mobile phase B). After centrifugation, 200 μl of the supernatant was transferred to a sampling tube. Samples (2 μl and 10 μl) were subjected to LC–MS/MS analysis in positive and negative ion modes, respectively. Metabolomics analysis was performed with continuous QCs every ten MS injections. The linear gradient was as follows: 1.5 min, 90% B; 5 min, 45% B; 10 min, 45% B; 12 min, 90% B; 25 min, 90% B. The MS parameters were as follows. Positive ion mode: curtain gas (GUR), 35.0; collision gas (CAD), medium; ion spray voltage (IS), 5,500. Negative ion mode: curtain gas (GUR), 35.0, collision gas (CAD), medium; ion spray voltage (IS), −4,500. The temperature was set to 400.0 °C, and ion source gas 1 (GS1) was set to 55.0.

The raw files of the targeted metabolomics data were analyzed by using SCIEX OS software based on a homemade metabolite database. With the Analytics module of SCIEX OS software, the peak area and retention time of each metabolite in each sample were checked manually. Metabolites with CV values ≥ 0.2 for longitudinal QC replicates were removed. To normalize the weight of the samples, the intensity of each metabolite was divided by the corresponding correction factor of that sample (correction factor = weight of the sample / average weight of all samples). Subsequently, the intensity of each metabolite was corrected using the average intensity of the metabolite in the QC samples before and after MS acquisition. The targeted metabolomic data of *M. fascicularis* large intestines satisfy a normal distribution.

### Analysis of iron content in CRC tissues

CRC tissues were lysed with RIPA buffer (50 mM Tris (pH 7.5), 150 mM NaCl, 0.5% sodium deoxycholate, 1% NP40, 25 mM nicotinamide, 10 mM sodium butyrate, 1% protease inhibitor cocktail, 1% phosphatase inhibitor A and 1% phosphatase inhibitor B) and homogenized with a gentleMACS Dissociator. The crude lysates were then sonicated on ice for 5 min (25% power, 3 s on and 10 s off), and the protein concentration was determined by Bradford assay (Bio-Rad, 5000205). Then, 400 μl of supernatant was transferred to a new Eppendorf tube and freeze-dried. The lyophilized samples were added to 50 μl of nitric acid (HNO_3_) (65% Suprapur, Merck) and digested overnight at room temperature. After heating (90 °C) for 20 min, an equivalent volume of hydrogen peroxide (H_2_O_2_) (30% Aristar, BDH) was added to each sample. Reheat for 15 min at 70 °C. Then, the samples were diluted with 1% HNO_3_ to 1 ml. Measurements were performed using an Agilent 7700 series ICP-MS instrument under routine multi-element operating conditions using a helium reaction gas cell.

### Statistics and reproducibility

The proteomic, transcriptomic, phosphoproteomic and targeted metabolomic data from *M. fascicularis* large intestinal tissues follow a normal distribution. Untargeted metabolomic data of *M. fascicularis* large intestinal tissues were not normally distributed. Normality was tested using the Shapiro–Wilk test. Differentially regulated genes or metabolites were screened according to statistical principles. For normally distributed data, the differences among the three age stages were tested by one-way ANOVA, and the differences between any two groups were tested by two-sided Student’s *t*-test. If the data did not conform to a normal distribution, the Kruskal–Wallis test was used to test the differences among the three age groups, and the Wilcoxon rank-sum test was used to test the differences between two groups. Correlations between two sets of data were evaluated using either Spearman’s correlation coefficient or Pearson’s correlation coefficient. For comparative analysis of the ascending and descending colon, two-tailed paired *t*-tests or Wilcoxon signed-rank paired tests were used. To address sample balance issues in the differential analysis, we carefully balanced samples for each analysis by selecting an equal number of samples from each animal. To enhance transparency regarding the sample balance status for each comparative analysis, we provided specific information about the samples used in the differential analysis data in the Source Data files. Gene Ontology (GO) and KEGG enrichment analyses were based on DAVID Bioinformatics Resources. For phosphoproteomics, the Metascape database was used for pathway analysis^94^. For metabolomic enrichment analyses, metabolic pathway data were downloaded from the KEGG PATHWAY Database (https://www.genome.jp/kegg/pathway.html). Subsequently, pathway enrichment analysis was performed using the ‘enricher’ function within the R package ‘clusterProfiler’. Data analysis and graphing were performed using R version 4.1.0 and GraphPad Prism 8.0. For the cell and *C. elegans* experiments, three or more independent experiments were performed for each group. In mouse experiments, we ensured that the number of mice in each group was equal to or greater than seven. For all the experiments, no statistical method was used to pre-determine the sample size. All the experimental animal groupings were randomized.

### Cell culture

Caco-2 and HEK293T cell lines were obtained from the Cell Resource Center, Peking Union Medical College (the headquarters of the National Science and Technology Infrastructure–National BioMedical Cell-Line Resource). Caco-2 cells were cultured in MEM-EBSS supplemented with 20% FBS, 1% NEAA (non-essential amino acids), 10 mM HEPES, 100 units of penicillin and 100 mg ml^−1^ streptomycin at 37 °C and 5% CO_2_. HEK293T cells were cultured in DMEM supplemented with 10% FBS, 100 units of penicillin and 100 mg ml^−1^ streptomycin at 37 °C in 5% CO_2_.

### Western blotting analysis

The retroviral plasmid MSCV-cDNA-IRES-GFP containing the human wild-type CTNND1, CTNND1^S349A^ mutant and CTNND1^S349E^ mutant genes was constructed. To produce retroviral particles, the plasmid for the gene, the packaging plasmid pCL-Eco and the envelope plasmid pVSV-G were co-transfected into 293T cells. Caco-2 cells were transfected with retroviral particles for 24 h. After 24 h, the transfection efficiency was assessed based on the fluorescence intensity of the cells. After 10 days of culture, the Caco-2 cells were collected. Then, the Caco-2 cells were lysed with RIPA buffer, and the protein concentrations were determined by the Bradford assay. Protein samples dissolved in 1× loading buffer (10% glycerol, 2% SDS, 1% 2-mercaptoethanol, 5% 1 M Tris (pH 6.8)) were separated by 12% SDS–PAGE and then transferred to polyvinylidene difluoride membranes (Millipore, ISEQ00010). The polyvinylidene difluoride membrane was blocked with 4% milk in PBST for 1 h at room temperature and then incubated overnight at 4 °C with the corresponding primary antibody. The membrane was washed with PBST buffer five times for 5 min each time. Then, the membrane was incubated with the secondary antibody at room temperature for 1 h. The membrane was washed with PBST for 5 min each time. After being washed five times, the membrane was subjected to chemiluminescent detection (Millipore, WBKLS0500). CLDN1 (HuaBio, ER1906-37, 1:1,000 dilution), TJP1 (Proteintech, 21773-1-AP, 1:5,000 dilution), JAM1 (HuaBio, ET1610-90, 1:1,000 dilution), CTNND1 (HuaBio, ER1803-71, 1:500 dilution), OCLN (HuaBio, R1510-33, 1:1,000 dilution), CTNNB1 (HuaBio, ET1601-5, 1:1,000 dilution), CTNNA1 (HuaBio, ER62912, 1:1,000 dilution), CDH1 (HuaBio, ET1607-75, 1:1,000 dilution) and GAPDH (Proteintech, 60004-1-Ig, 1:10,000 dilution) antibodies were used for western blotting analysis.

### Treatment of DSS-induced colitis with chemicals

C57BL/6 male mice (8 weeks old) were fed for 7 days before the treatment was started. Mice were pre-treated with metabolites (Trp (50 mg/kg/d), 5-HT (20 mg/kg/d) or LP-533401 (HY-15849A, MedChemExpress) (30 mg/kg/d), intraperitoneally) for 2 days and then administered DSS (3%, w/v) for 7 days (ad libitum) with continued metabolite treatment. The DSS group was given an equal volume of normal saline every day. Seven mice were included in each group. The body weight and disease activity indices of the mice were recorded daily. After DSS treatment was discontinued, the mice were killed after 2 days of continued metabolite treatment, and the colon length of each mouse was measured.

### Histopathology

The ascending and descending colon tissues of the mice were washed with normal saline and fixed with 4% paraformaldehyde. Tissues were embedded in paraffin, cut into 5-μm tissue sections and stained with H&E. Sections were imaged using an automatic digital slide scanner (PANNORAMIC MIDI, 3DHISTECH) and analyzed by CaseViewer (RRID: SCR_017654). The histopathology score was calculated by double-blind scoring of epithelial damage, mononuclear and polymorphonuclear infiltration and submucosal edema (0 = none, no epithelial damage, no inflammatory infiltration; 1 = very mild, mild reduction of goblet cells, no inflammatory infiltration; 2 = mild, multiple epithelial cell loss, mild inflammatory infiltration; 3 = moderate, moderate degree, multifocal crypt epithelial cell loss and inflammatory infiltration; 4 = severe, severe diffuse epithelial cell loss, lamina propria inflammatory infiltration and edema).

### Matching human gene homologs in *C. elegans*

The *C. elegans* homologous genes corresponding to the genes in the National Center for Biotechnology Information (NCBI) database (https://www.ncbi.nlm.nih.gov/homologene) and the WormBase database (https://wormbase.org) were retrieved, and the homologous genes were subsequently filtered. These selected genes were further compared to our RNAi bacterial library, and the resulting genes were subsequently functionally validated in *C. elegans*.

### *C. elegans* culture and strains

*C. elegans* were cultured on standard nematode growth medium (NGM) plates seeded with *Escherichia coli* OP50 or HT115 bacteria at 20 °C (ref. ^95^), unless otherwise specified. The screening worms were cultured at 25 °C and subsequently transferred to RNAi bacteria at the late L4 stage. CF512(*rrf-3*(*b26*)*II*; *fem-1*(*hc17*)*IV*), MS438([*elt-2*::*NLS*::*GFP*::*lacZ*+*rol-6*(*su1006*)]*IV*) and N2 were obtained from the Caenorhabditis Genetics Center. The first day of adulthood was calculated as D1.

### RNAi screening and RNAi assays

RNAi clones from the *C. elegans* Vidal RNAi library (Horizon Discovery) were cultured overnight in LB solution containing ampicillin (0.1 mg ml^−1^) and seeded on NGM culture plates containing 0.1 mg ml^−1^ ampicillin, 1 mM tetracycline and 1 mM IPTG. A total of 40–50 synchronized L4 worms at 25 °C were washed and transferred from OP50 bacteria to plates containing RNAi bacteria using M9 buffer. Bacteria containing the empty vector (L4440) were used as an RNAi control. Worms were cultured at 25 °C for 7 days or 8 days. RNAi assays were performed using the standard RNAi feeding method^96^. Adult worms were transferred daily to exclude interference from offspring.

### Microscopy and imaging analysis

Worms were anesthetized with 1 mM levamisole and imaged immediately. Differential interference contrast (DIC) and fluorescence images were captured with an sCMOS camera (ORCA-Flash4.0 digital camera (Hamamatsu)) and an Olympus BX63 automatic fluorescence microscope. Images were processed and viewed using Olympus cellSens Dimension software.

### Treatment of *C. elegans* with compounds

Various amounts of compounds, including Trp, 5-HT, 5-HIAA, KYN, KYNA, QA and IPA, were dissolved in water to prepare stock solutions. The pH of the stock solution was subsequently adjusted to neutral. For the treatment of *C. elegans*, synchronized worms on D1 were transferred to *E. coli* OP50 plates with different concentrations of compounds. All worms were cultured at 25 °C. Intestinal atrophy and intestinal nuclei were observed at D7, and intestinal leakage was observed at D8. Adult worms were transferred daily to exclude interference from offspring.

### Intestinal atrophy morphology assays

Intestinal atrophy was assessed at 25 °C as previously described^49^. Approximately 30 D7 CF512 worms were placed on agar pads containing 1 mM levamisole, and the intestinal morphology of worms was observed under an Olympus BX63 automatic fluorescence microscope. To assess the impact of age-related genes on intestinal atrophy, synchronized worms were treated with control or targeted gene RNAi. For the genes identified through previous omics screening, we measured the relative intestinal width using the method described in the literature^49^. Specifically, to obtain the relative intestinal width, the luminal width at a section posterior to the pharynx was subtracted from the overall intestinal width, and the resulting value was divided by the body width. The relative intestinal width was then used to assess intestinal atrophy to determine any considerable alterations.

### Intestinal barrier function assay

The Smurf assay in *C. elegans* was performed as previously described^53^. Approximately 30 D8 CF512 worms were taken from each plate and suspended in liquid cultures of standard OP50 bacteria (grown at 37 °C and 220 r.p.m. per minute for 12 h) mixed with blue food dye (Erioglaucine disodium salt, 5.0% w/v in water). Worms were incubated in this mixture for 3 h or 5 h and then washed with M9 until colorless. Subsequently, the worms were transferred to agar pads containing 1 mM levamisole, and the absence of blue food dye in the body cavity was observed under a DIC microscope. Three or more independent experiments were performed for each group, each with 40–50 animals per strain or treatment. The significance of the difference in the proportion of Smurfs between the experimental and control groups was calculated. For genes with significant differences between the experimental and control groups, the mean blue dye intensity in the body cavity of each worm was measured by ImageJ. Specifically, the image was opened using ImageJ, and three random areas without worms were selected for the measurement of the background intensity. The average intensity value corresponding to the posterior part of the pharynx for each worm (approximately the front quarter of the intestine) was calculated, and then the background intensity was subtracted to obtain the actual mean intensity for each individual worm.

### Fluorescence intensity of the intestinal nucleus

The intestinal nucleus assay was performed as previously described^97^. Approximately 15 MS438 worms treated with RNAi or compound were cultured at 25 °C and transferred to 1 mM imidazole at D7. All intestinal nuclei of the worms were imaged under a ×100 objective. The focal length was adjusted at the same exposure time to ensure clarity of the intestinal nuclei. After imaging, the fluorescence intensity of each intestinal nucleus was quantified. Furthermore, for each imaging experiment, images for all timepoints and conditions were acquired on the same day using the same microscope and camera settings.

### *C. elegans* lifespan assay

The lifespan assay was performed at 20 °C. For each group, a total of approximately 120 N2 worms were placed on NGM plates and transferred daily. The first day of adulthood was calculated as D1. Worms with protruding vulvae, that crawled off plates or that exploded were excluded. Worms were counted every day, and those that did not respond to gentle touching were scored dead.

### Reporting summary

Further information on research design is available in the Nature Portfolio Reporting Summary linked to this article.

## Supplementary information

Reporting Summary

Supplementary Table 1 List of 32 crucial regulators confirmed by our functional validation results.

Supplementary Table 2 Age-related metabolites in the gut associated with inflammaging.

## Source data

Source Data Fig. 1 Statistical source data.

Source Data Fig. 2 Statistical source data.

Source Data Fig. 3 Statistical source data.

Source Data Fig. 4 Statistical source data.

Source Data Fig. 5 Statistical source data.

Source Data Fig. 6 Statistical source data.

Source Data Fig. 7 Statistical source data.

Source Data Extended Data Fig. 1 Statistical source data.

Source Data Extended Data Fig. 2 Statistical source data.

Source Data Extended Data Fig. 3 Statistical source data.

Source Data Extended Data Fig. 4 Statistical source data.

Source Data Extended Data Fig. 5 Statistical source data.

Source Data Extended Data Fig. 6 Statistical source data.

Source Data Extended Data Fig. 7 Statistical source data.

Source Data Fig. 4 Unprocessed western blots and original images.

Source Data Fig. 5 Unprocessed western blots and original images.

Source Data Fig. 6 Unprocessed western blots and original images.

Source Data Fig. 7 Unprocessed western blots and original images.

## Data Availability

The mass spectrometry proteomics and phosphoproteomics data of *Macaca fascicularis* have been deposited in the ProteomeXchange Consortium via the iProX partner repository^98,99^ with dataset identifier PXD039361. The mass spectrometry proteomics data of patients with colorectal cancer have been deposited in the ProteomeXchange Consortium via the iProX partner repository with dataset identifier PXD039360. The raw RNA sequencing data have been deposited in the Sequence Read Archive under accession number PRJNA999062. Metabolomics data have been deposited in the European Molecular Biology Laboratory–European Bioinformatics Institute under accession code MTBLS7612 (ref. ^100^). All data relating to the results of this study are available upon reasonable request from the corresponding authors. Source data are provided with this paper.
