# Author Correction: Age-, sex- and proximal–distal-resolved multi-omics identifies regulators of intestinal aging in non-human primates

**DOI:** 10.1038/s43587-026-01258-0

**Published:** 2026-10-06

**Authors:** Xinyuan Wang, Yaru Luo, Siyu He, Ying Lu, Yanqiu Gong, Li Gao, Shengqiang Mao, Xiaohui Liu, Na Jiang, Qianlun Pu, Dan Du, Yang Shu, Shan Hai, Shuangqing Li, Hai-Ning Chen, Yi Zhao, Dan Xie, Shiqian Qi, Peng Lei, Hongbo Hu, Heng Xu, Zong-Guang Zhou, Biao Dong, Huiyuan Zhang, Yan Zhang, Lunzhi Dai

**Affiliations:** 1 National Clinical Research Center for Geriatrics, Center for Immunology and Hematology and General Practice Ward/International Medical Center Ward, General Practice Medical Center, State Key Laboratory of Biotherapy, West China Hospital, Sichuan University, Chengdu, China; 2 Advanced Mass Spectrometry Center, Research Core Facility, Frontiers Science Center for Disease-Related Molecular Network, West China Hospital, Sichuan University, Chengdu, China; 3 School of Life Sciences, Tsinghua University, Beijing, China; 4 Colorectal Cancer Center, Department of General Surgery, West China Hospital, Sichuan University, Chengdu, China; 5 Department of Rheumatology and Immunology, State Key Laboratory of Biotherapy, West China Hospital, Sichuan University, Chengdu, China

**Keywords:** Proteomics, Phosphorylation, Ageing

Correction to: *Nature Aging* https://doi.org/10.1038/s43587-024-00572-9, published online 6 February 2024.

In the version of the article initially published, in column AM of the source data for Fig. 3f, female proximal data were incorrectly included in place of male distal data. The source data have now been corrected. In Fig. 5m, the significance annotations for DTD1, HPX and SH3BGR were “***” instead of “*”; that for SPATA5 was “***” instead of “**”; those for ATP1B1 and L1CAM were “**” instead of “*”; and that for STX1A was “*” instead of “**”. The figure has now been corrected. These corrections do not affect the results or conclusions of the article. This correction has been made to the HTML and PDF versions of the article.

