# Supplementary material for: Age-, sex- and proximal–distal-resolved multi-omics identifies regulators of intestinal aging in non-human primates
Source: Nat Aging. 2024 Feb 6;4(3):414–33. doi: 10.1038/s43587-024-00572-9 (PMC10950786; doi:10.1038/s43587-024-00572-9)
Supplement: Supplementary file 1 — Reporting Summary [file 43587_2024_572_MOESM1_ESM.pdf]

Corresponding author(s): Lunzhi Dai; Yan Zhang; Huiyuan Zhang; Biao Dong

Last updated by author(s): Dec 13, 2023

# Reporting Summary

Nature Portfolio wishes to improve the reproducibility of the work that we publish. This form provides structure for consistency and transparency in reporting. For further information on Nature Portfolio policies, see our Editorial Policies and the Editorial Policy Checklist.

## Statistics

For all statistical analyses, confirm that the following items are present in the figure legend, table legend, main text, or Methods section.

| n/a | Confirmed | |
|---|---|---|
| ☐ | ☒ | The exact sample size (*n*) for each experimental group/condition, given as a discrete number and unit of measurement |
| ☐ | ☒ | A statement on whether measurements were taken from distinct samples or whether the same sample was measured repeatedly |
| ☐ | ☒ | The statistical test(s) used AND whether they are one- or two-sided<br>*Only common tests should be described solely by name; describe more complex techniques in the Methods section.* |
| ☒ | ☐ | A description of all covariates tested |
| ☐ | ☒ | A description of any assumptions or corrections, such as tests of normality and adjustment for multiple comparisons |
| ☐ | ☒ | A full description of the statistical parameters including central tendency (e.g. means) or other basic estimates (e.g. regression coefficient) AND variation (e.g. standard deviation) or associated estimates of uncertainty (e.g. confidence intervals) |
| ☐ | ☒ | For null hypothesis testing, the test statistic (e.g. *F*, *t*, *r*) with confidence intervals, effect sizes, degrees of freedom and *P* value noted<br>*Give P values as exact values whenever suitable.* |
| ☒ | ☐ | For Bayesian analysis, information on the choice of priors and Markov chain Monte Carlo settings |
| ☒ | ☐ | For hierarchical and complex designs, identification of the appropriate level for tests and full reporting of outcomes |
| ☐ | ☒ | Estimates of effect sizes (e.g. Cohen's *d*, Pearson's *r*), indicating how they were calculated |

*Our web collection on statistics for biologists contains articles on many of the points above.*

## Software and code

Policy information about availability of computer code

| | |
|---|---|
| Data collection | Raw files for proteomics and phosphoproteomics were searched using MaxQuant (version 1.6). Raw files of untargeted metabolomics were searched by using Tracefinder (Thermo, CA) based on a home-made metabolite database. Raw files of targeted metabolomics were analyzed by using SCIEX OS software (Version 2.1.6.59781) based on a home-made metabolite database. Sections were imaged using an automatic digital slide scanner (PANNORAMIC MIDI, 3DHISTECH). DIC and fluorescence images were captured with an sCMOS camera ORCA-Flash4.0 digital camera (Hamamatsu) and an Olympus BX63 automatic fluorescence microscope. |
| Data analysis | Unsupervised k-means clustering analyses, principal component analysis (PCA), partial least squares discriminant analyses, spearman correlation analysis were implemented in R (version 4.1.0). GO and KEGG enrichment analysis were based on DAVID Bioinformatics Resources. For phosphoproteomics, the Metascape database was used for pathway analysis. Graphing was performed using R version 4.1.0 and Graphpad prism 8.0. Sections were analyzed by CaseViewer (RRID: SCR_017654). DIC and fluorescence images were processed and viewed using Olympus cellSens Dimension software (version 1.18). |

For manuscripts utilizing custom algorithms or software that are central to the research but not yet described in published literature, software must be made available to editors and reviewers. We strongly encourage code deposition in a community repository (e.g. GitHub). See the Nature Portfolio guidelines for submitting code & software for further information.

# Data

Policy information about availability of data

All manuscripts must include a data availability statement. This statement should provide the following information, where applicable:

- Accession codes, unique identifiers, or web links for publicly available datasets
- A description of any restrictions on data availability
- For clinical datasets or third party data, please ensure that the statement adheres to our policy

https://www.iprox.cn/page/PSV023.html;?url=1702264133127qmNF, password: fsWw, proteomics and phosphoproteomics data of Macaca fascicularis (accession number PXD039361).
https://www.iprox.cn/page/PSV023.html;?url=1702263926918qksP, password: 2YUJ, proteomics data of colorectal cancer (accession number PXD039360).
https://dataview.ncbi.nlm.nih.gov/object/PRJNA999062?reviewer=obnbjooik17pgnfel3g0f46pje, Raw RNA-Seq data of Macaca fascicularis (accession number PRJNA999062).
https://www.ebi.ac.uk/metabolights, Raw metabolomics data of Macaca fascicularis (accession number MTBLS7612).

# Human research participants

Policy information about studies involving human research participants and Sex and Gender in Research.

| | |
|---|---|
| Reporting on sex and gender | Gender information is available in Source Data File. |
| Population characteristics | Studies include patient information with age, region in colon, vital status and Overall survival (months). Clinical information of 104 CRC patients is reported in Source Data File. |
| Recruitment | All patients were recruited randomly, and there was no self-selection bias or other biases. Any patient undergoing colorectal cancer surgery in the Department of Gastrointestinal Surgery during the recruitment period was recruited. |
| Ethics oversight | The collection and use of human colorectal cancer (CRC) samples was approved by the Ethics Committee of Biology Research, West China Hospital at Sichuan University (2020(374)), and informed consent was obtained from all participants or their families. |

Note that full information on the approval of the study protocol must also be provided in the manuscript.

# Field-specific reporting

Please select the one below that is the best fit for your research. If you are not sure, read the appropriate sections before making your selection.

☒ Life sciences ☐ Behavioural & social sciences ☐ Ecological, evolutionary & environmental sciences

For a reference copy of the document with all sections, see nature.com/documents/nr-reporting-summary-flat.pdf

# Life sciences study design

All studies must disclose on these points even when the disclosure is negative.

| | |
|---|---|
| Sample size | This study did not employ any statistical methods for sample size calculation. For the multi-omics analyses of large intestinal tissues from 26 Macaca fascicularis and the proteomic analysis of CRC tissues, sample sizes were chosen based on data availability and on previous studies. All available samples passing the quality control were included. For the cell line experiments, ≥ 3 biological replicates were performed. For the mice experiments, ≥ 7 mice were included in each group. For the C. elegans experiments, each experimental group consisted of more than 13 animals in each trial, and each experiment was repeated independently three or more times. |
| Data exclusions | For transcriptomics, the mRNA data in downstream analyses were filtered for genes with an average TPM > 1. For proteomics, the proteins with unique peptide < 2 were removed. And the proteins detected in ≥ 50% samples in each subgroup (RY, RM, RO, LY, LM and LO) were used for subsequent analysis. For phosphoproteomics, phosphopeptides detected in ≥ 50% samples were used for subsequent analysis. |
| Replication | For the cell line and C. elegans experiments, ≥ 3 biological replicates were performed. |
| Randomization | In this study, all cells, C. elegans, mice, and Macaca fascicularis were randomly assigned to experimental and control groups. Patients were also recruited randomly, and there was no selection bias. |
| Blinding | For sample processing and histopathology scoring, all investigators were blinded to the outcome. |

# Reporting for specific materials, systems and methods

We require information from authors about some types of materials, experimental systems and methods used in many studies. Here, indicate whether each material, system or method listed is relevant to your study. If you are not sure if a list item applies to your research, read the appropriate section before selecting a response.

## Materials & experimental systems

| n/a | Involved in the study |
| --- | --- |
| ☐ | ☒ Antibodies |
| ☐ | ☒ Eukaryotic cell lines |
| ☒ | ☐ Palaeontology and archaeology |
| ☐ | ☒ Animals and other organisms |
| ☒ | ☐ Clinical data |
| ☒ | ☐ Dual use research of concern |

## Methods

| n/a | Involved in the study |
| --- | --- |
| ☒ | ☐ ChIP-seq |
| ☒ | ☐ Flow cytometry |
| ☒ | ☐ MRI-based neuroimaging |

## Antibodies

Antibodies used

anti-CLDN1 rabbit polyclonal antibody ( Cat # ER1906-37, lot: A110091815, Huabio, 1:1000 dilution)
anti-ZO-1 polyclonal antibody (Cat #21773-1-AP, lot: 00106141, Proteintech, 1:5000 dilution)
anti-JAM1 recombinant rabbit monoclonal antibody (Cat #ET1610-90, lot: HO0315,clone number: SC60-07, Huabio, 1:1000 dilution)
anti-CTNND1 rabbit polyclonal antibody (Cat #ER1803-71, lot: HM0118, Huabio, 1:500 dilution)
anti-OCLN rabbit polyclonal antibody (Cat #R1510-33, lot: HN1019, Huabio, 1:1000 dilution)
anti-CTNNB1 recombinant rabbit monoclonal antibody (Cat #ET1601-5, lot: HO1109, clone number: SA30-04, Huabio, 1:1000 dilution)
anti-CTNNA1 rabbit polyclonal antibody (Cat #ER62912, lot: HO0315, Huabio, 1:1000 dilution)
anti-CDH1 recombinant rabbit monoclonal antibody (Cat #ET1607-75, lot: HN1224,clone number: SY0287, Huabio, 1:1000 dilution)
anti-GAPDH monoclonal antibody (Cat #60004-1-Ig, lot: 10021642, clone number: 1E6D9, Proteintech, 1:10000 dilution)

Validation

1. Anti-CLDN1: Application statement in manufacturer's website as following: this anti-CLDN1 antibody is validated for use inWB, IHC-P, FC. http://www.huabio.cn/product/Claudin-1-antibody-ER1906-37
2. Anti-ZO-1: Application statement in manufacturer's website as following: this anti-ZO-1 antibody is validated for use in WB, IP, IF, FC. https://www.ptglab.co.jp/products/ZO1-Antibody-21773-1-AP.htm
3. Anti-JAM1: Application statement in manufacturer's website as following: this anti-JAM1 antibody is validated for use in WB, IHC-P. http://www.huabio.cn/product/Junctional-Adhesion-Molecule-1-antibody-ET1610-90
4. Anti-CTNND1: Application statement in manufacturer's website as following: this anti-CTNND1 antibody is validated for use in WB, IF-Cell. http://www.huabio.cn/product/delta-1-Catenin-CAS-antibody-ER1803-71
5. Anti-OCLN: Application statement in manufacturer's website as following: this anti-OCLN antibody is validated for use in WB, IF-Cell, IHC-P, FC. http://www.huabio.cn/product/Occludin-antibody-R1510-33
6. Anti-CTNNB1: Application statement in manufacturer's website as following: this anti-CTNNB1 antibody is validated for use in WB, IHC-P, IF-Tissue, IP. http://www.huabio.cn/product/Beta-Catenin-antibody-ET1601-5
7. Anti-CTNNA1: Application statement in manufacturer's website as following: this anti-CTNNA1 antibody is validated for use in WB, IHC-P, IF. http://www.huabio.cn/product/Catenin-alpha-E-N-antibody-ER62912
8. Anti-CDH1: Application statement in manufacturer's website as following: this anti-CDH1 antibody is validated for use in WB, IF-Cell, IF-Tissue, IHC-P, FC, IP. http://www.huabio.cn/product/E-Cadherin-antibody-ET1607-75
9. Anti-GAPDH: Application statement in manufacturer's website as following: this anti-GAPDH antibody is validated for use in WB, IP, IF, FC. https://www.ptglab.co.jp/products/GAPDH-Antibody-60004-1-Ig.htm

## Eukaryotic cell lines

Policy information about cell lines and Sex and Gender in Research

Cell line source(s)

The Caco-2 cell line and HEK-293T cell line were obtained from the Cell Resource Center, Peking Union Medical College (the headquarter of the National Science & Technology Infrastructure-National BioMedical Cell-Line Resource, NSTI-BMCR).

Authentication

The method used to authenticate cell lines is short tandem repeat (STR) profiling.

Mycoplasma contamination

The mycoplasma contamination test results were negative.

Commonly misidentified lines (See ICLAC register)

No commonly misidentified cell lines were used.

## Animals and other research organisms

Policy information about studies involving animals; ARRIVE guidelines recommended for reporting animal research, and Sex and Gender in Research

| | |
|---|---|
| Laboratory animals | Macaca fascicularis were housed in a standardized laboratory environment maintained at a temperature of approximately 25 °C and a 12 h light and 12 h dark cycle in Canton. Before the experiment, all animals had no clinical or experimental history that might affect physiological aging or increase susceptibility to diseases.<br>C57BL/6 mice (8 weeks old) were ordered from GemPharmatech Co. (Nanjing, China) and housed in a standard specific pathogen-free (SPF) laboratory environment. The mice were kept in a controlled environment with a 12-hour light/dark cycle, maintaining the ambient temperature at 22 ± 2 degrees Celsius and humidity at 50 ± 5%. They had access to standard rodent chow and water at all times. Housing conditions remained uniform across all experimental and control groups.<br>C. elegans were cultured on standard nematode growth medium (NGM) plates seeded with Escherichia coli OP50 or HT115 bacteria at 20 °C, unless otherwise specified. Screening worms were cultured at 25 °C and transferred to RNAi bacteria at the late L4 stage. |
| Wild animals | The study did not involve wild animals. |
| Reporting on sex | In the experiment of treatment of DSS-induced colitis with chemicals, male mice were selected to construct the DSS mouse model. This is because male mice tend to be more susceptible than female mice in DSS colitis[1].<br><br>References:<br>1. Wirtz, Stefan et al. "Chemically induced mouse models of acute and chronic intestinal inflammation." Nature protocols vol. 12,7 (2017): 1295-1309. doi:10.1038/nprot.2017.044 |
| Field-collected samples | The study did not involve field-collected samples. |
| Ethics oversight | This study of Macaca fascicularis was conducted in accordance with the Ethical Treatment of Non-Human Primate which was approved by the Institutional Animal Care and Use Committee at Yuanxi Biotech Inc. in Guangzhou (YXSW-2016-01). The use and care of the mice were approved by the Animal Experiment Ethics Committee of State Key Laboratory of Biotherapy at Sichuan University (Number: 20220531045). |

Note that full information on the approval of the study protocol must also be provided in the manuscript.
