# Supplementary material for: Age-, sex- and proximal–distal-resolved multi-omics identifies regulators of intestinal aging in non-human primates
Source: Nat Aging. 2024 Feb 6;4(3):414–33. doi: 10.1038/s43587-024-00572-9 (PMC10950786; doi:10.1038/s43587-024-00572-9)
Supplement: Supplementary file 18 — Unprocessed western blots and original images. [file 43587_2024_572_MOESM18_ESM.pdf]

The original images of the microscopy and Western blotting.

Fig. 4a

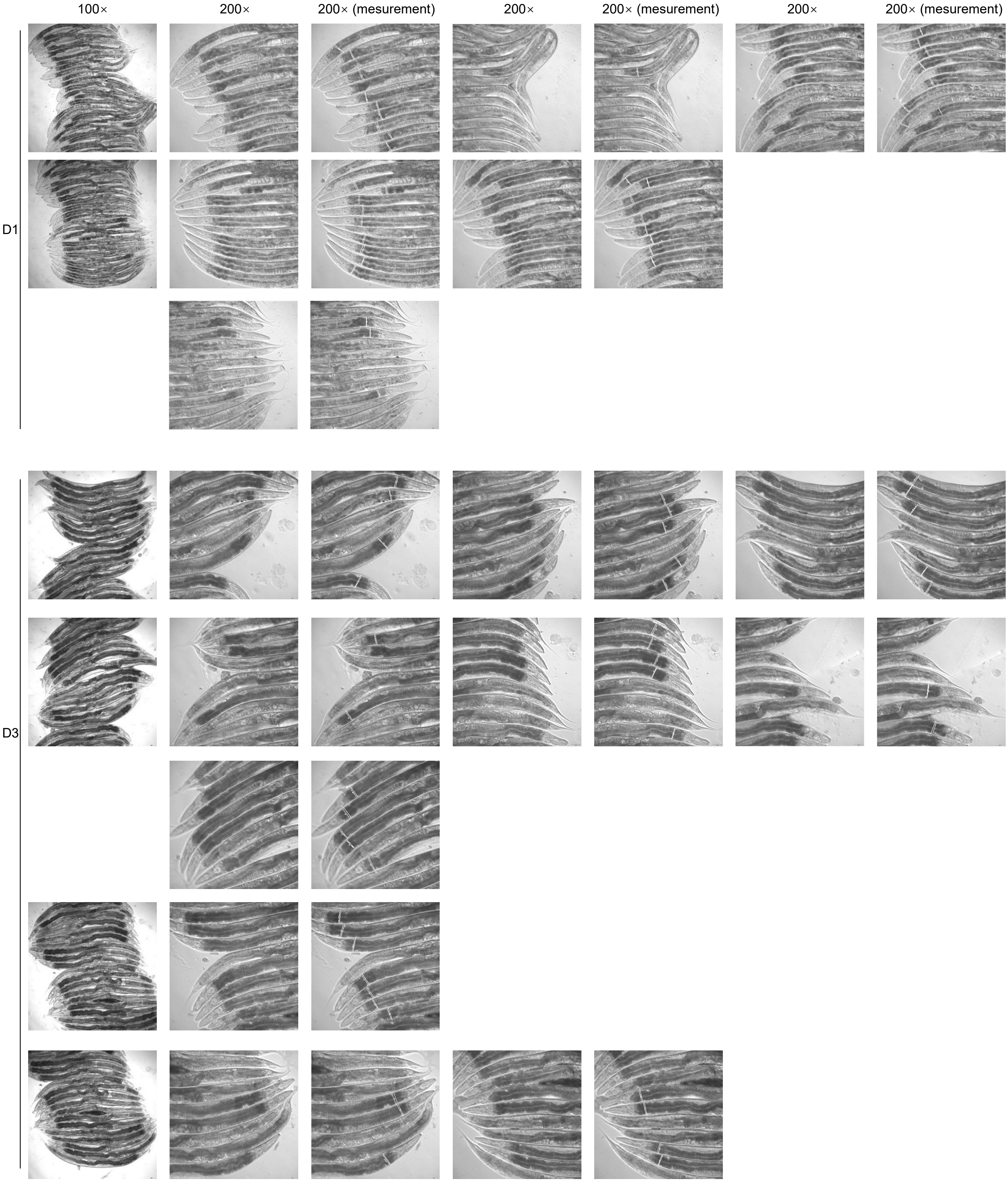

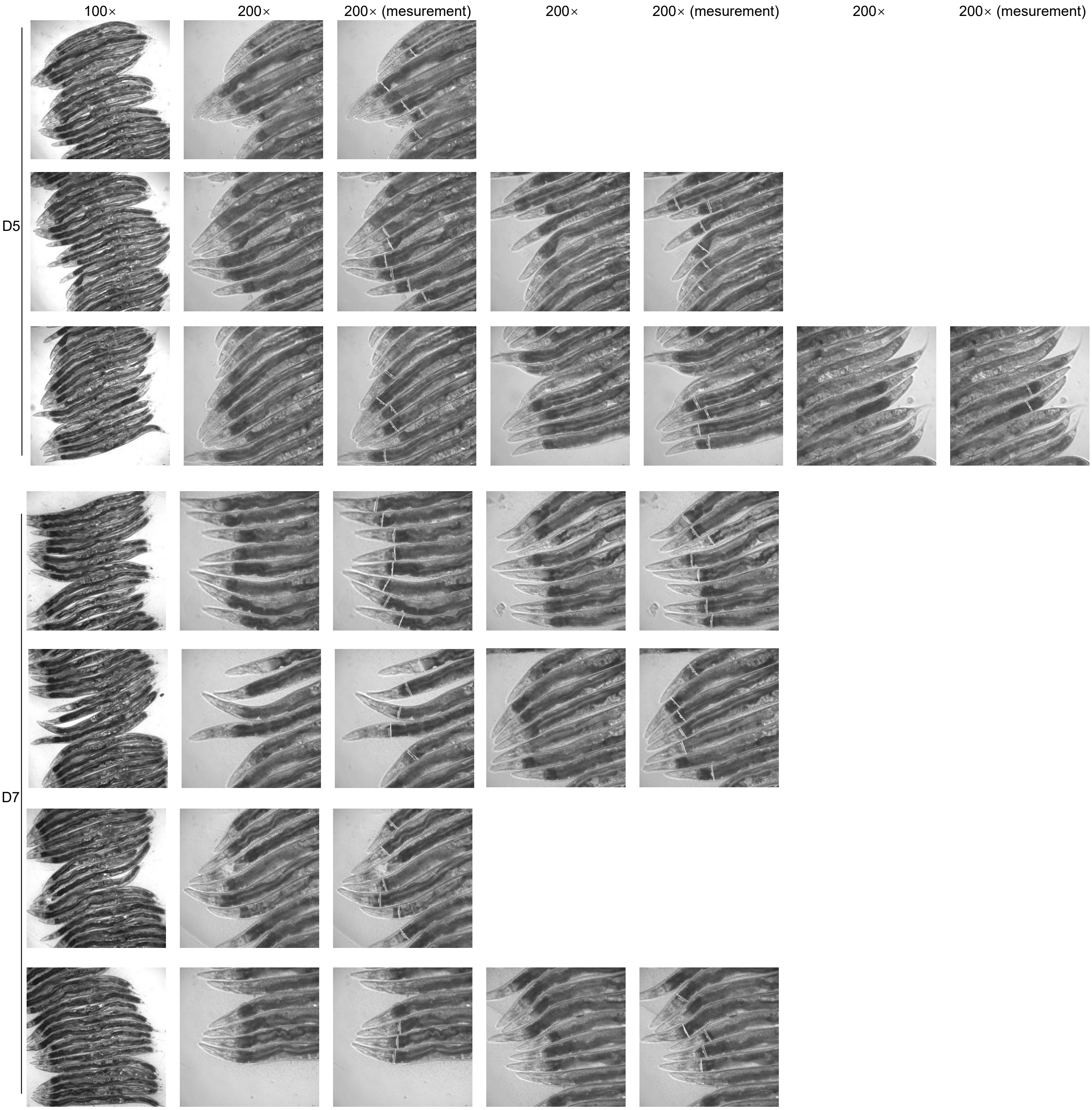
100×
200×
200× (mesurement)
200×
200× (mesurement)
200×
200× (mesurement)
D5
D7

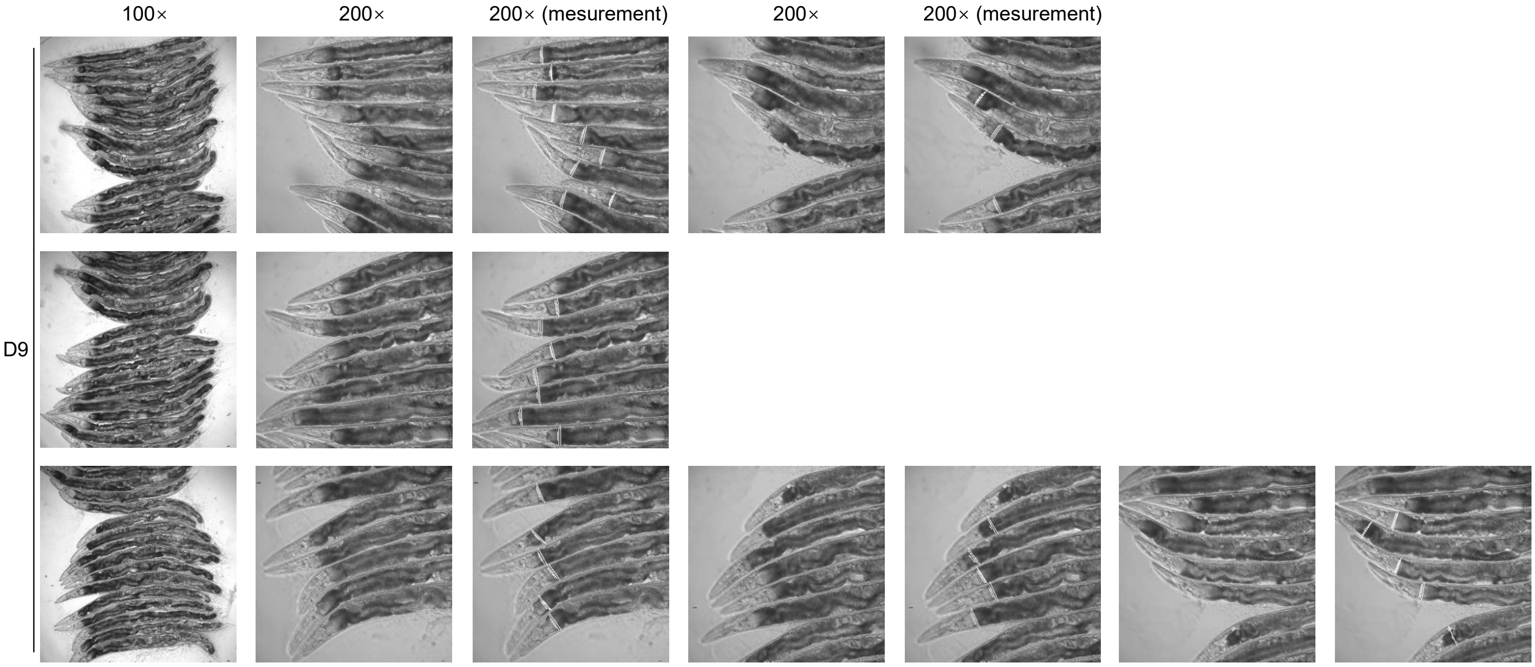
100×
200×
200× (mesurement)
200×
200× (mesurement)
D9

Fig. 4e

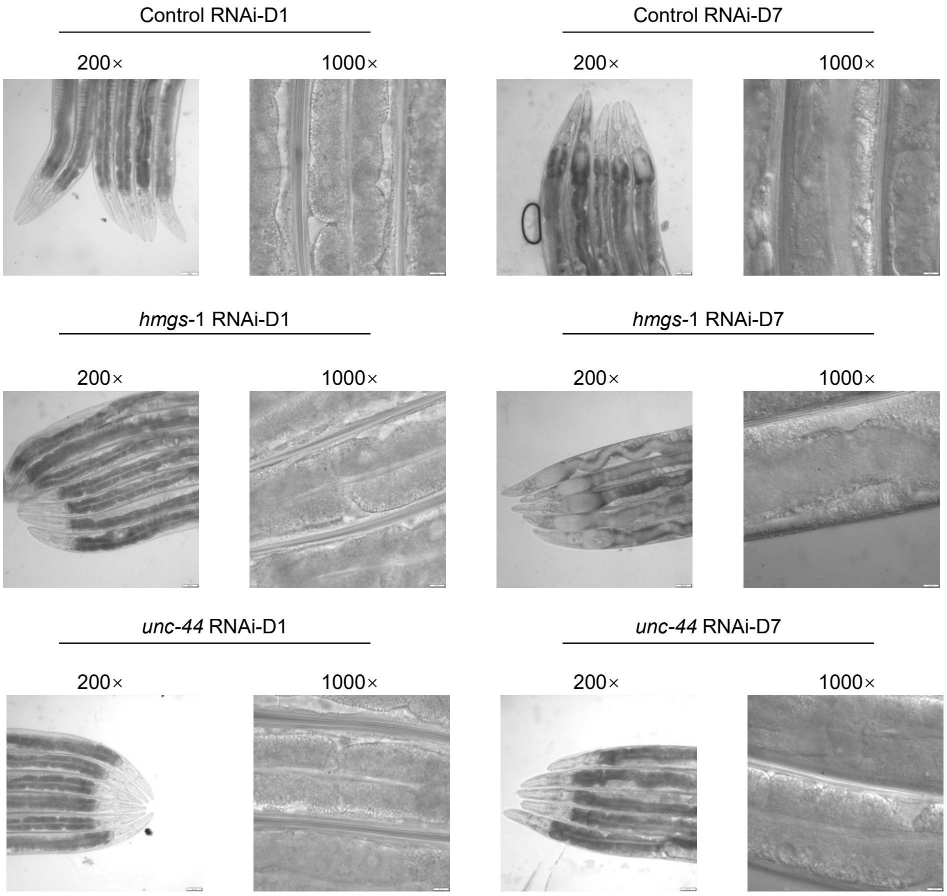

Control RNAi-D1
200×
1000×
Control RNAi-D7
200×
1000×
hmgs-1 RNAi-D1
200×
1000×
hmgs-1 RNAi-D7
200×
1000×
unc-44 RNAi-D1
200×
1000×
unc-44 RNAi-D7
200×
1000×

Fig. 4f-i

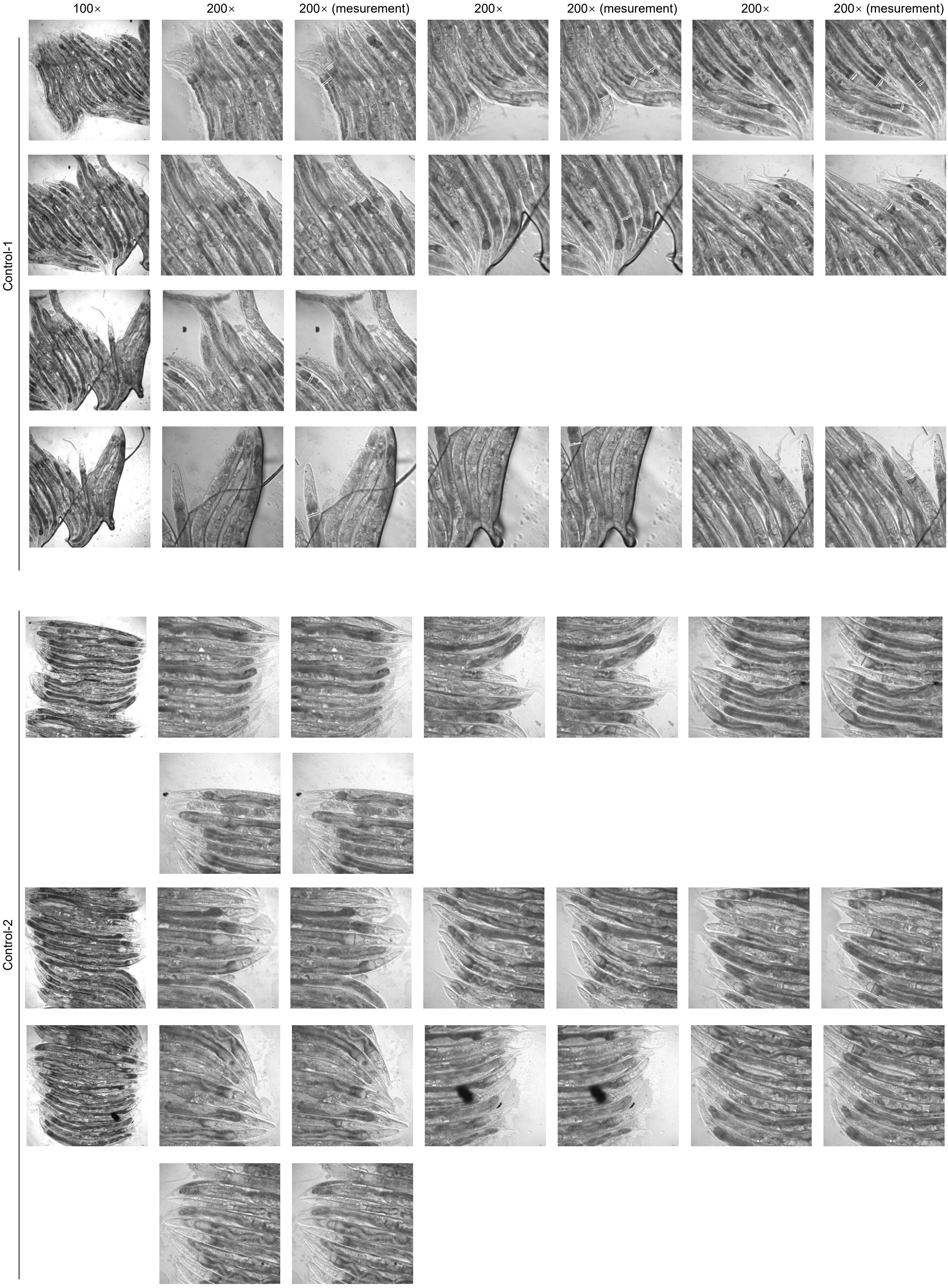
100×
200×
200× (mesurement)
200×
200× (mesurement)
200×
200× (mesurement)
Control-1
Control-2

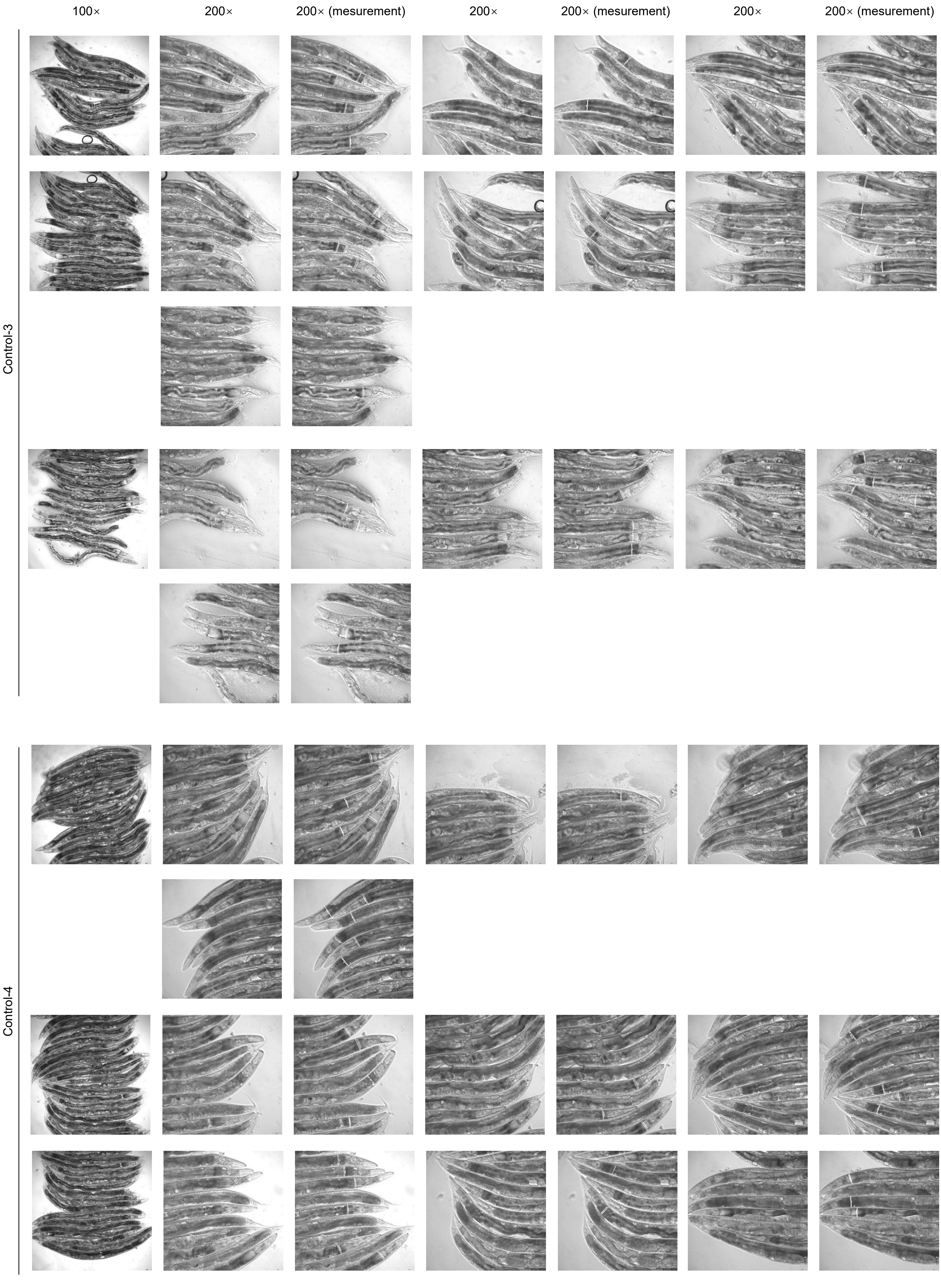
100×
200×
200× (mesurement)
200×
200× (mesurement)
200×
200× (mesurement)
Control-3
Control-4

100× | 200× | 200× (mesurement) | 200× | 200× (mesurement) | 200× | 200× (mesurement)

Control-5

Control-6

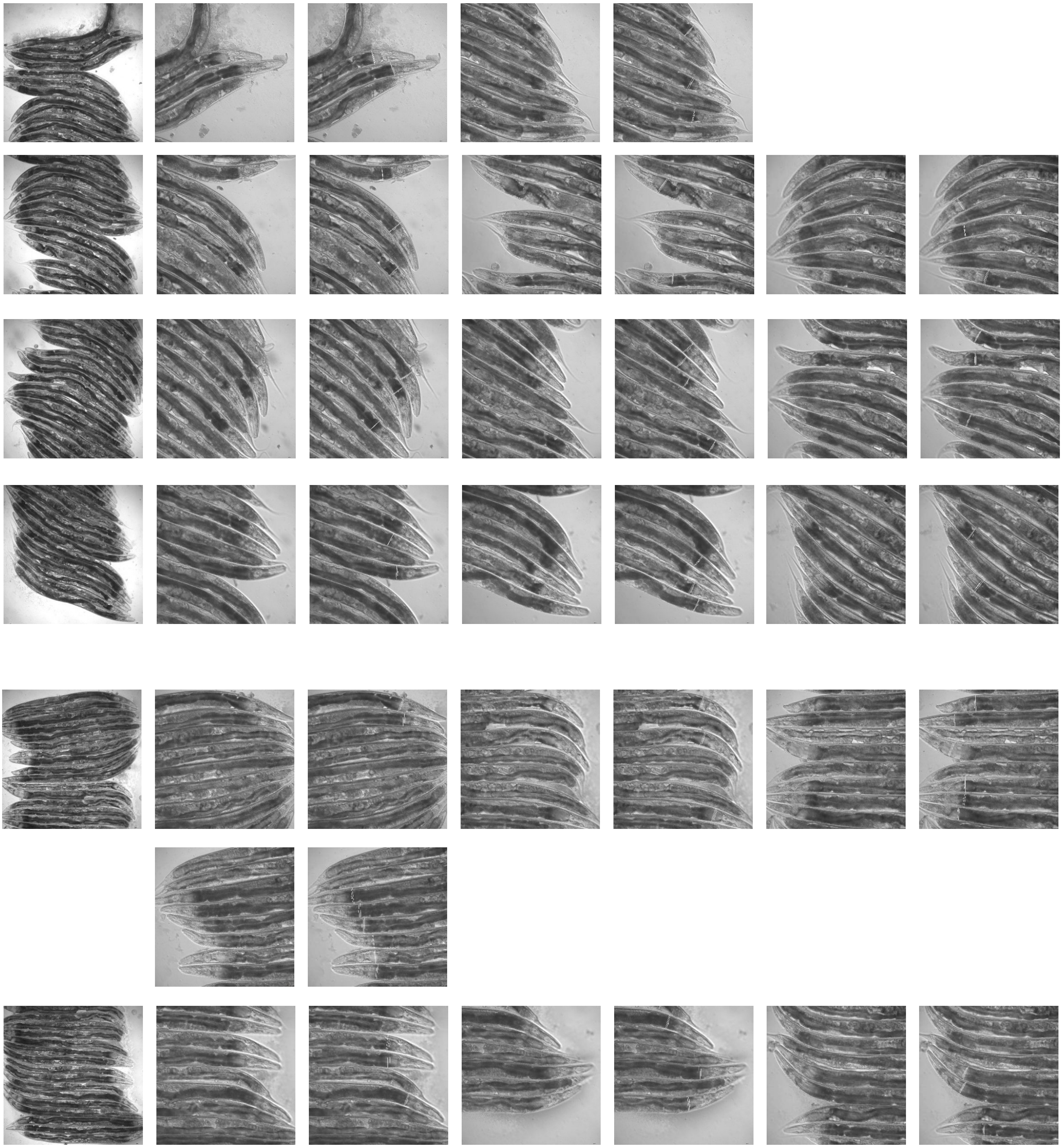

Fig. 4f

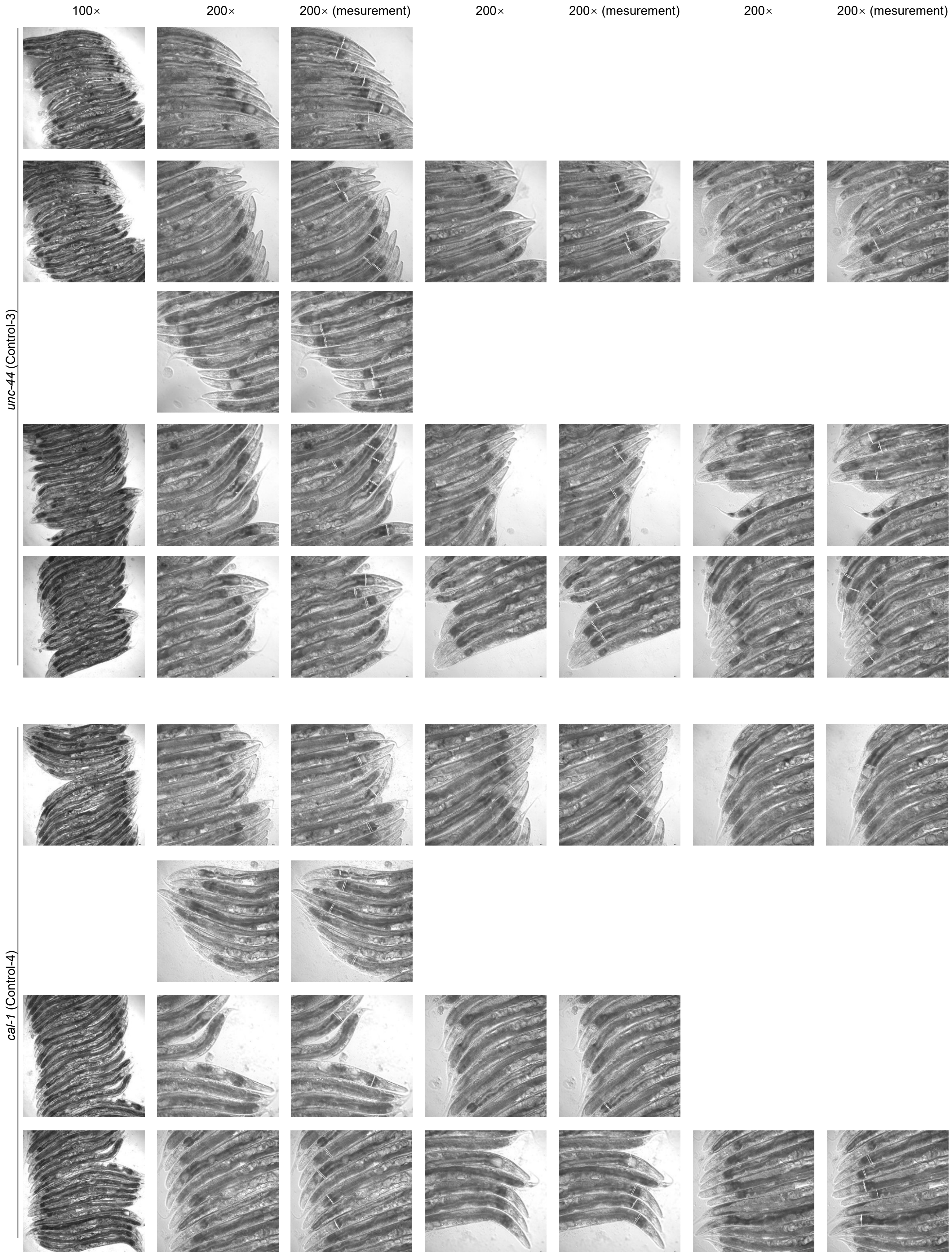
100×
200×
200× (mesurement)
200×
200× (mesurement)
200×
200× (mesurement)
unc-44 (Control-3)
cal-1 (Control-4)

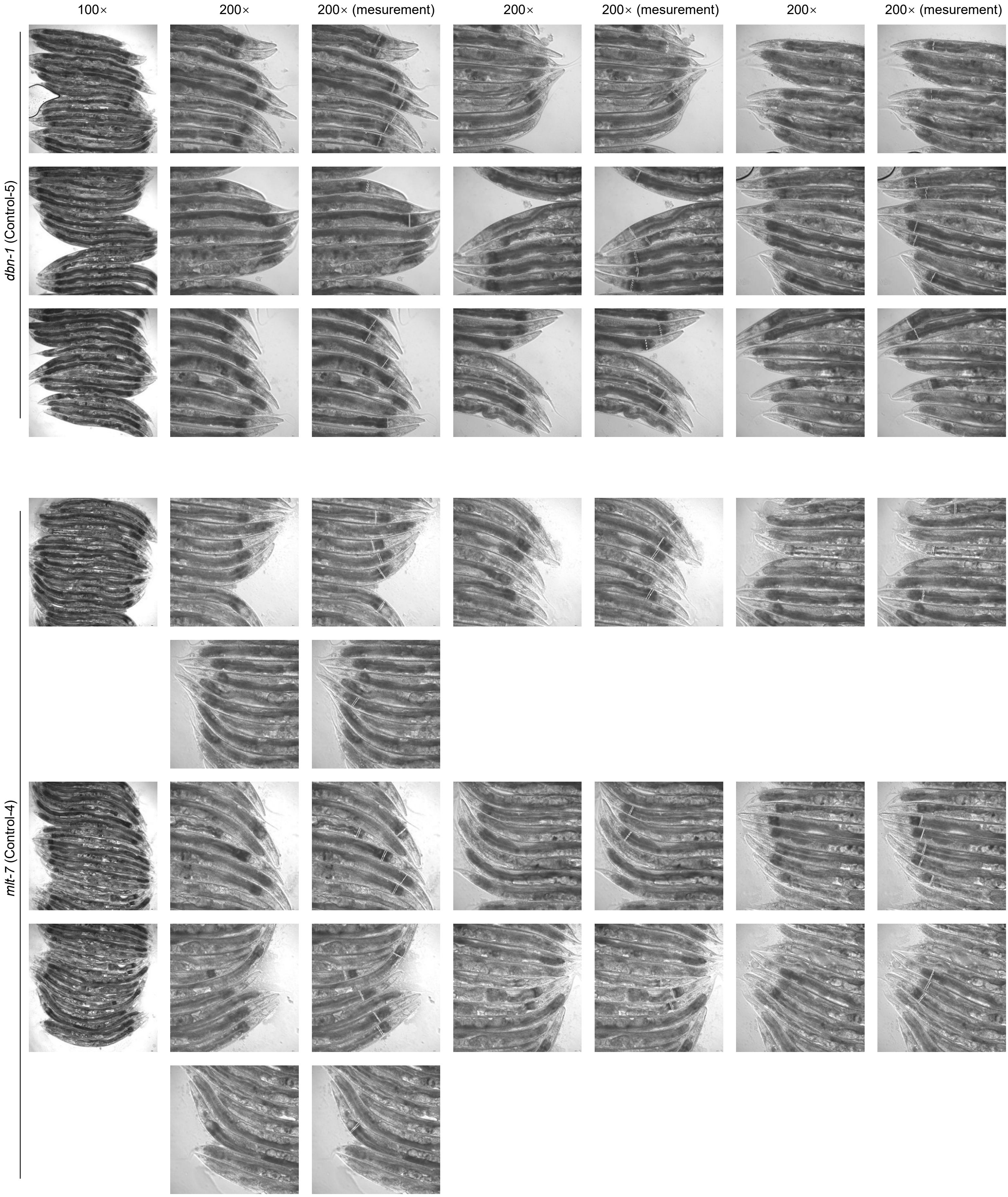
100×
200×
200× (mesurement)
200×
200× (mesurement)
200×
200× (mesurement)
dbn-1 (Control-5)
mlt-7 (Control-4)

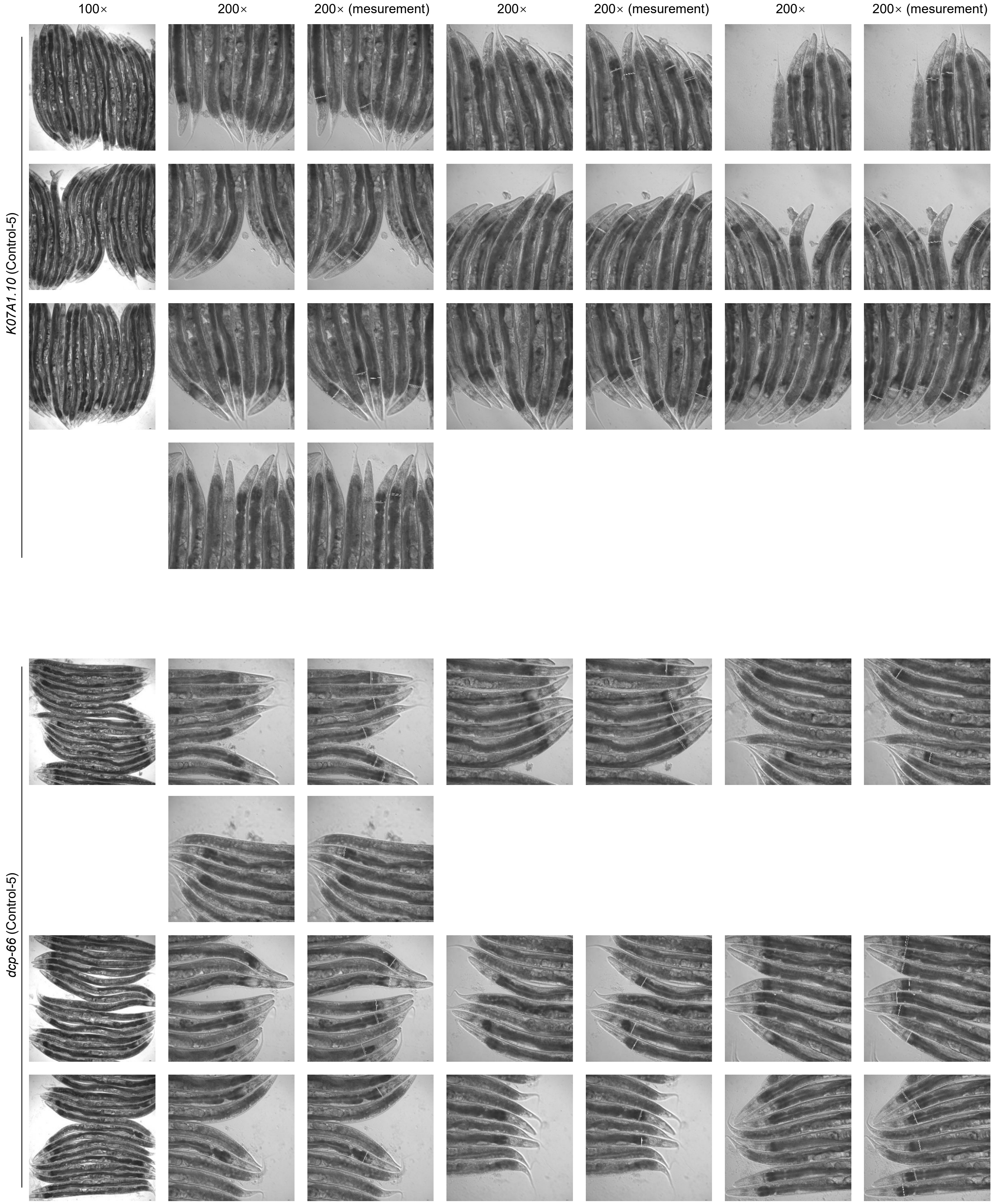
100×
200×
200× (mesurement)
200×
200× (mesurement)
200×
200× (mesurement)
K07A1.10 (Control-5)
dcp-66 (Control-5)

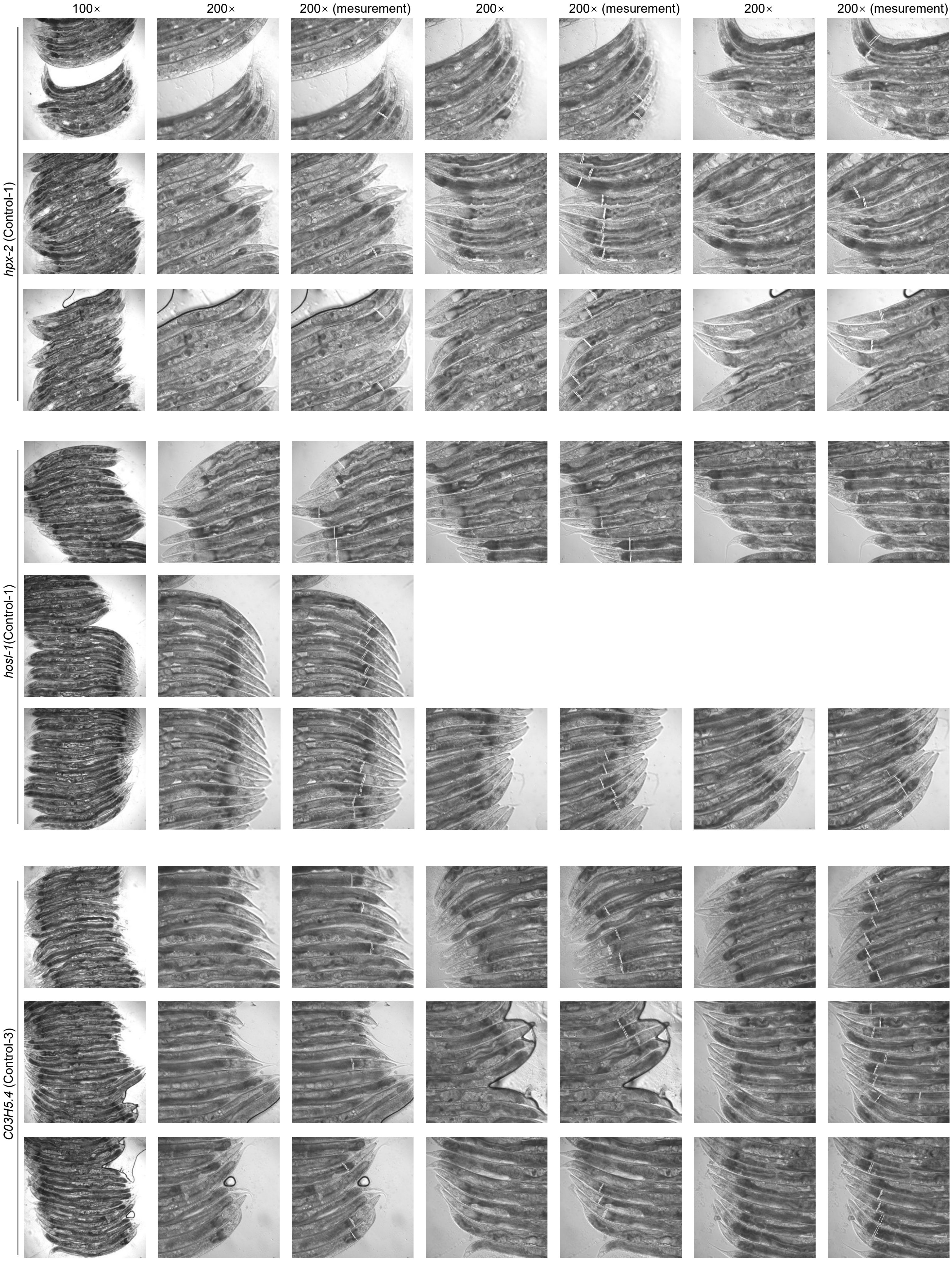
100×
200×
200× (mesurement)
200×
200× (mesurement)
200×
200× (mesurement)
hpx-2 (Control-1)
hosl-1(Control-1)
C03H5.4 (Control-3)

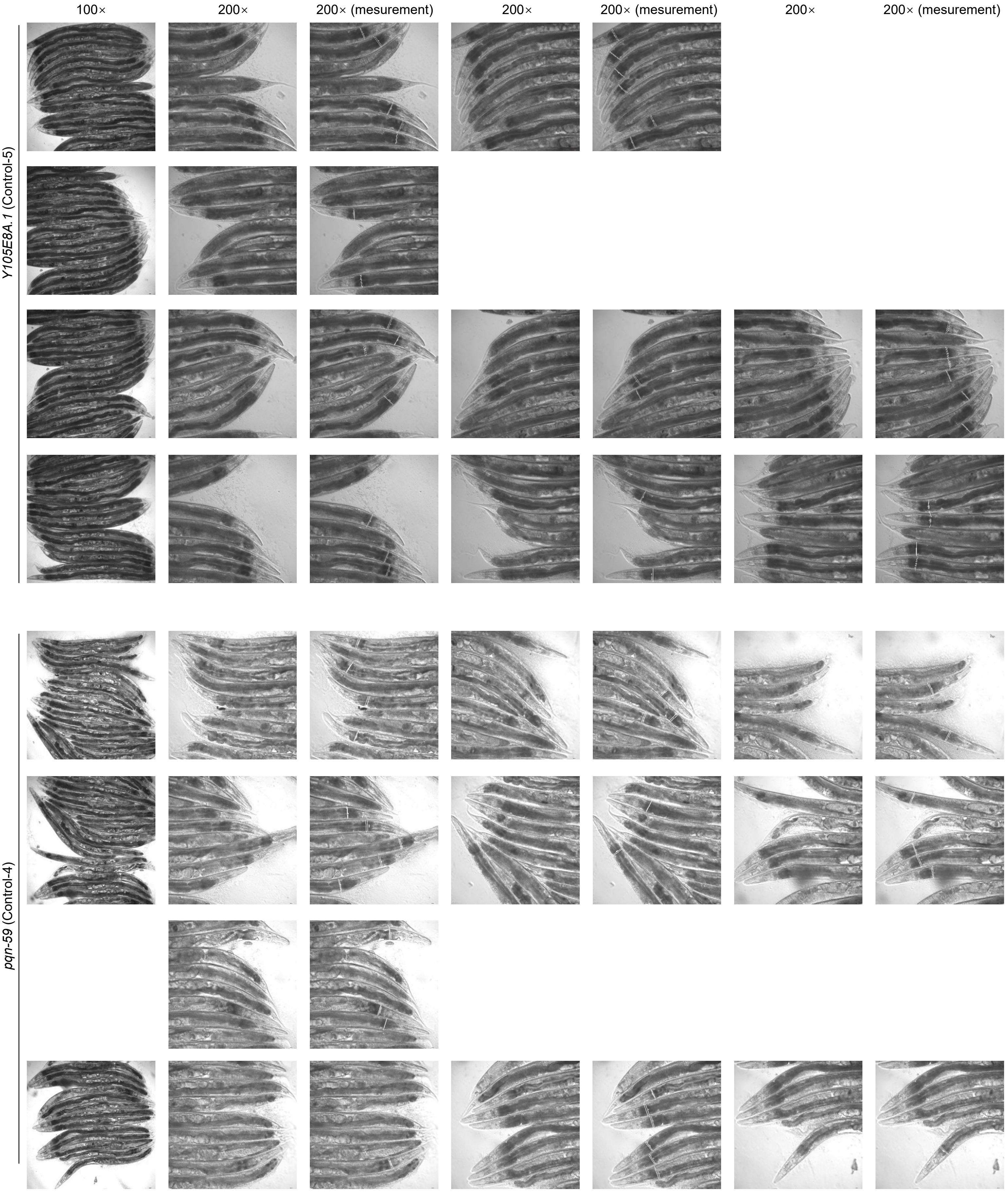
100×
200×
200× (mesurement)
200×
200× (mesurement)
200×
200× (mesurement)
Y105E8A.1 (Control-5)
pqn-59 (Control-4)

Fig. 4g

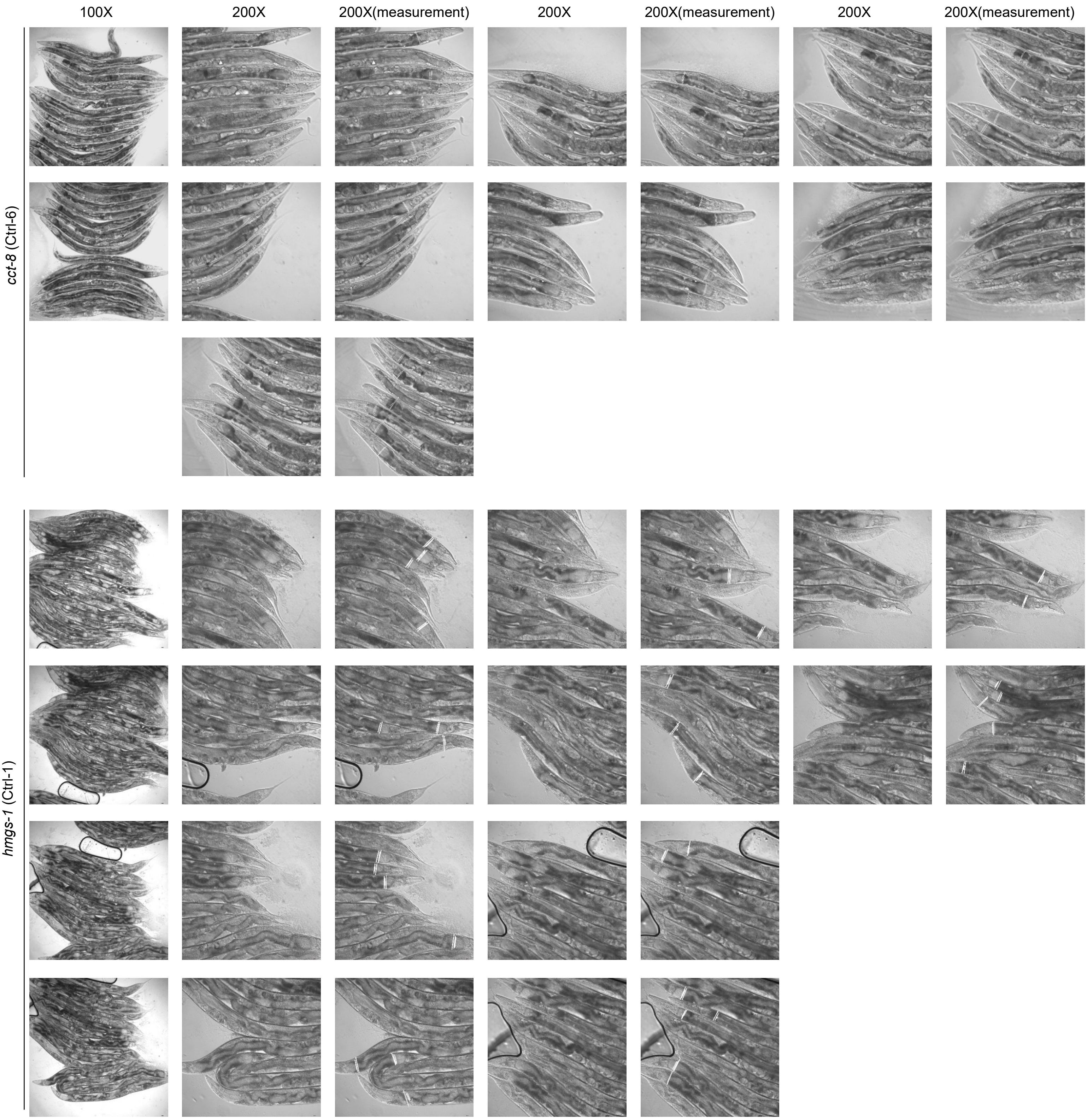
100X
200X
200X(measurement)
200X
200X(measurement)
200X
200X(measurement)
cct-8 (Ctrl-6)
hmgs-1 (Ctrl-1)

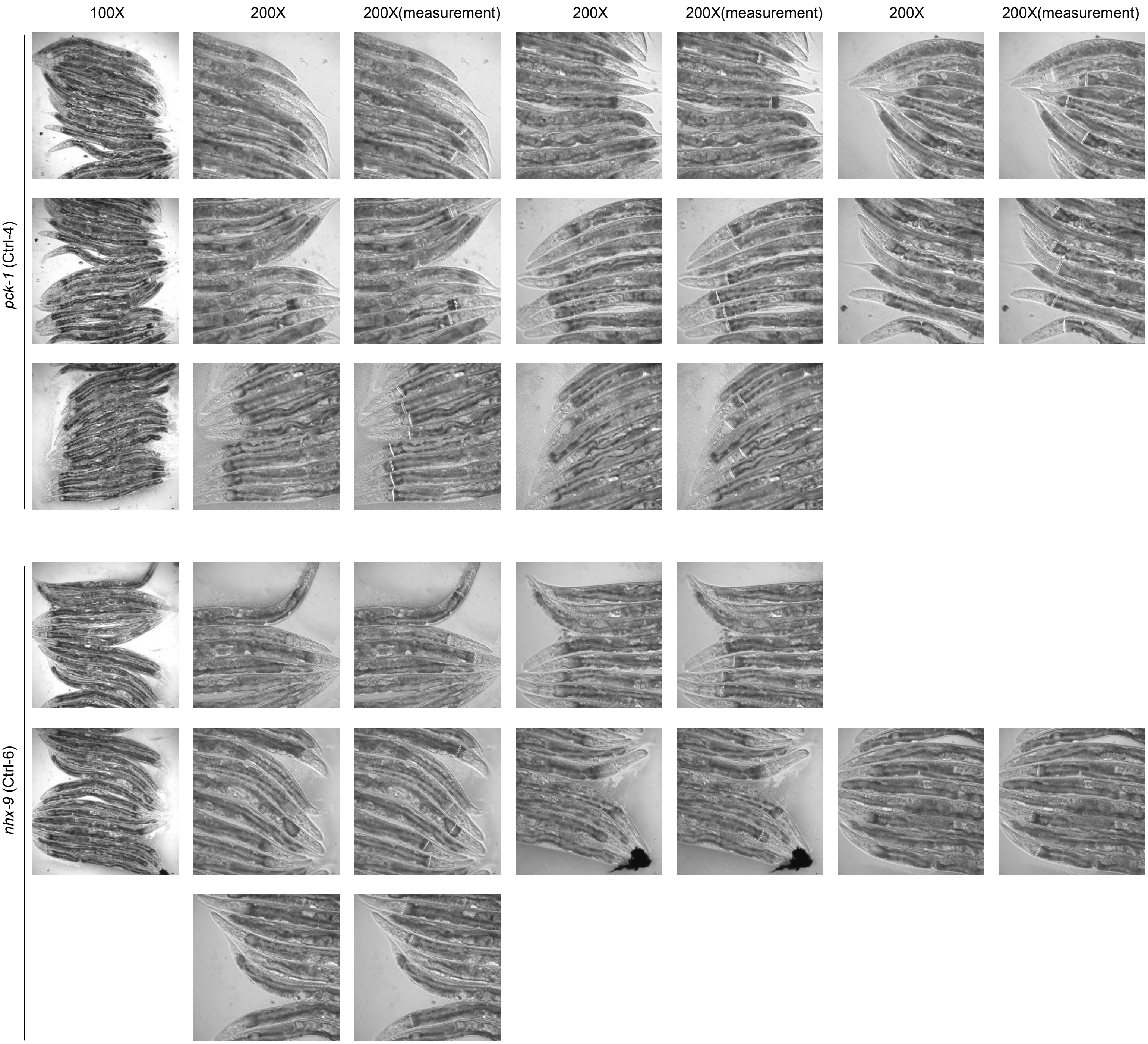
100X
200X
200X(measurement)
200X
200X(measurement)
200X
200X(measurement)
pck-1 (Ctrl-4)
nhx-9 (Ctrl-6)

Fig. 4h

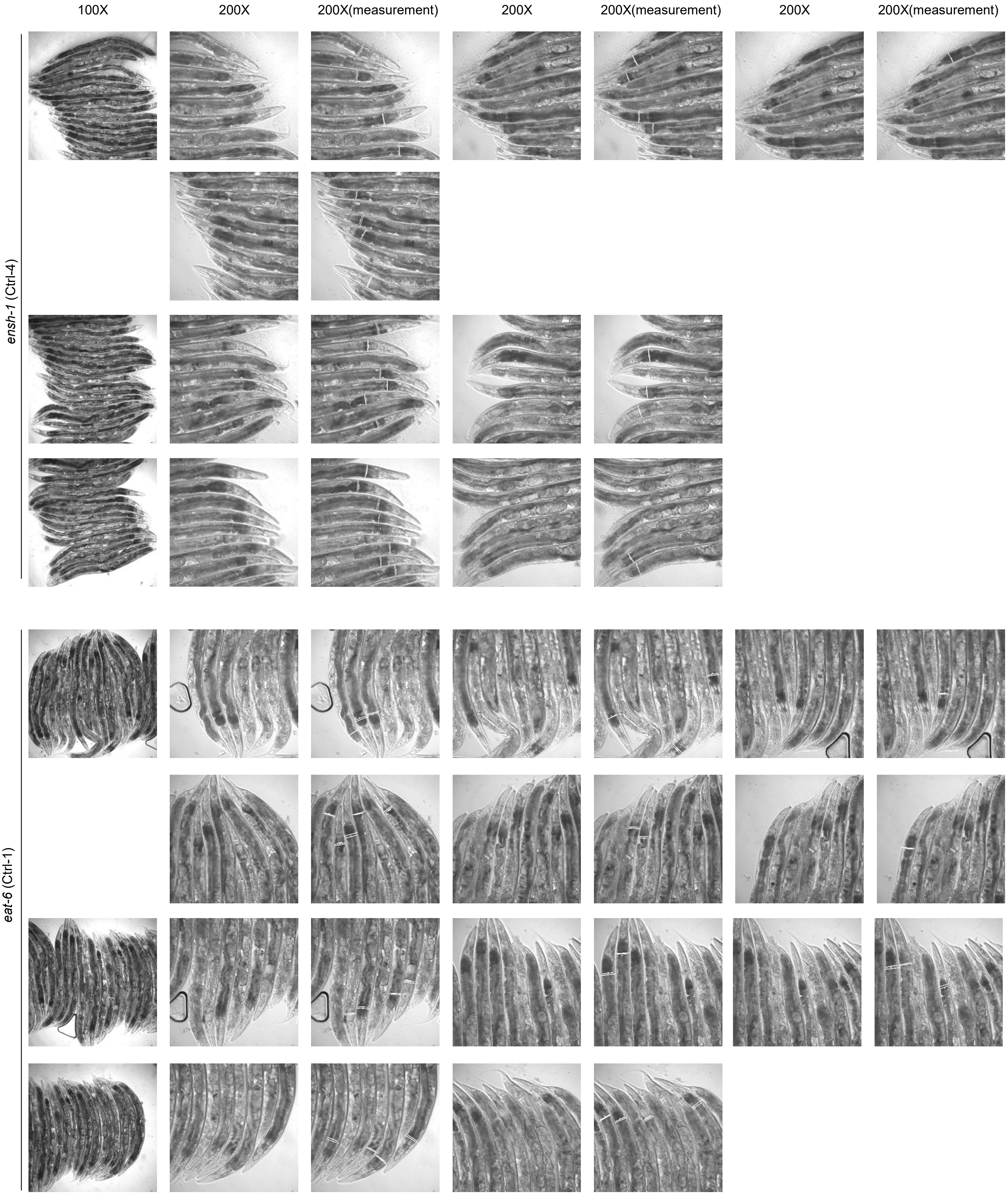

100X
200X
200X(measurement)
200X
200X(measurement)
200X
200X(measurement)
ensh-1 (Ctrl-4)
eat-6 (Ctrl-1)

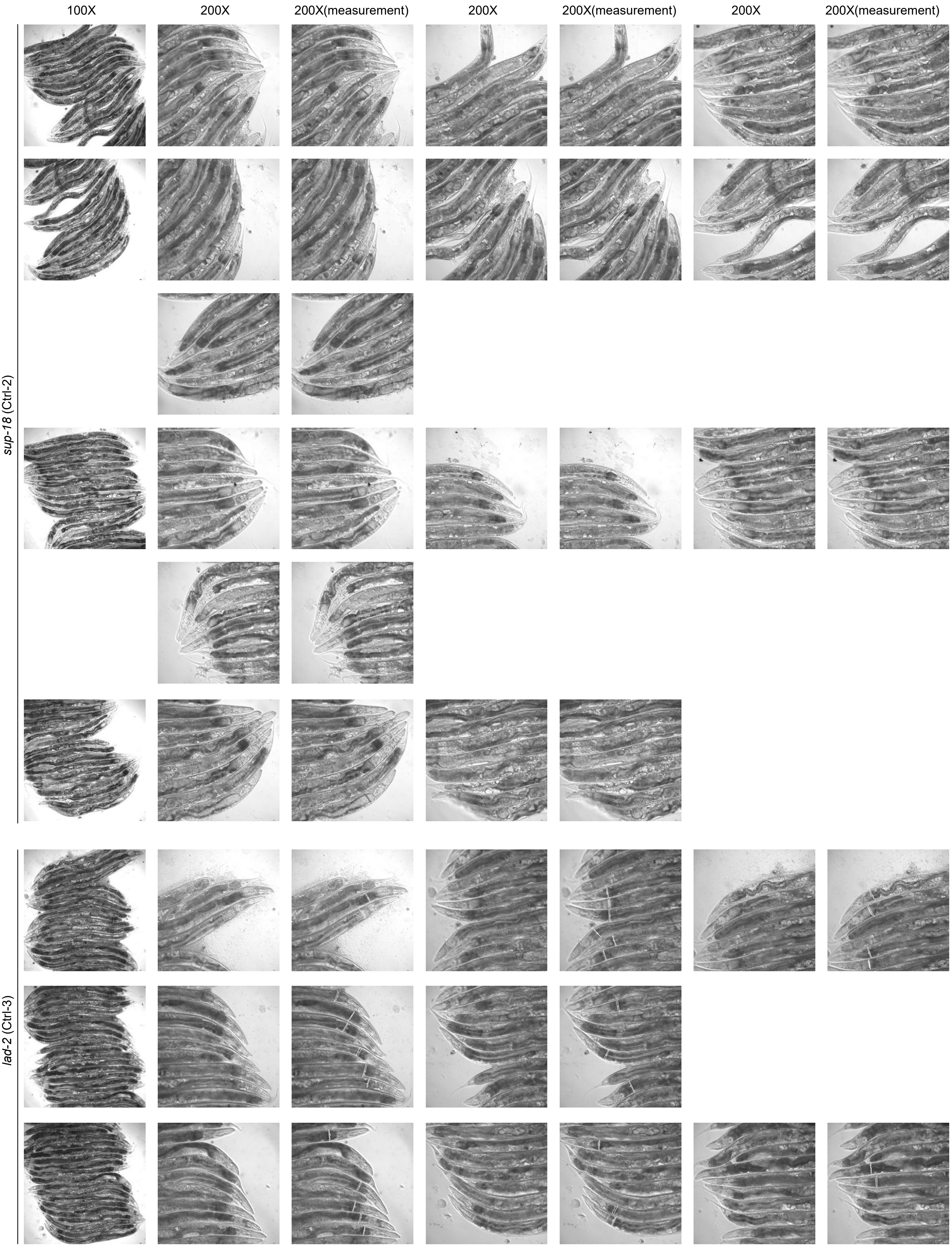
100X
200X
200X(measurement)
200X
200X(measurement)
200X
200X(measurement)
sup-18 (Ctrl-2)
lad-2 (Ctrl-3)

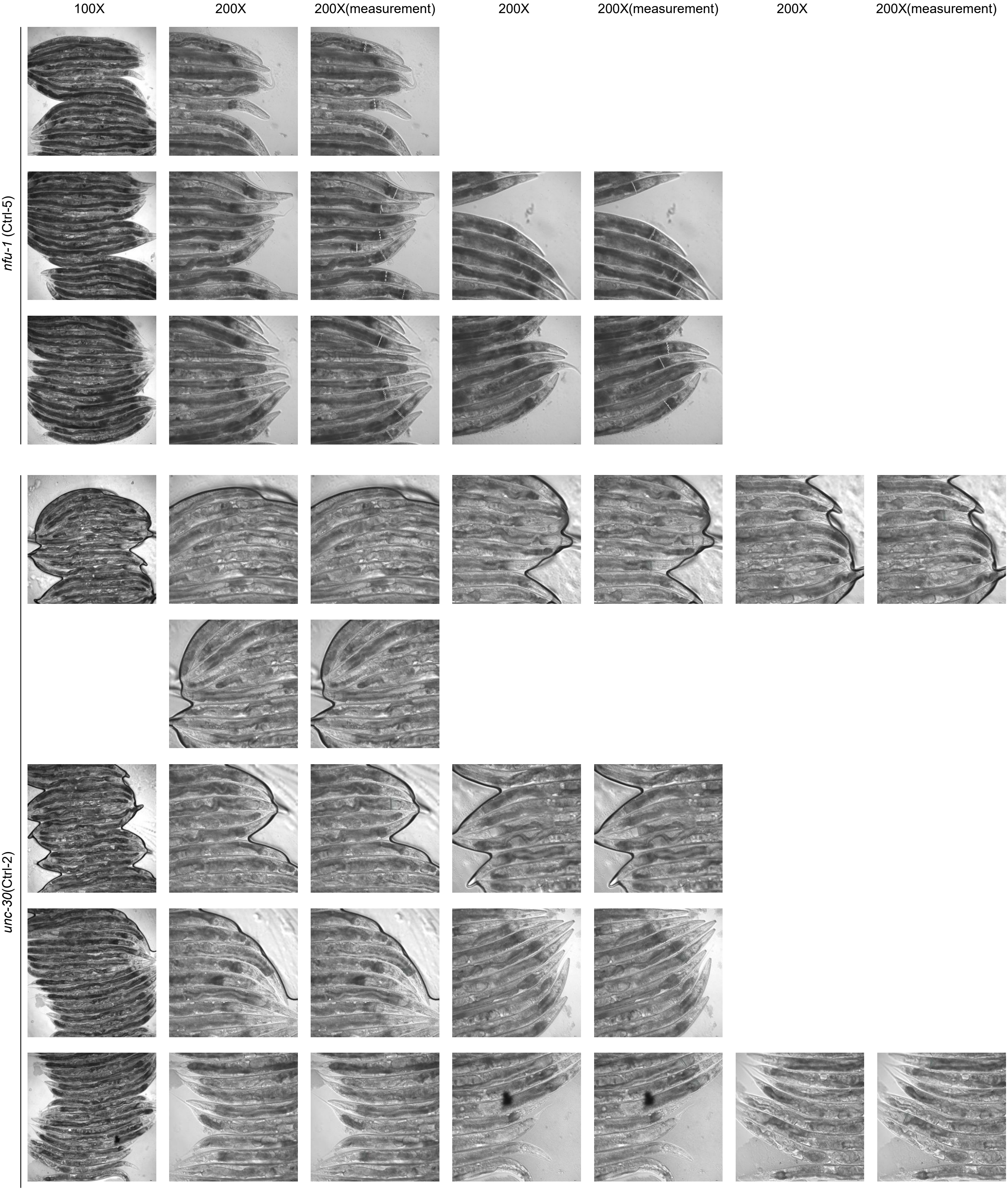
100X
200X
200X(measurement)
200X
200X(measurement)
200X
200X(measurement)
nfu-1 (Ctrl-5)
unc-30(Ctrl-2)

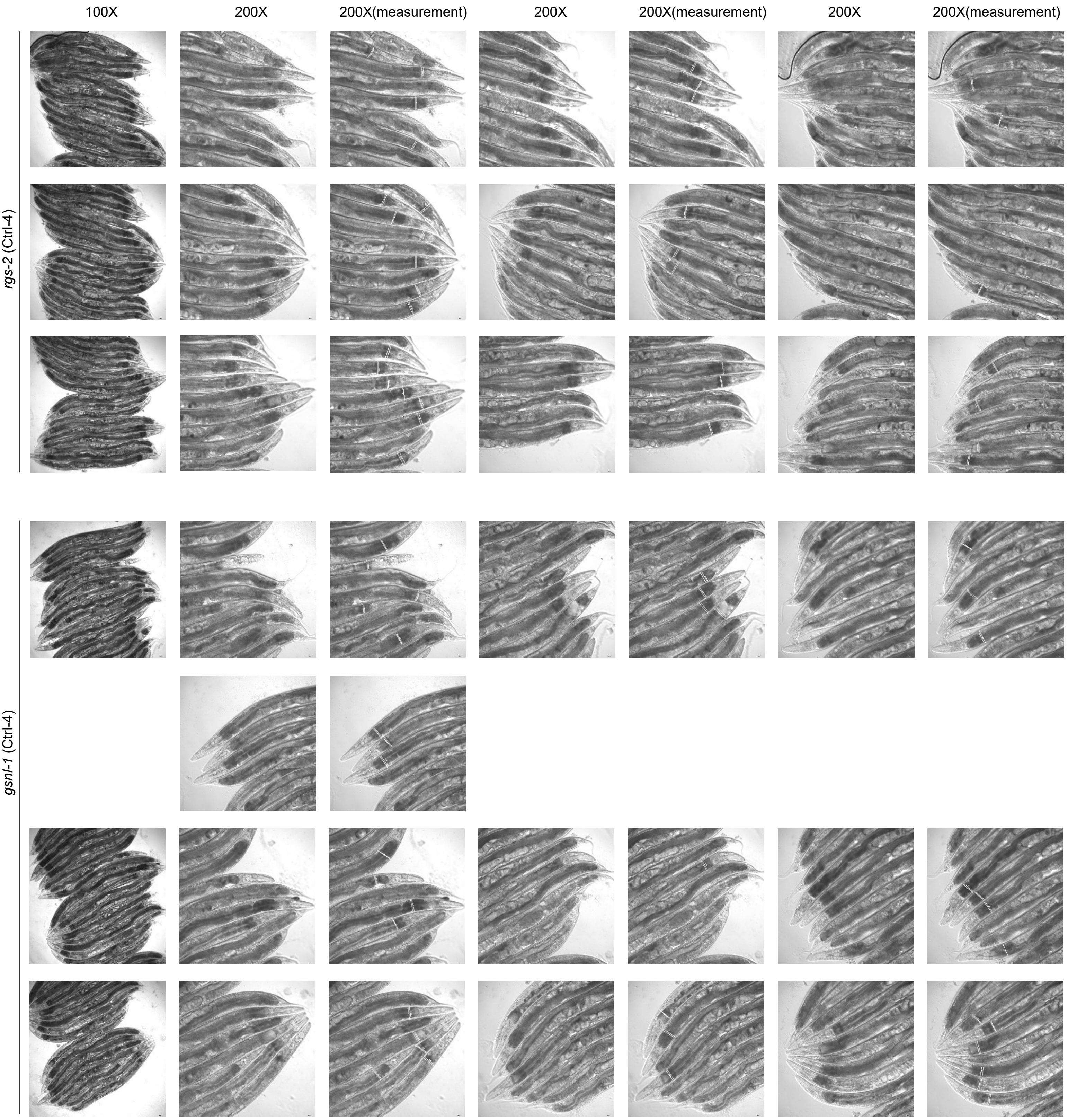
100X
200X
200X(measurement)
200X
200X(measurement)
200X
200X(measurement)
rgs-2 (Ctrl-4)
gsnl-1 (Ctrl-4)

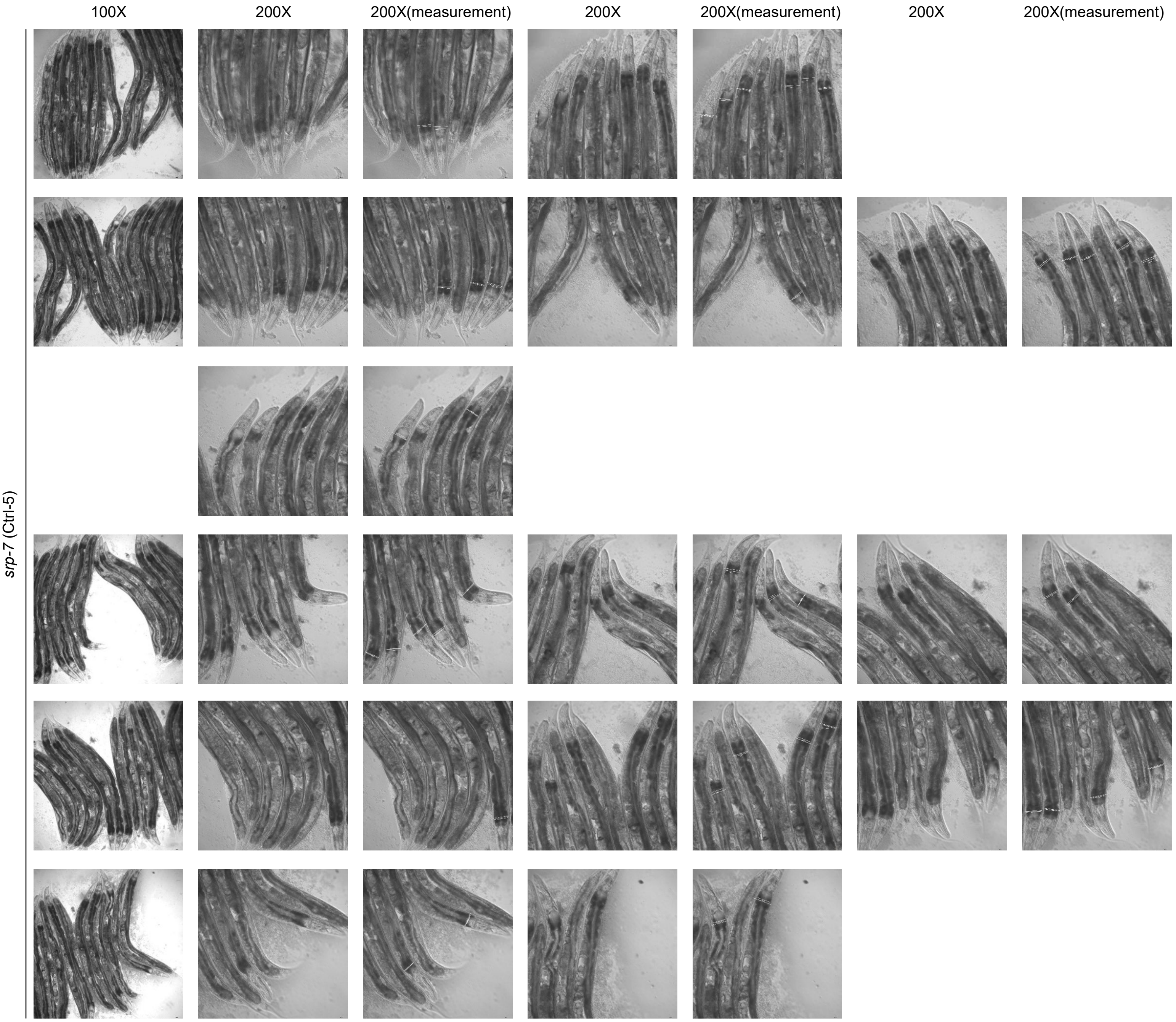
100X
200X
200X(measurement)
200X
200X(measurement)
200X
200X(measurement)
srp-7 (Ctrl-5)

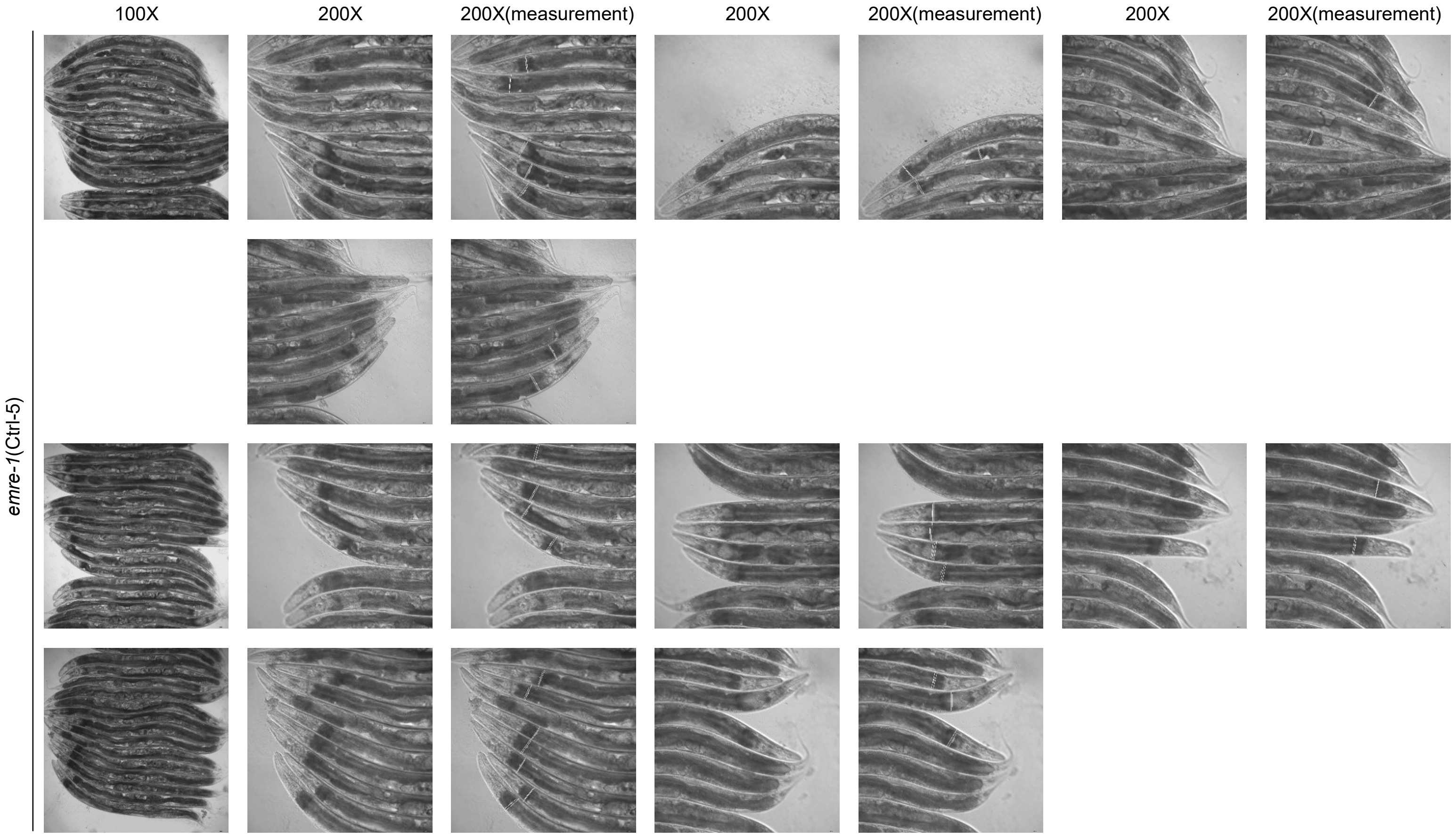
100X
200X
200X(measurement)
200X
200X(measurement)
200X
200X(measurement)
emre-1(Ctrl-5)

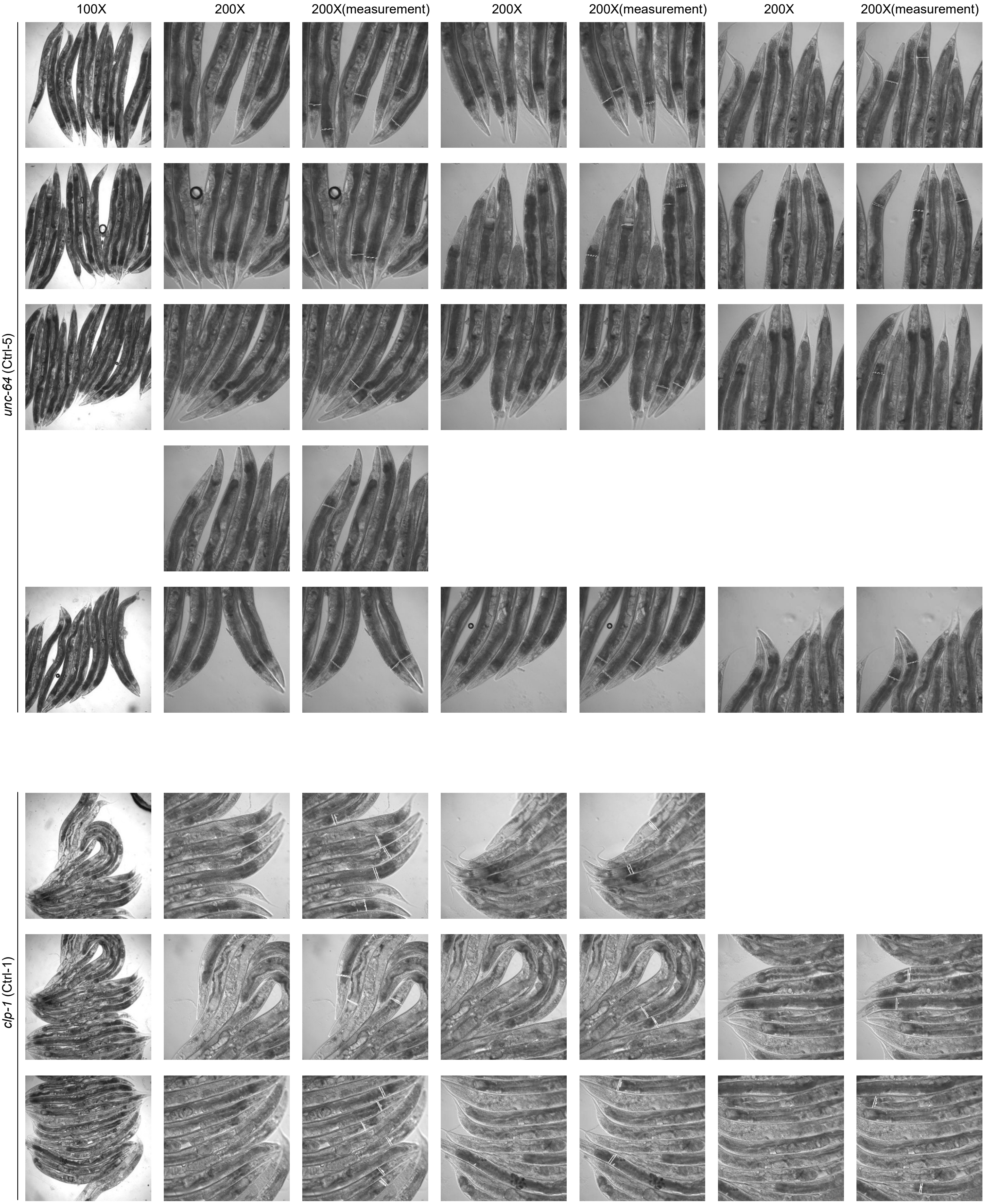
100X
200X
200X(measurement)
200X
200X(measurement)
200X
200X(measurement)
unc-64 (Ctrl-5)
clp-1 (Ctrl-1)

Fig. 4i

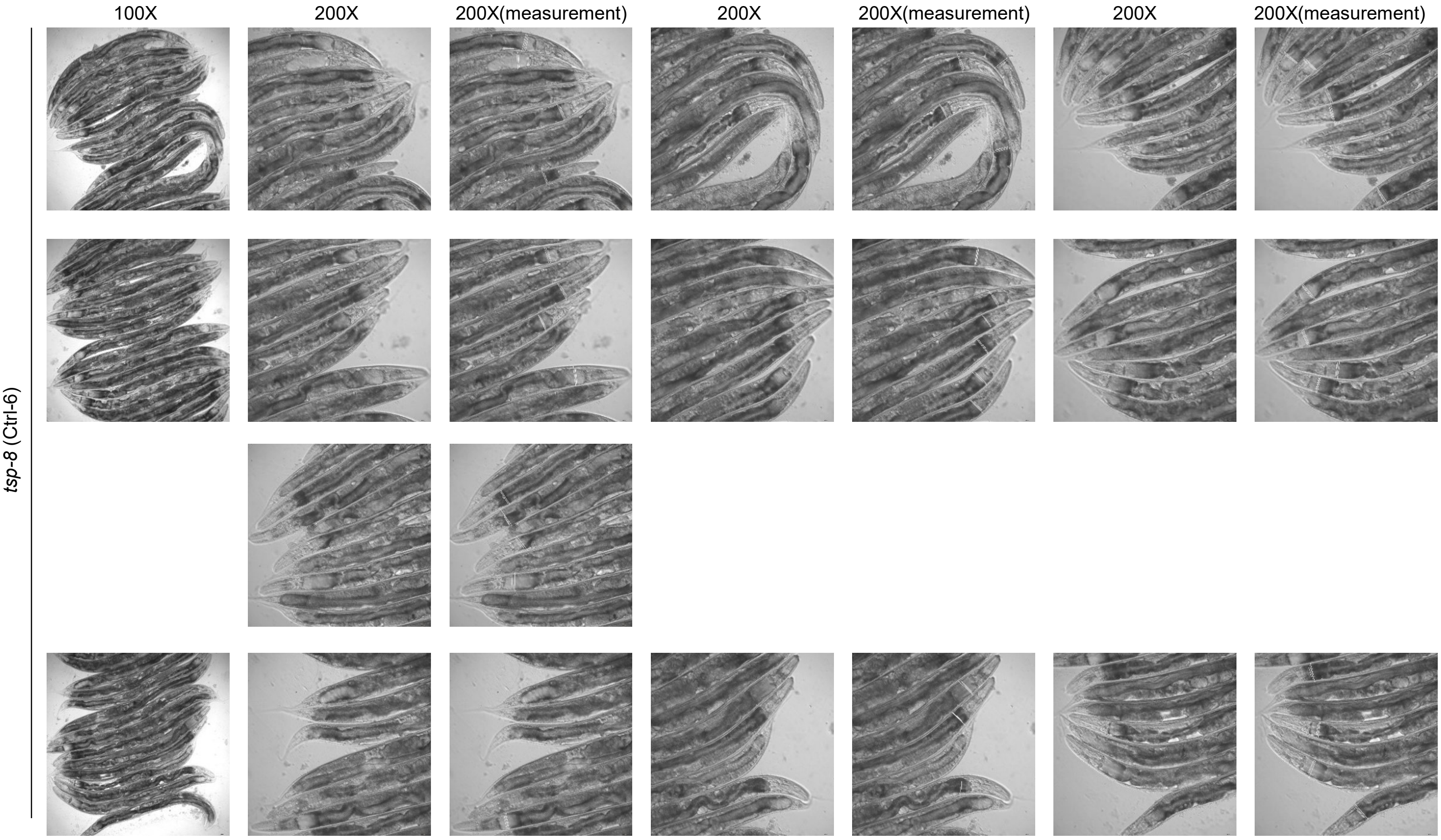
100X
200X
200X(measurement)
200X
200X(measurement)
200X
200X(measurement)
tsp-8 (Ctrl-6)
