# Supplementary figures and images for: Age-, sex- and proximal–distal-resolved multi-omics identifies regulators of intestinal aging in non-human primates
Source: Nat Aging. 2024 Feb 6;4(3):414–33. doi: 10.1038/s43587-024-00572-9 (PMC10950786; doi:10.1038/s43587-024-00572-9)

Fig. 5b

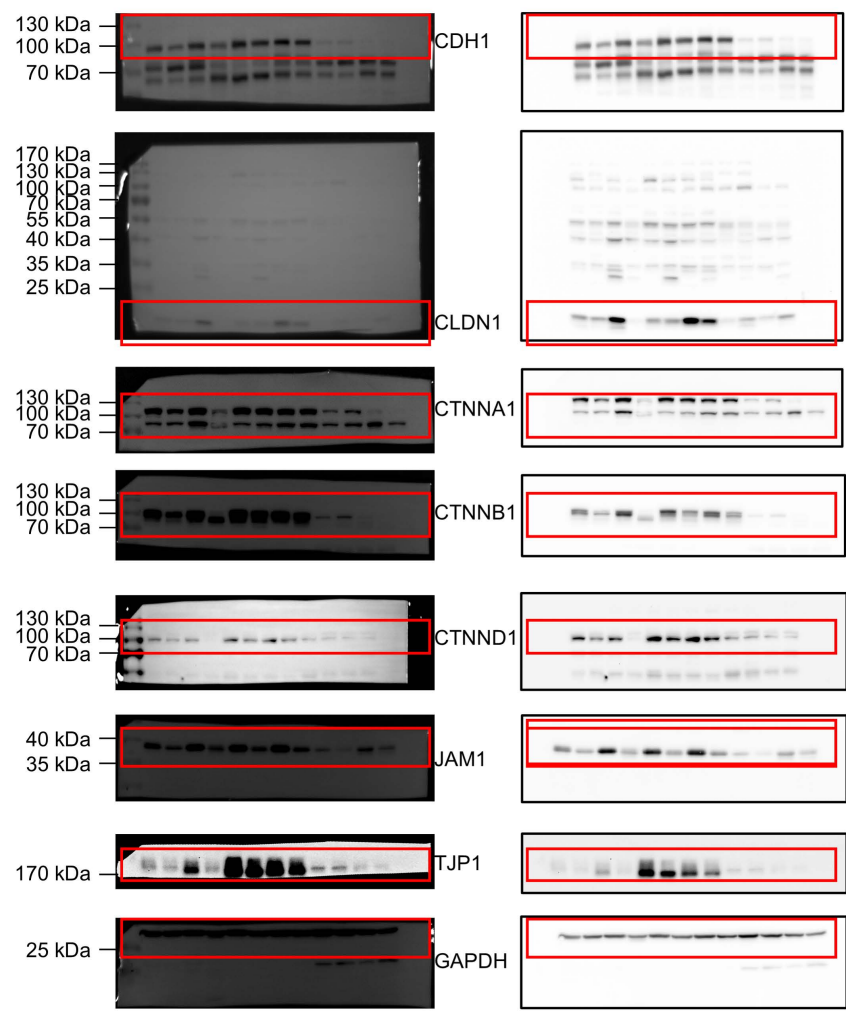

Fig. 5d

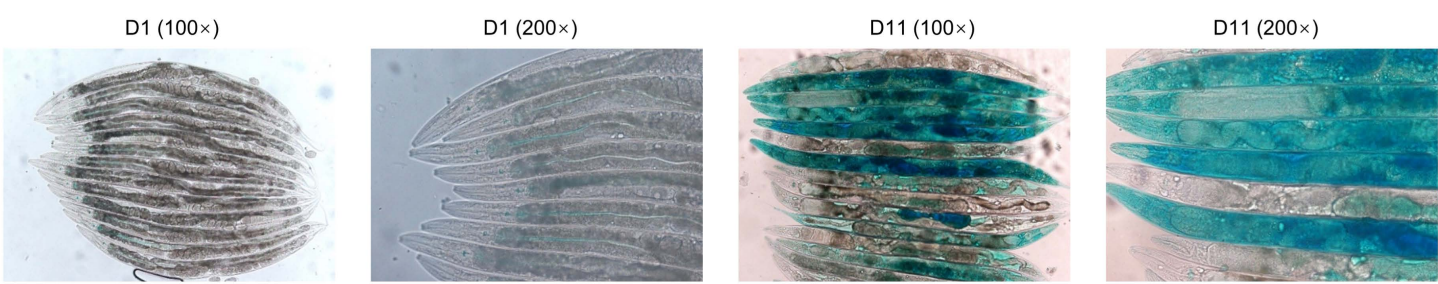

Fig. 5e

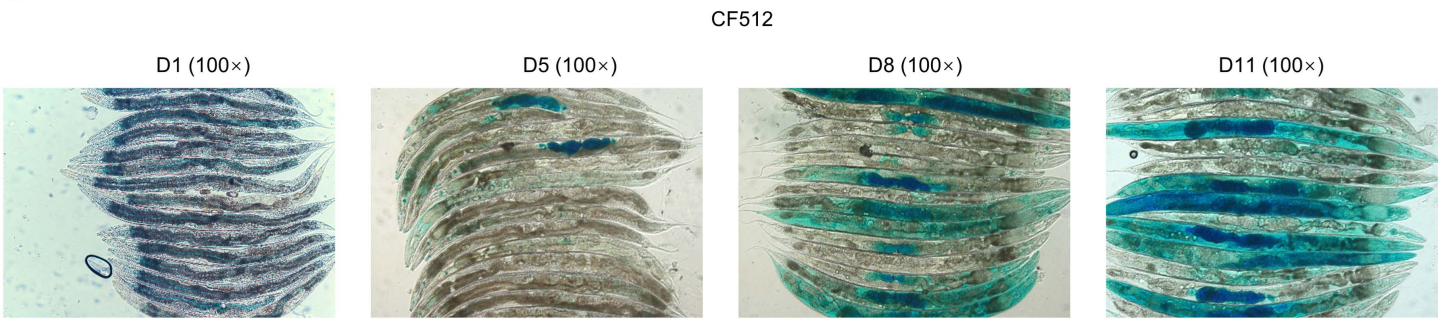

Supplement: Supplementary file 19 — Unprocessed western blots and original images. [file 43587_2024_572_MOESM19_ESM.pdf]

Fig. 6h,i

Replicate 1

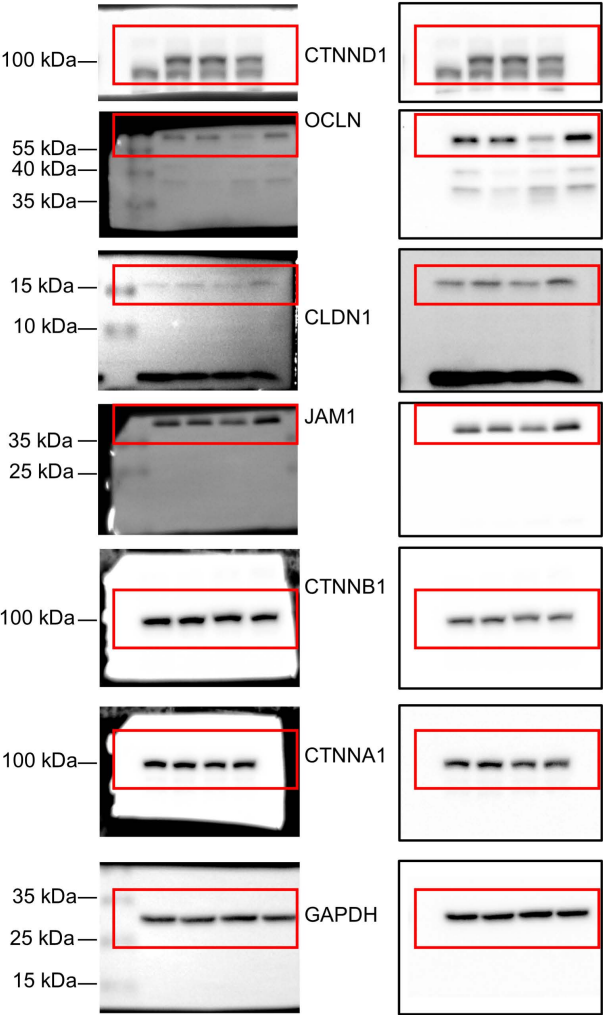

Replicate 2

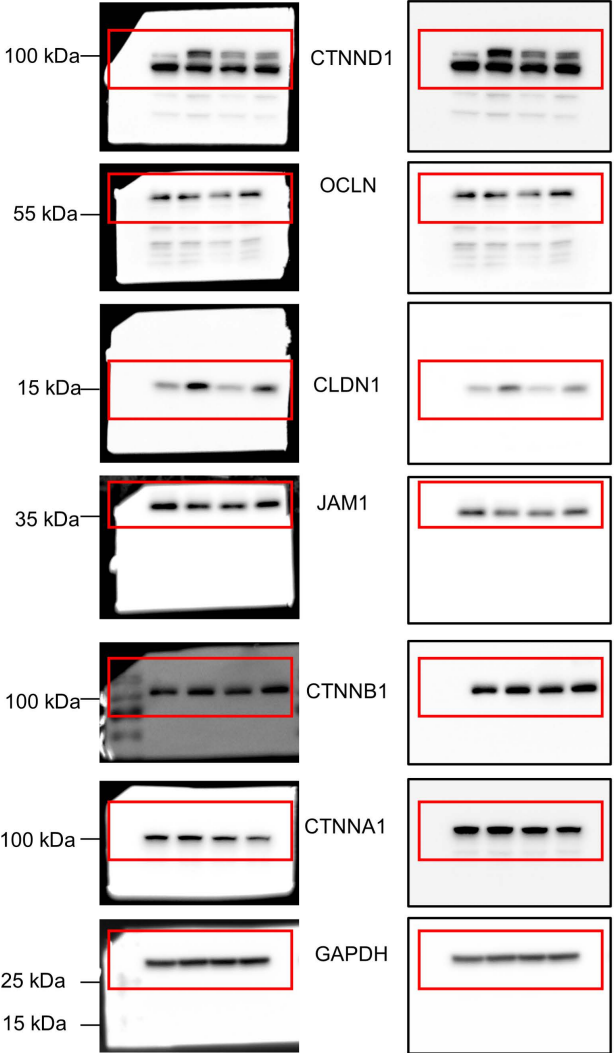

Replicate 3

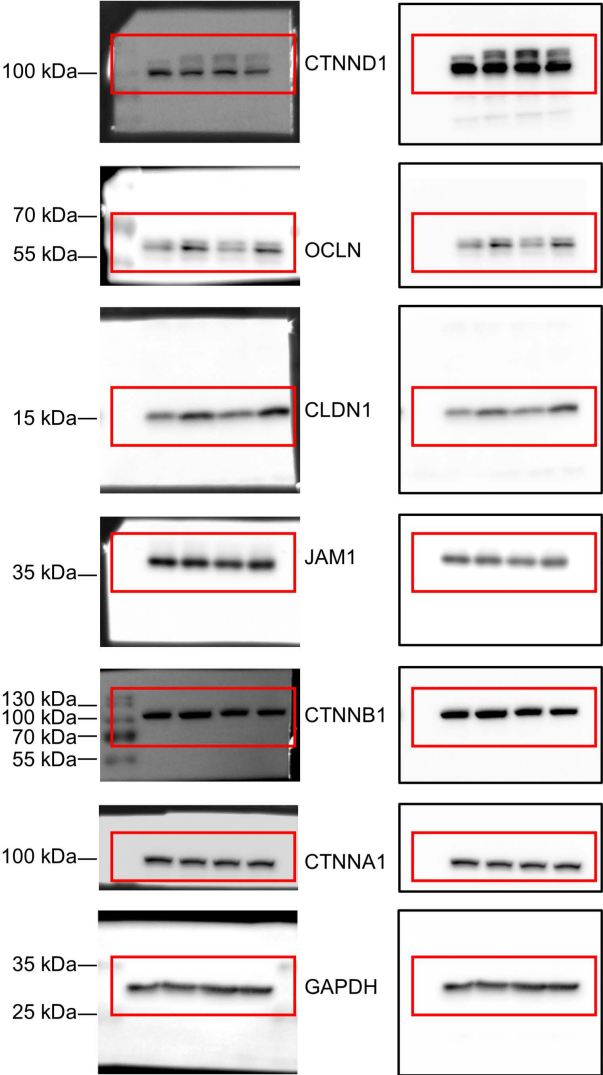

Supplement: Supplementary file 20 — Unprocessed western blots and original images. [file 43587_2024_572_MOESM20_ESM.pdf]
