# Supplementary material for: Age-, sex- and proximal–distal-resolved multi-omics identifies regulators of intestinal aging in non-human primates
Source: Nat Aging. 2024 Feb 6;4(3):414–33. doi: 10.1038/s43587-024-00572-9 (PMC10950786; doi:10.1038/s43587-024-00572-9)

Fig. 7b

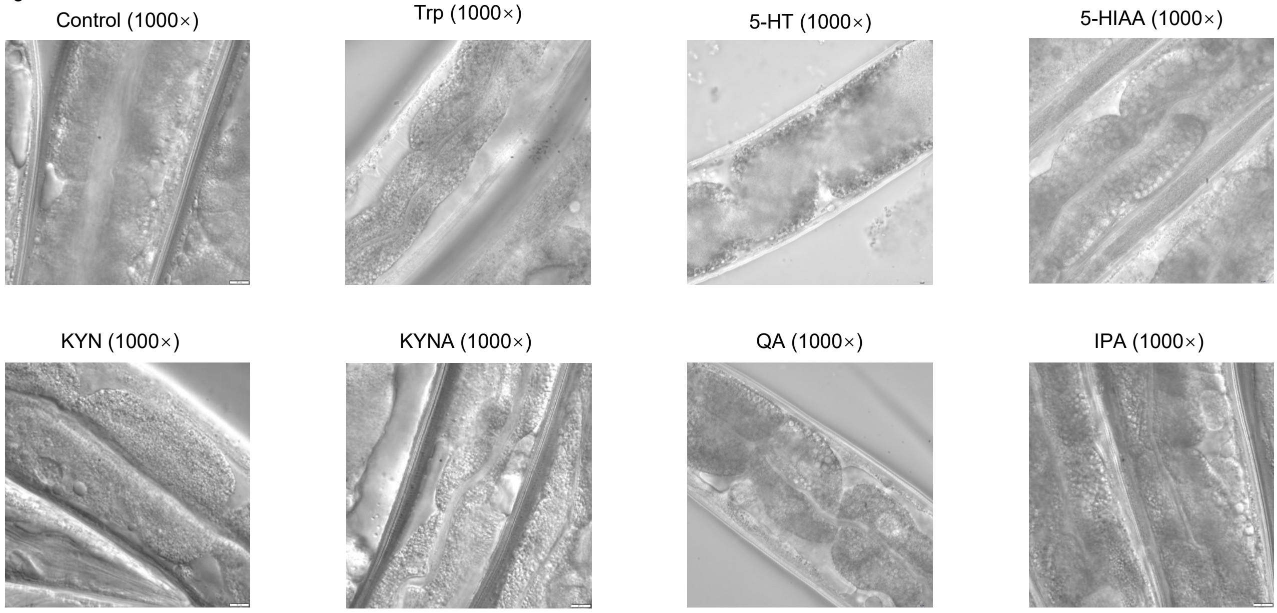
Control (1000×)
Trp (1000×)
5-HT (1000×)
5-HIAA (1000×)
KYN (1000×)
KYNA (1000×)
QA (1000×)
IPA (1000×)

Fig. 7c

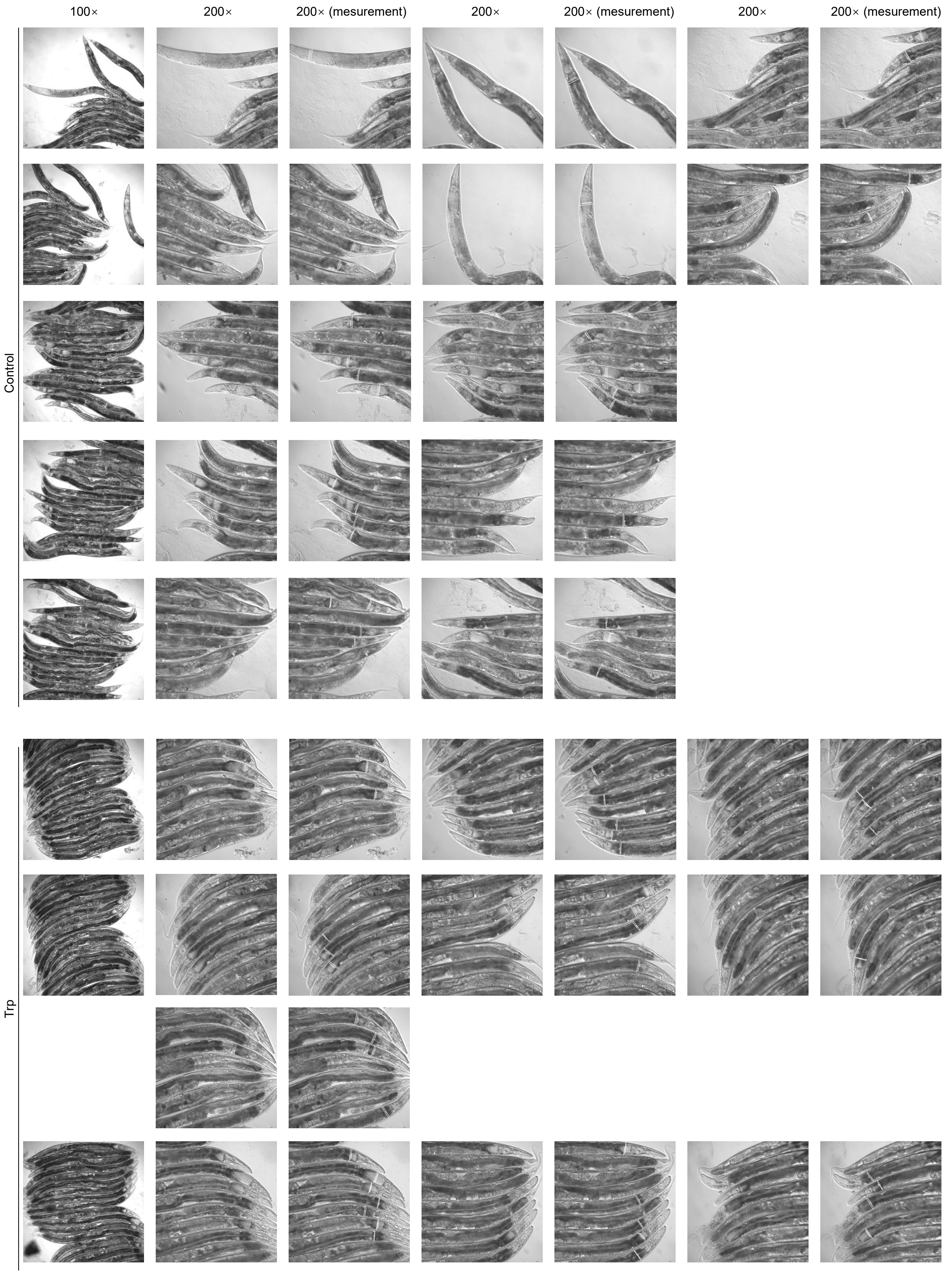
100×
200×
200× (mesurement)
200×
200× (mesurement)
200×
200× (mesurement)
Control
Trp

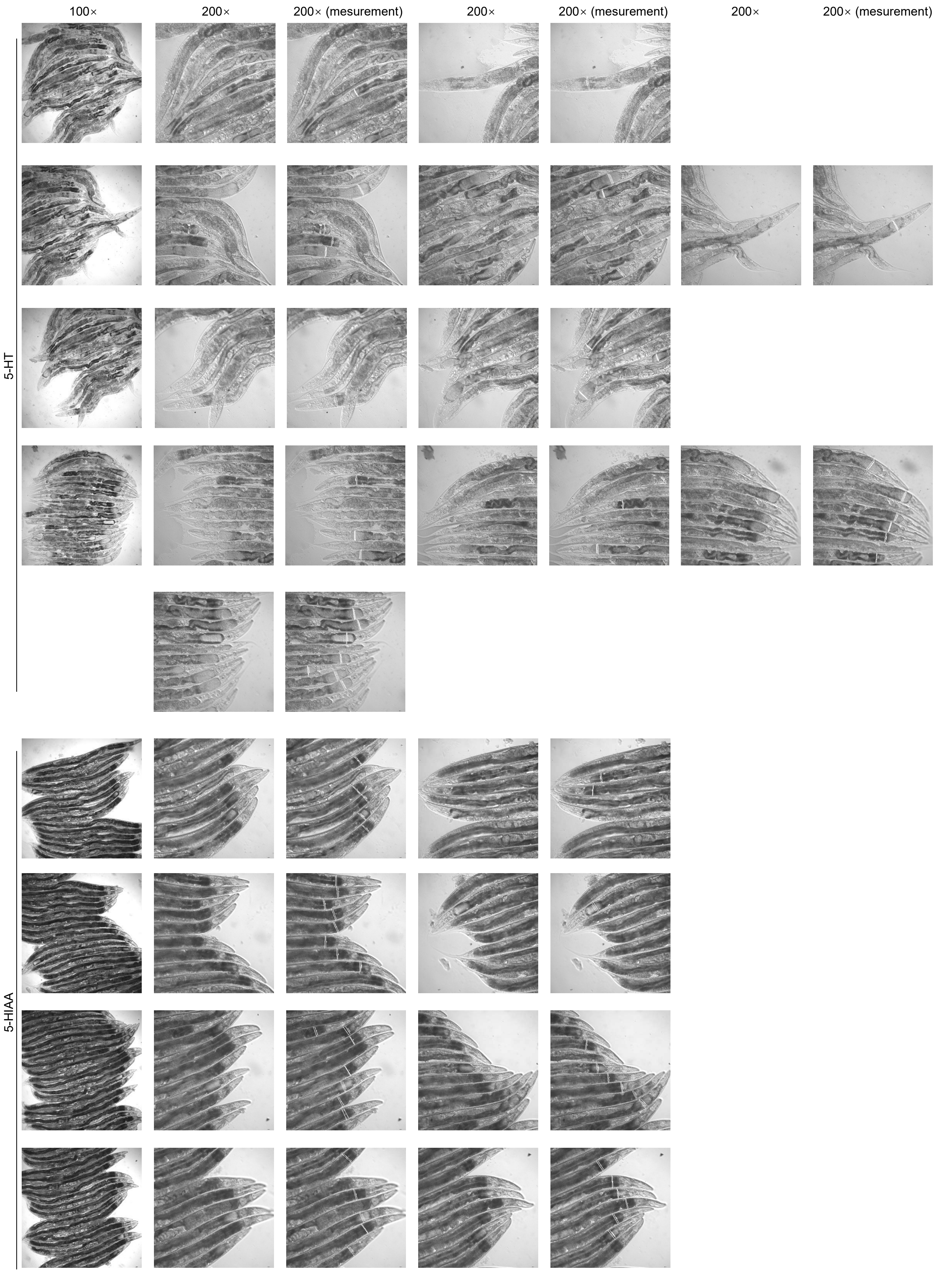

100×
200×
200× (mesurement)
200×
200× (mesurement)
200×
200× (mesurement)
5-HT
5-HIAA

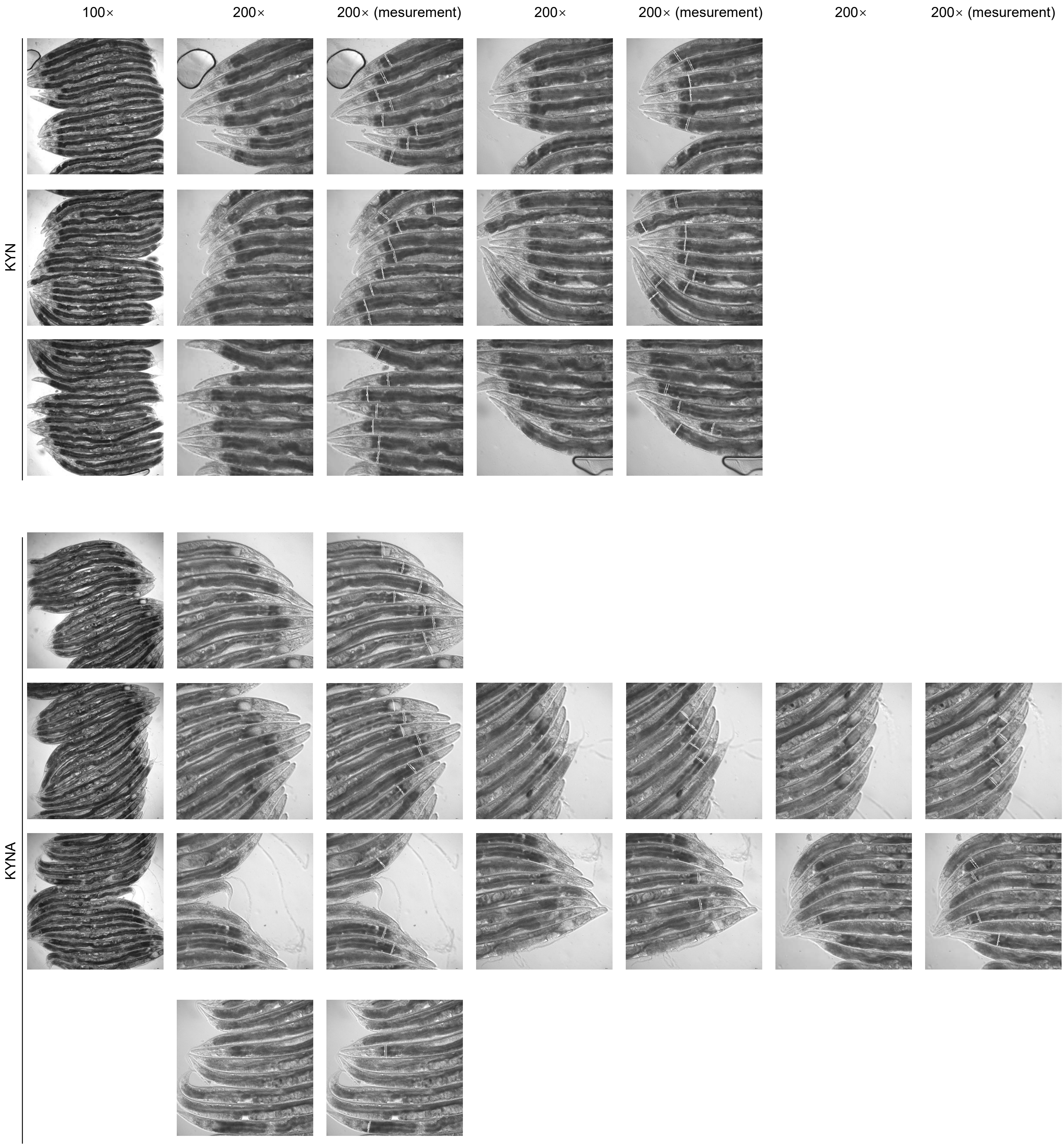
100×
200×
200× (mesurement)
200×
200× (mesurement)
200×
200× (mesurement)
KYN
KYNA

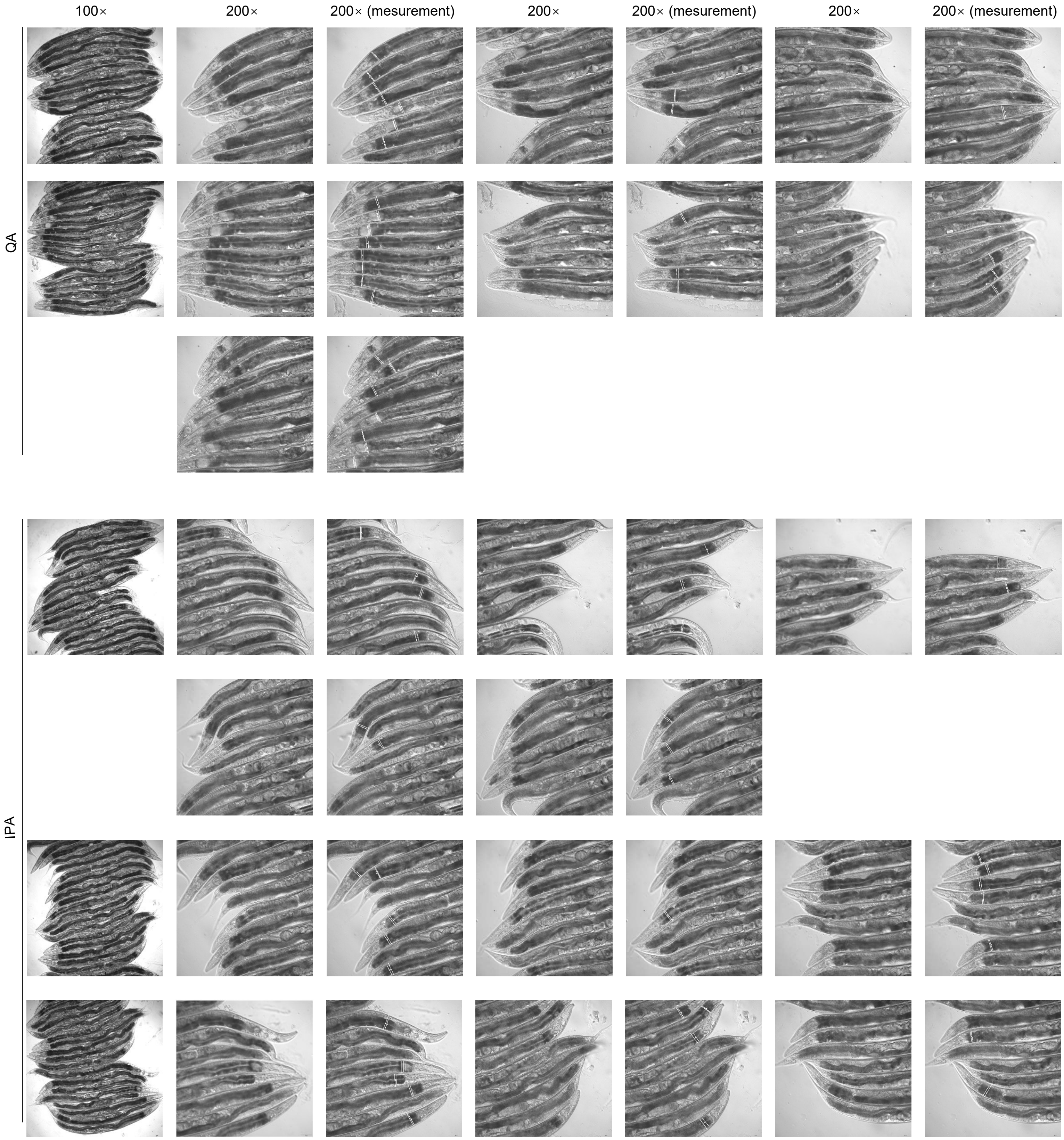
100×
200×
200× (mesurement)
200×
200× (mesurement)
200×
200× (mesurement)
QA
IPA

Fig. 7e

The red arrows indicate the intestinal nucleus used for measuring fluorescence intensity.
Control -1 (for Trp & 5-HIAA) (1000×)

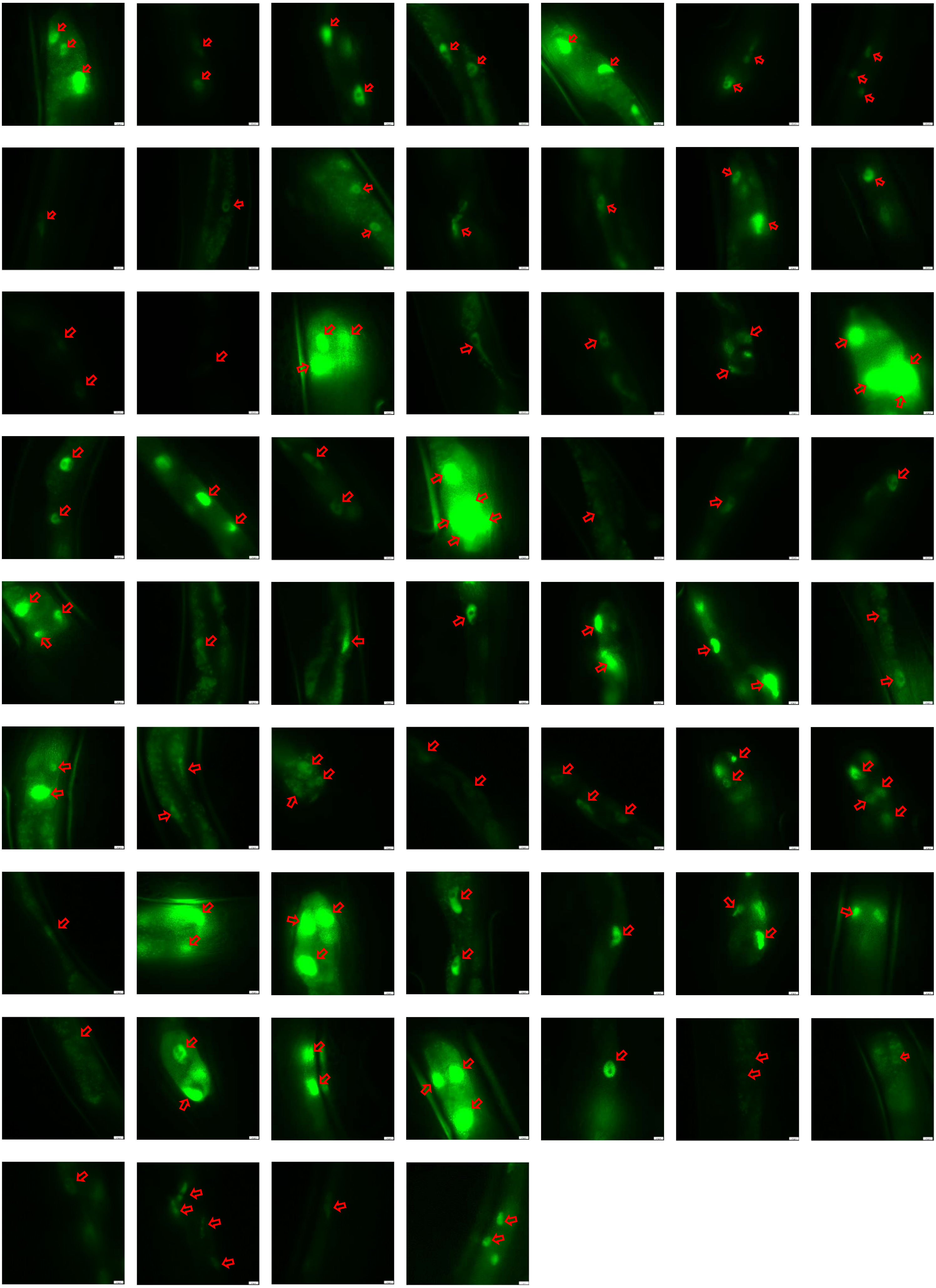

Control-2 (for 5-HT) (1000×)

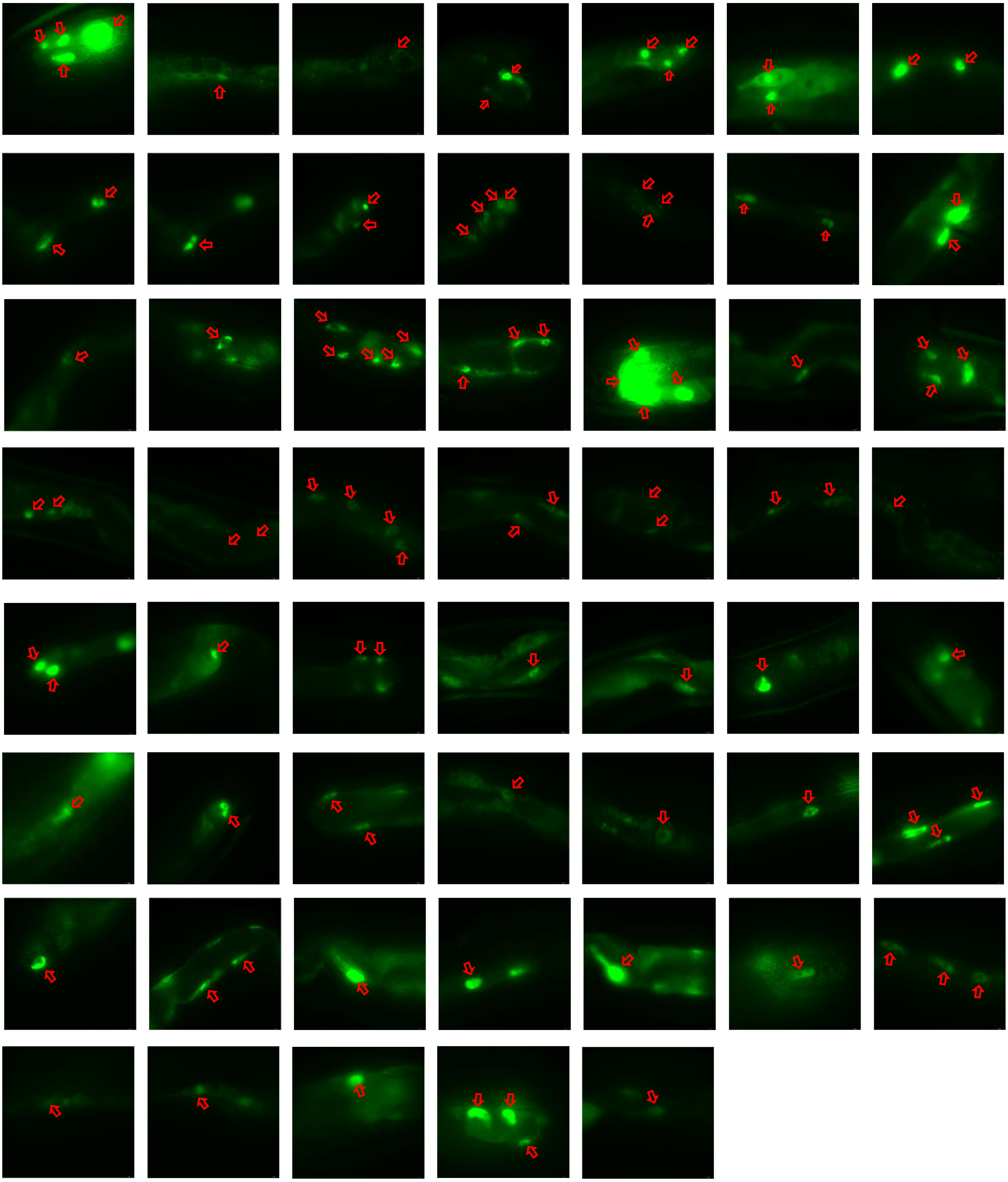

Control-3 (for KYN & IPA) (1000×)

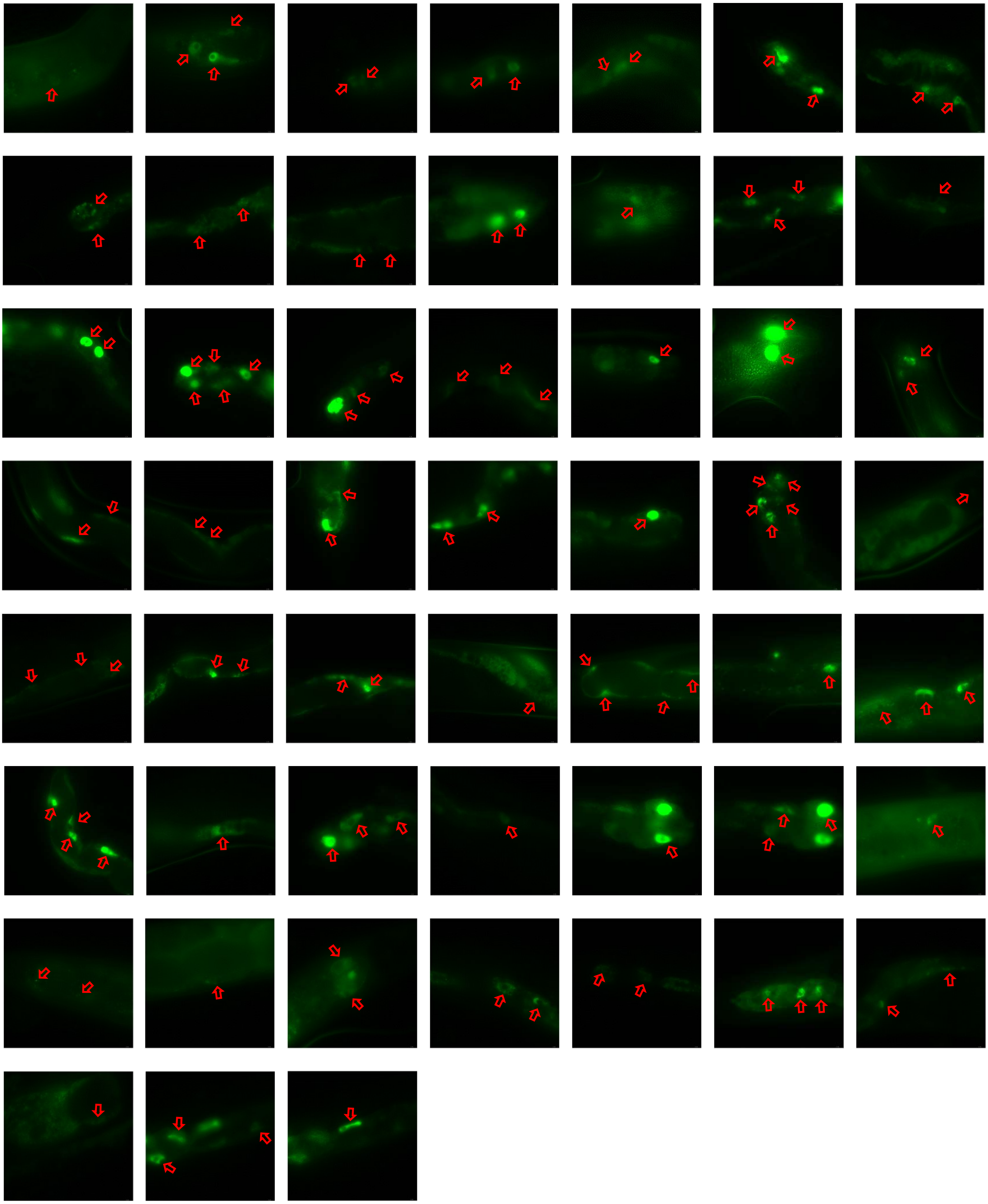

Control-4 (for KYNA) (1000×)

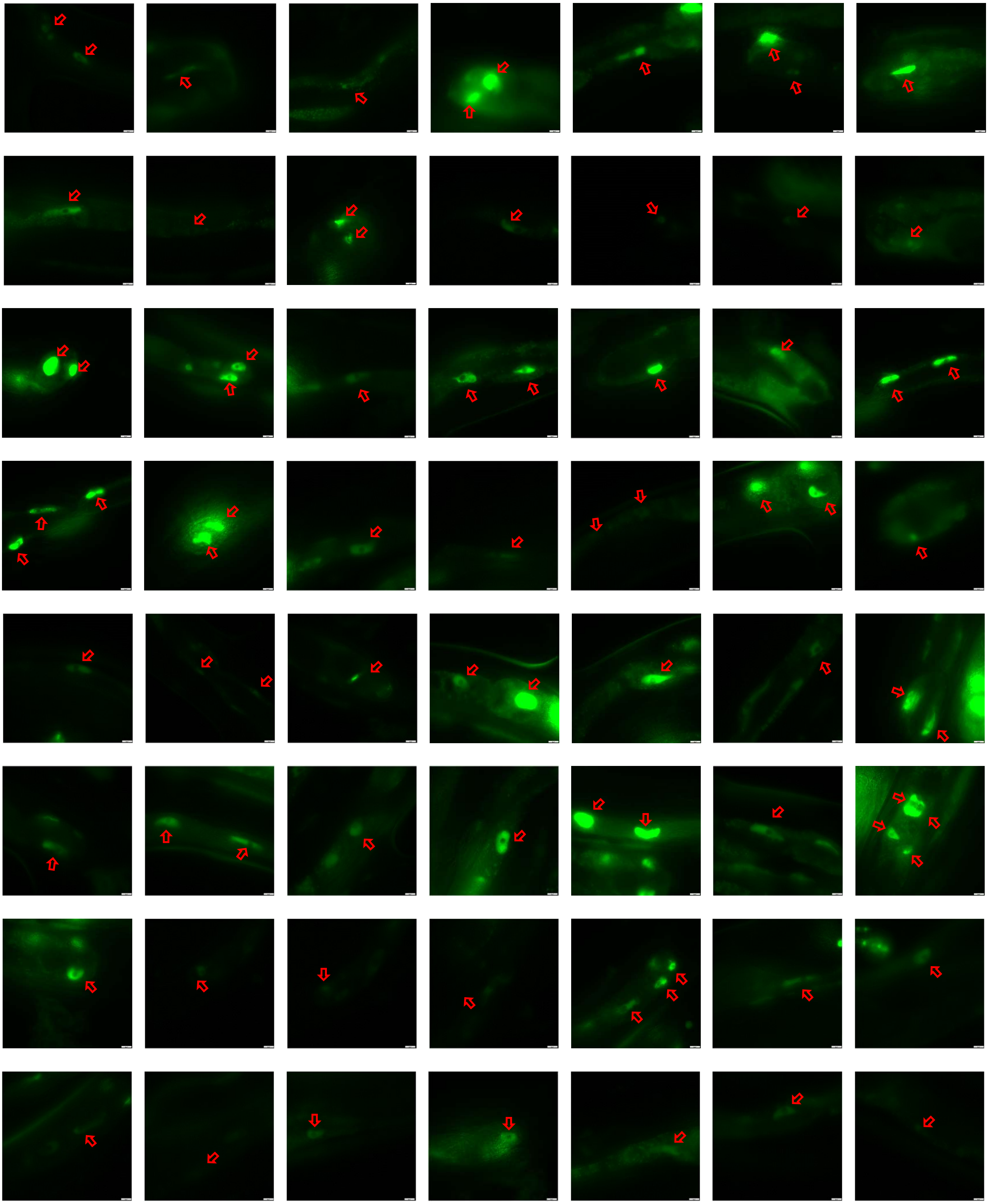

Control -5 (for QA)(1000×)

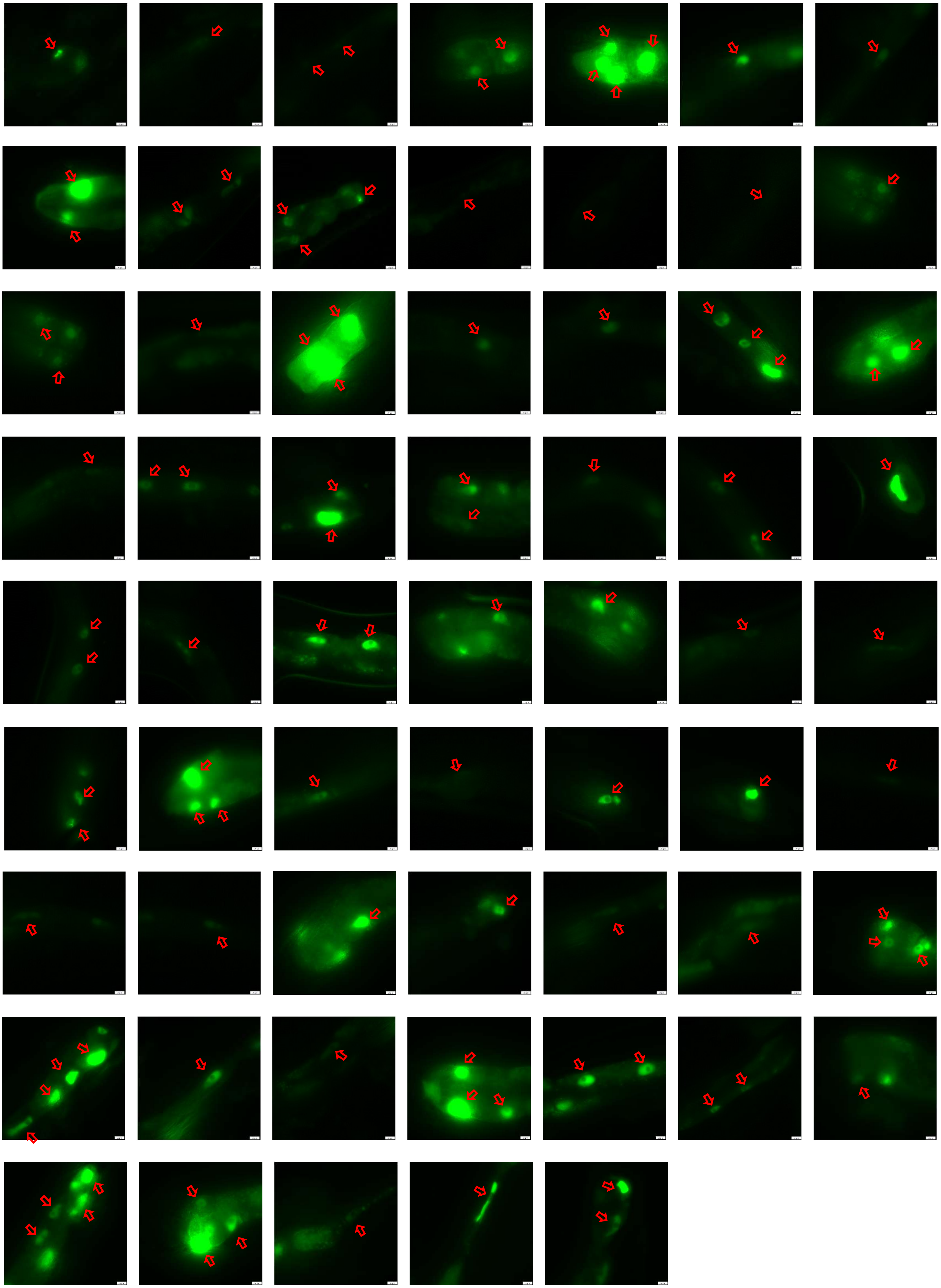

Trp-Metabolites (Control-1) (1000×)

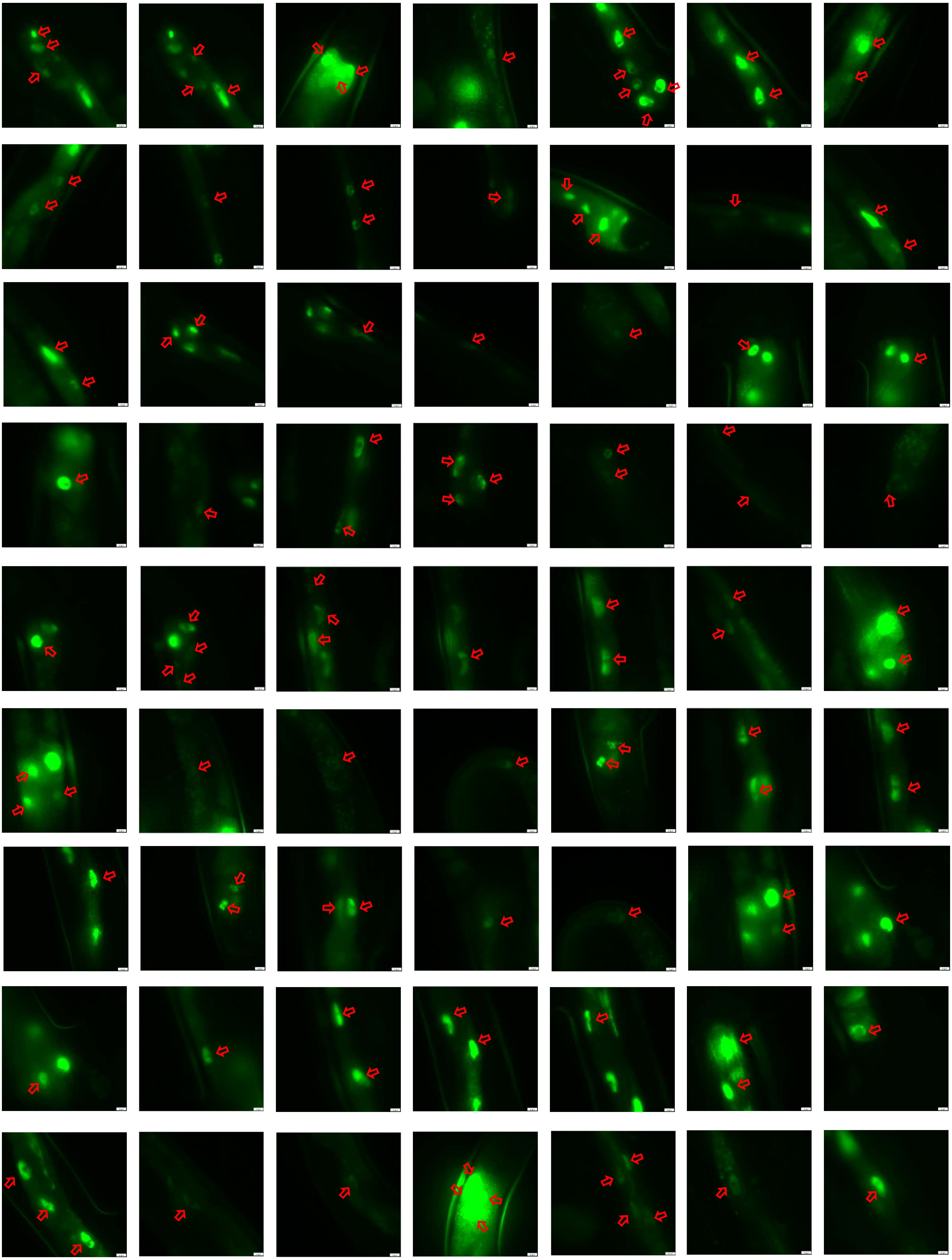

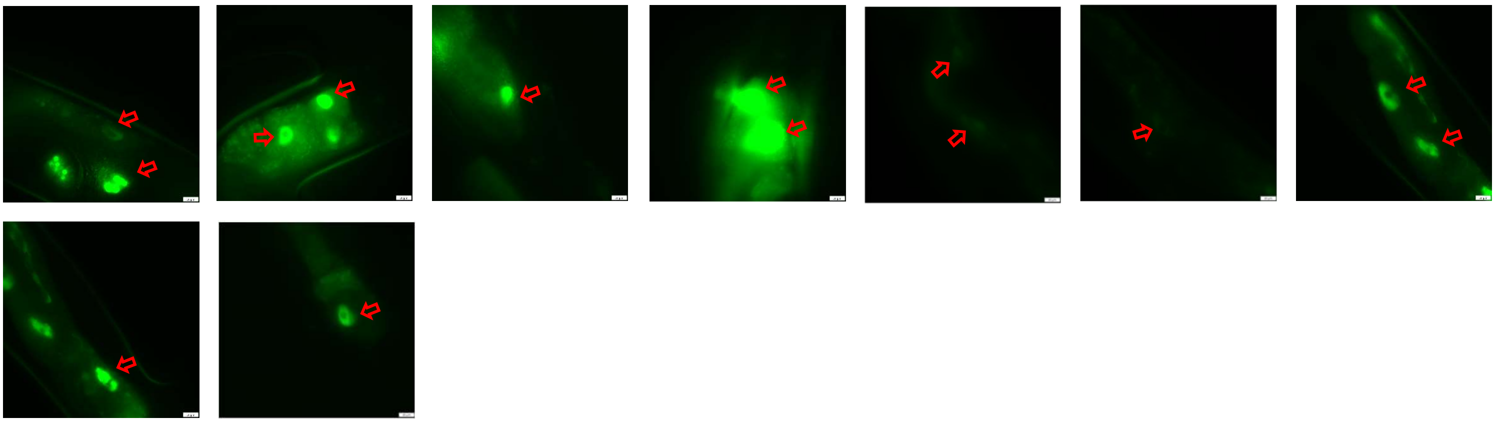

5-HIAA-Metabolites (Control-1) (1000×)

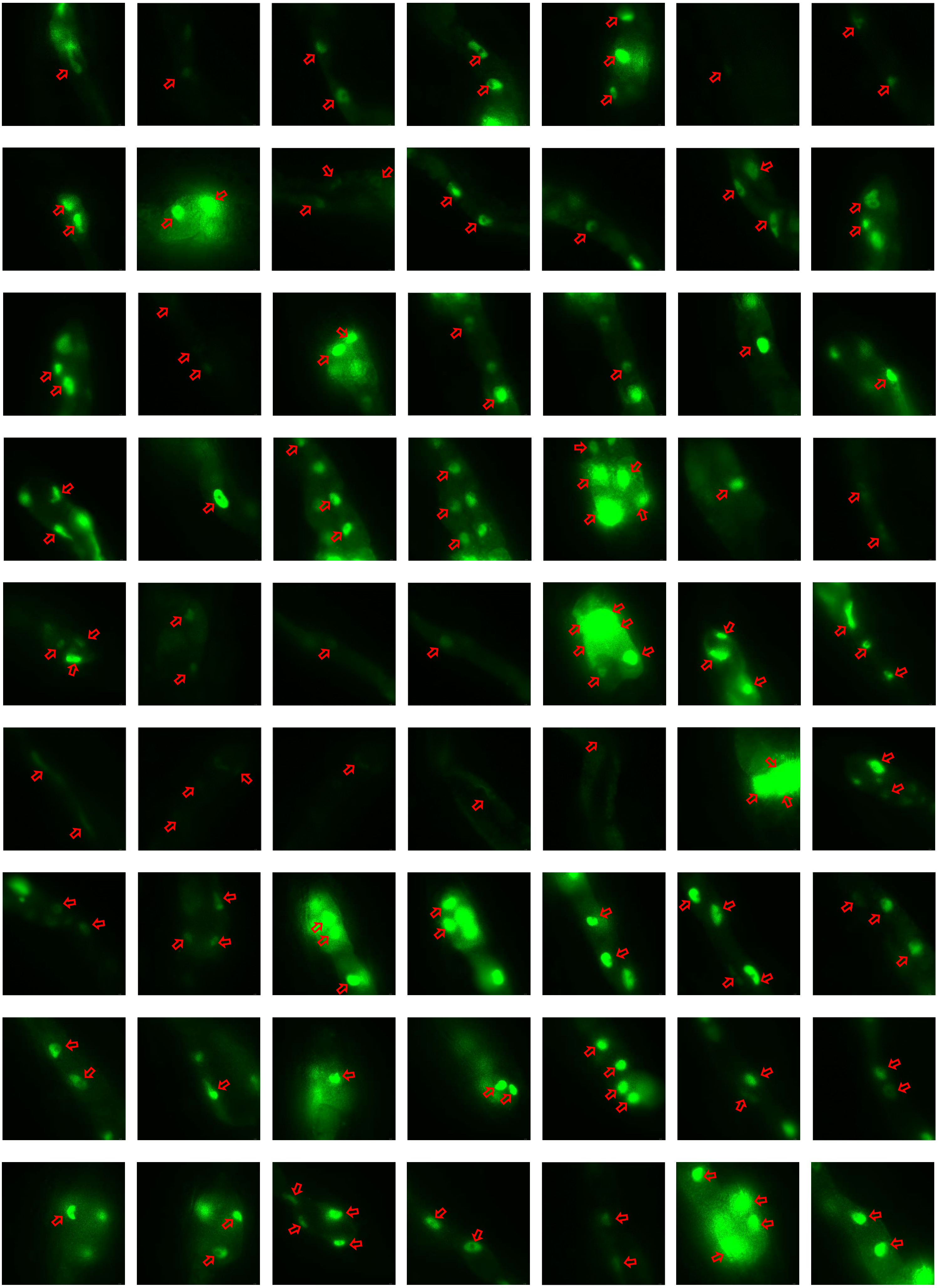

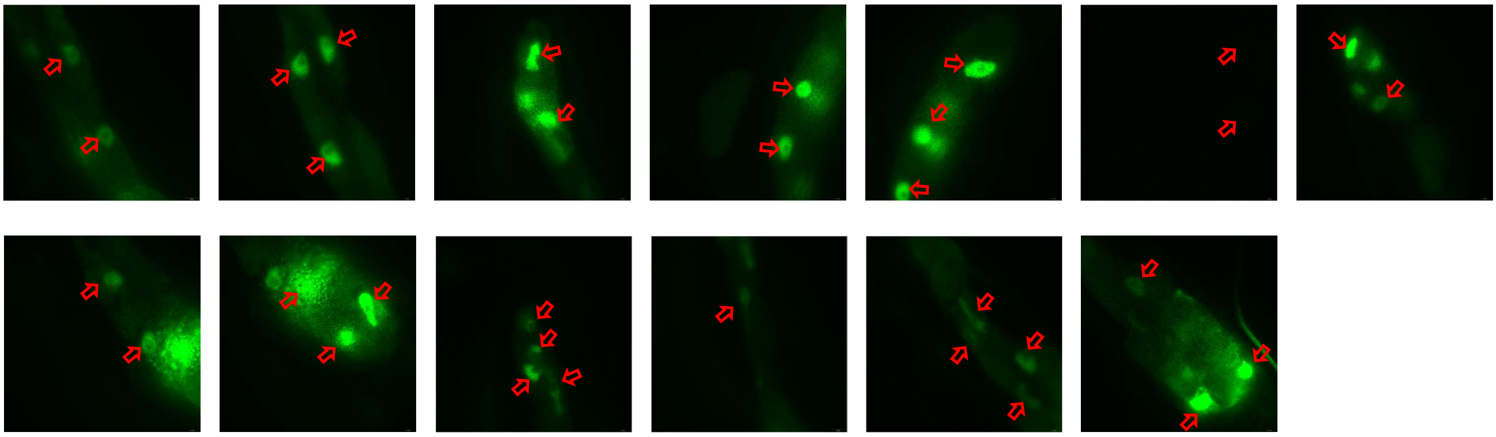

5-HT-Metabolites (Control-2) (1000×)

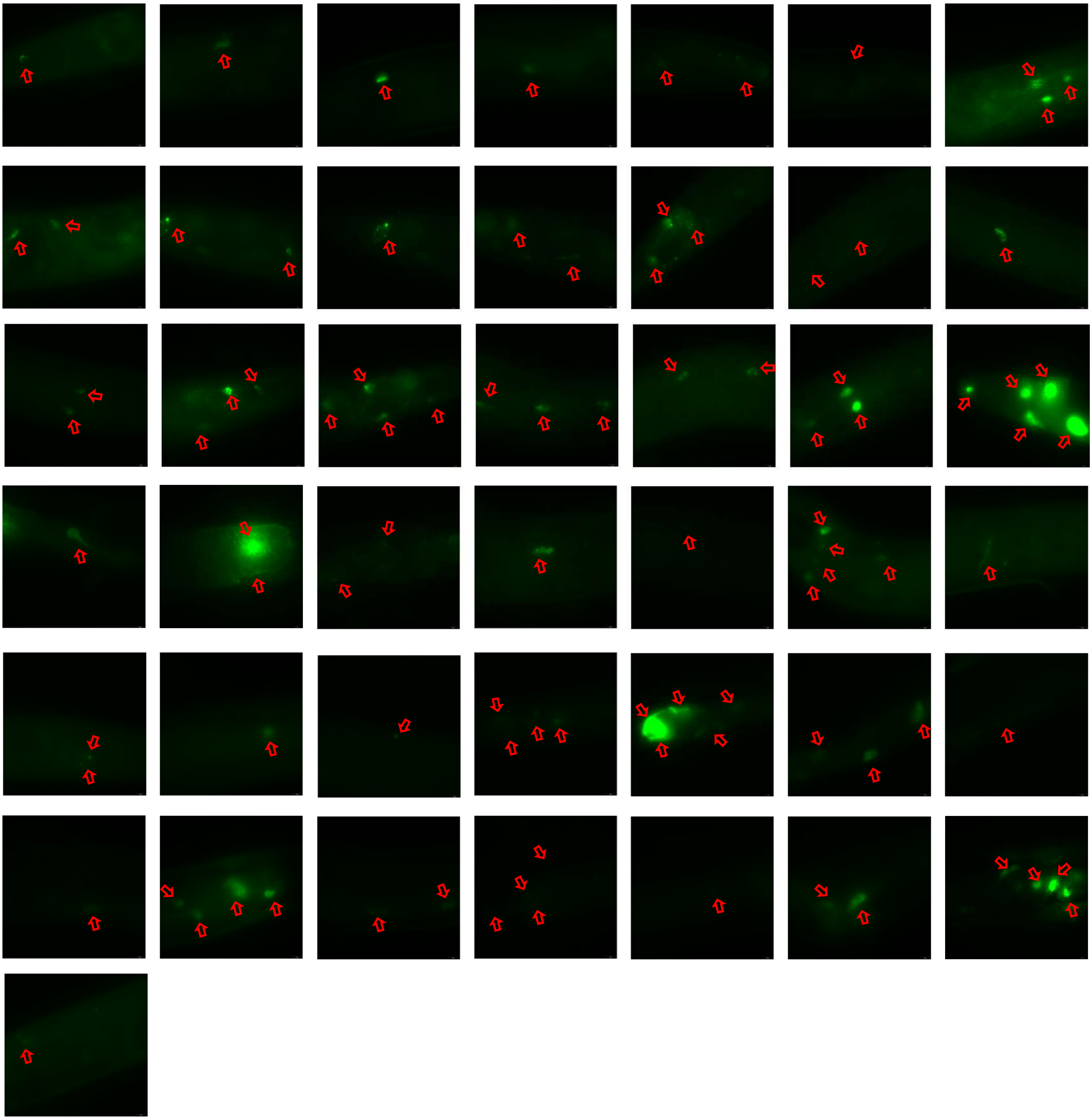

KYN-Metabolites (Control-3) (1000×)

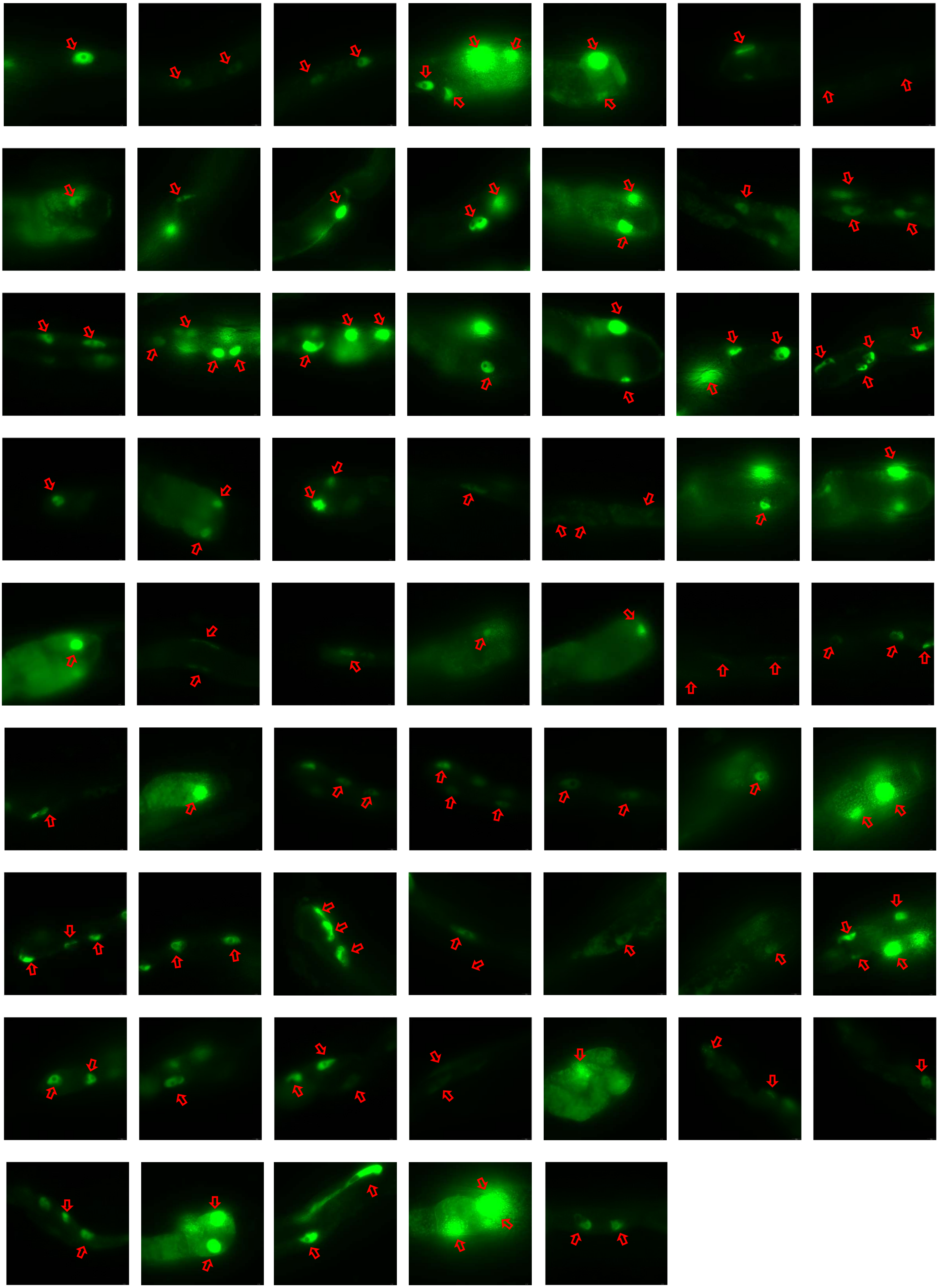

IPA-Metabolites (Control-3)(1000×)

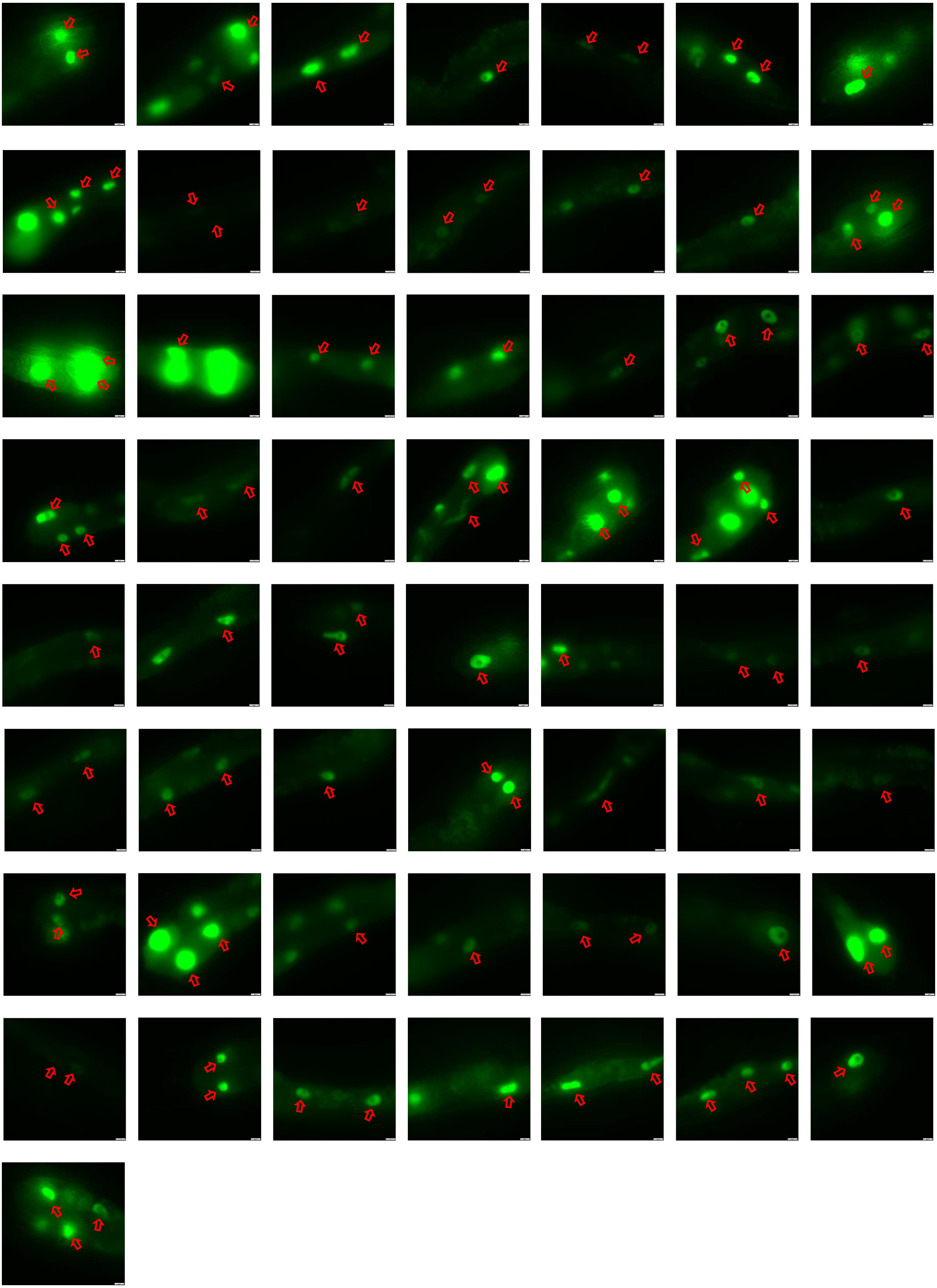

KYNA-Metabolites (Control-4) (1000×)

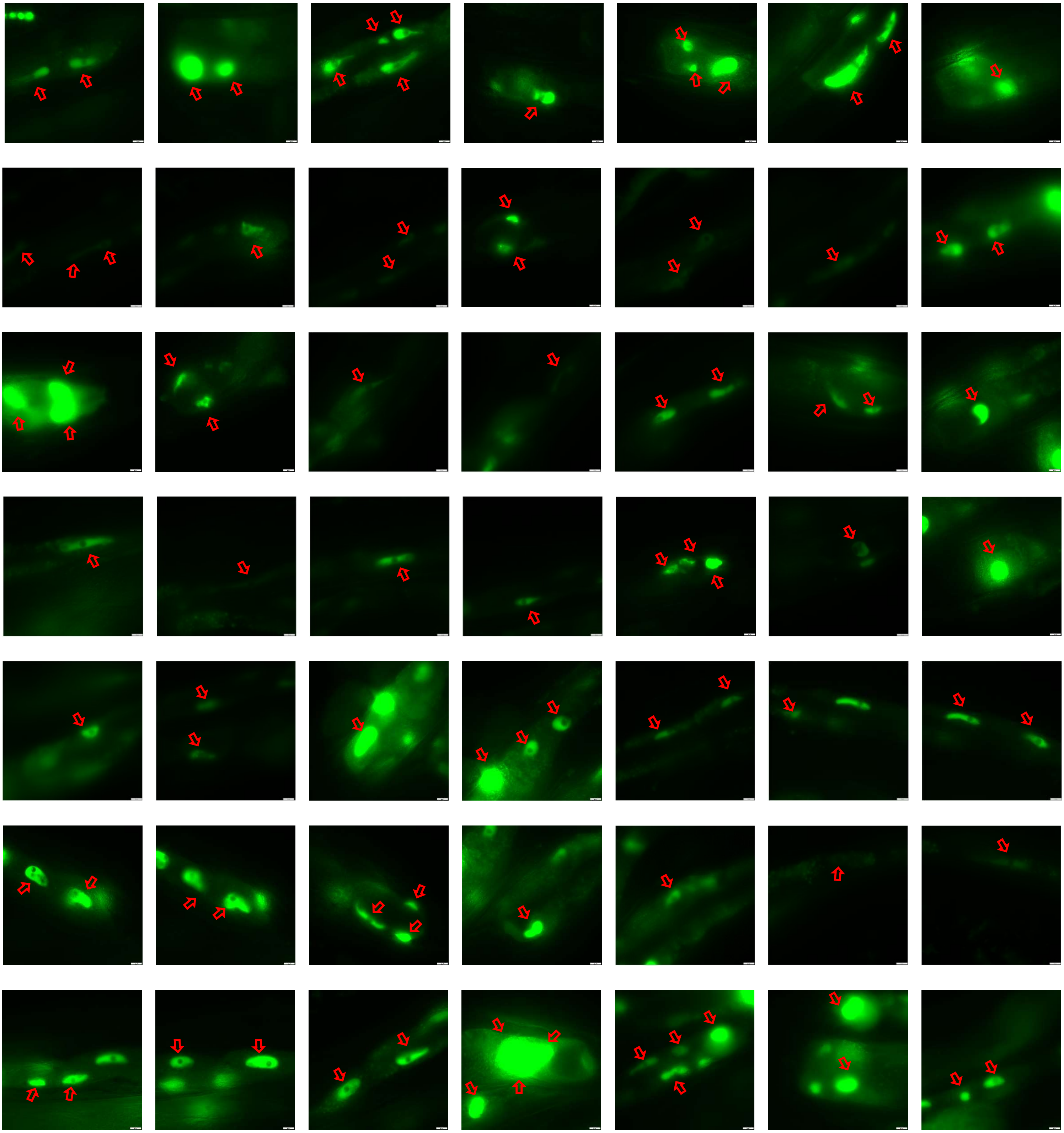

QA-Metabolites (Control-5) (1000×)

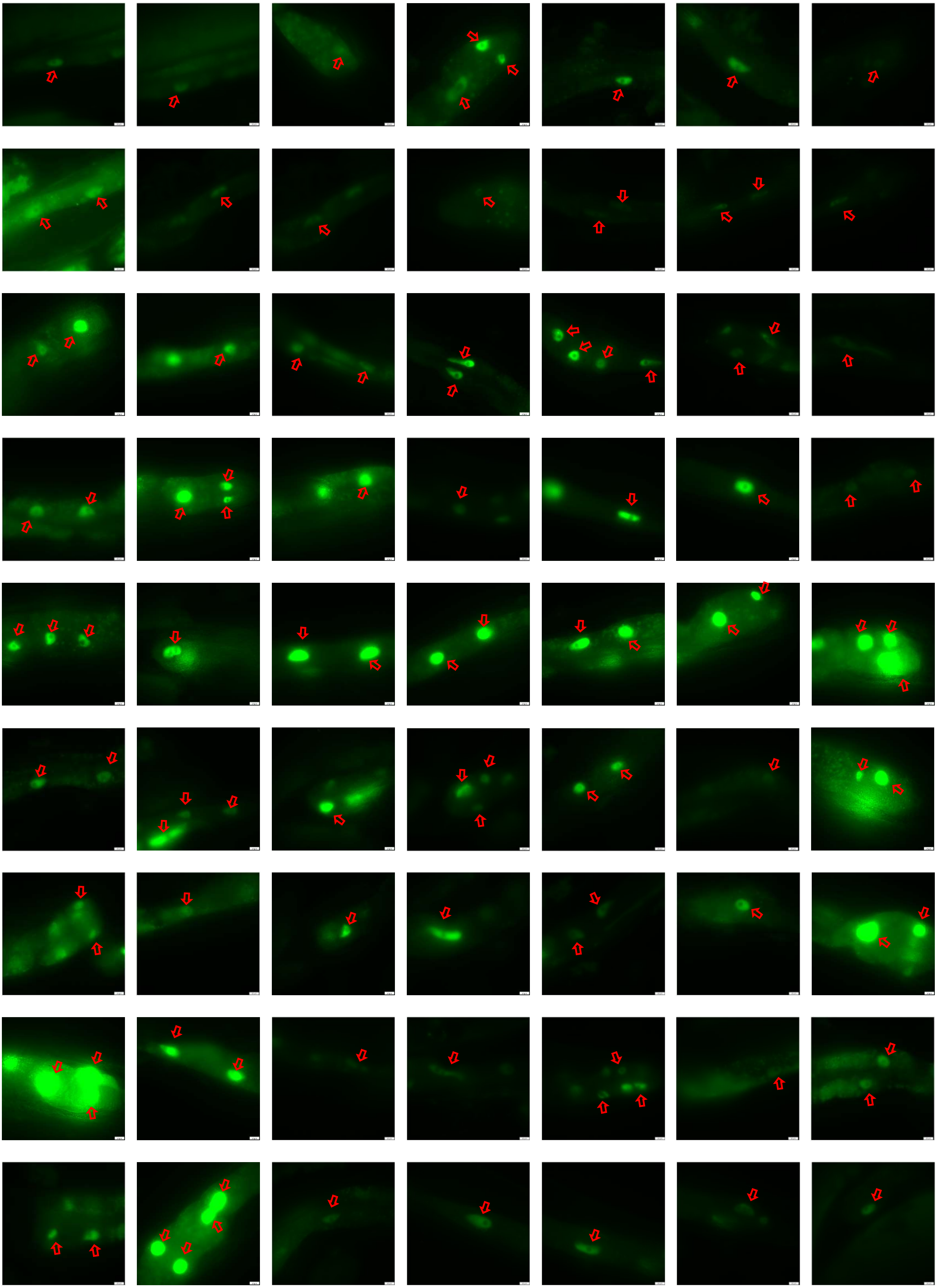

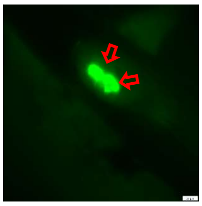

Fig. 7k-l

Control | DSS | DSS+5-HT | DSS+ LP-533401 | DSS+Trp

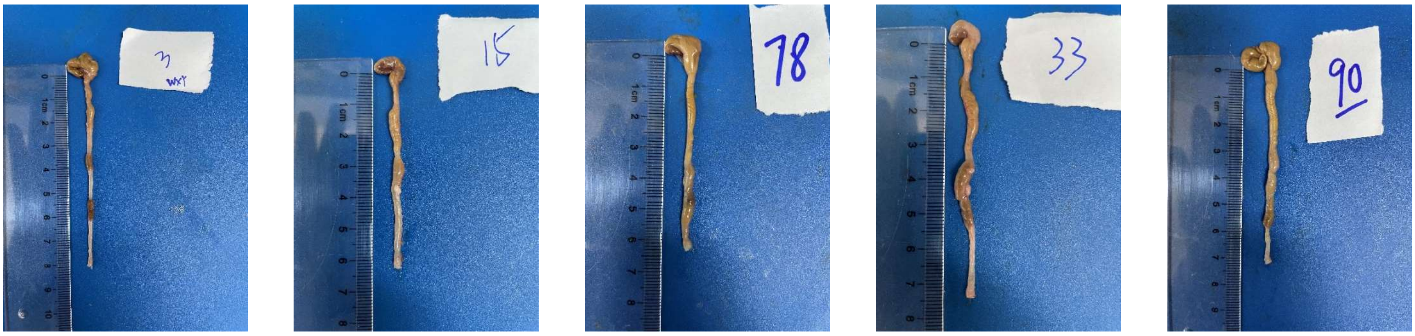

Fig. 7m

Proximal region

Control | DSS | DSS+Trp | DSS+LP-533401 | DSS+5-HT

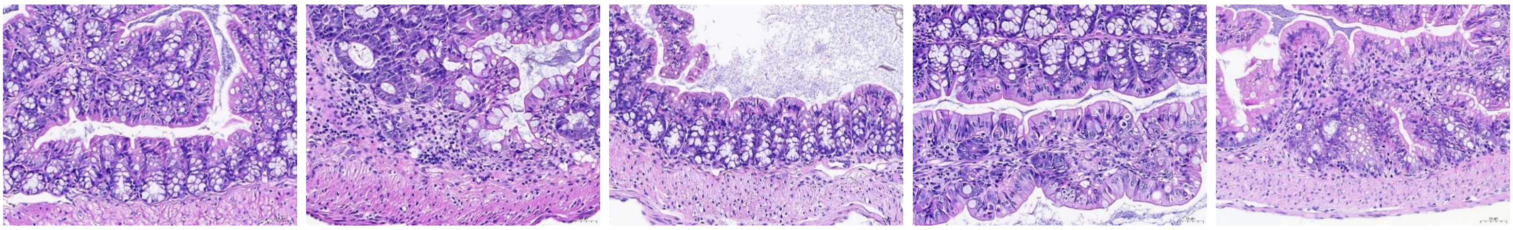

Distal region

Control | DSS | DSS+Trp | DSS+LP-533401 | DSS+5-HT

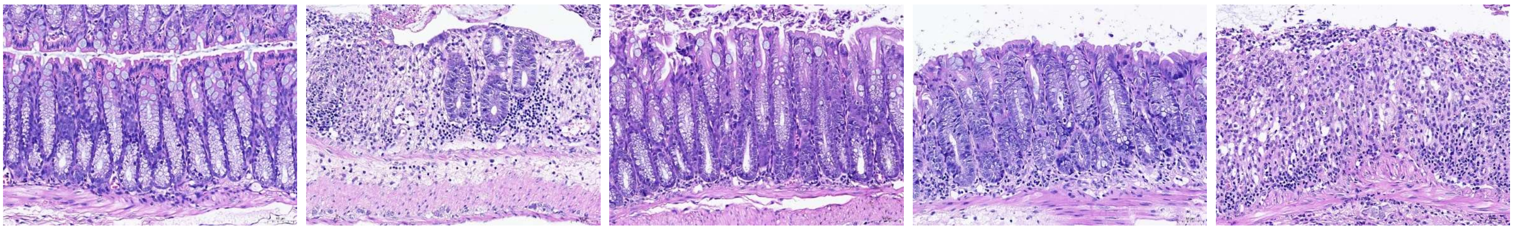

Supplement: Supplementary file 21 — Unprocessed western blots and original images. [file 43587_2024_572_MOESM21_ESM.pdf]
